# Nanoarchitectonics of Metal–Organic Framework and Nanocellulose Composites for Multifunctional Environmental Remediation

**DOI:** 10.1002/adma.202504364

**Published:** 2025-07-11

**Authors:** Ye Song, Wenkai Zhu, Chaohai Wang, Zequn Li, Ruiqi Xin, Dujuan Wu, Xiaofan Ma, Hanwei Wang, Xiangyu Wang, Song Li, Jeonghun Kim, Qingfeng Sun, Minjun Kim, Yusuke Yamauchi

**Affiliations:** ^1^ College of Chemistry and Materials Engineering Zhejiang A&F University Hangzhou 311300 China; ^2^ Henan Key Laboratory of Water Pollution Control and Rehabilitation Technology Henan International Joint Laboratory for Green Low Carbon Water Treatment Technology and Water Resources Utilization School of Municipal and Environmental Engineering Henan University of Urban Construction Pingdingshan 467036 China; ^3^ Department of Chemical and Biomolecular Engineering Yonsei University 50 Yonsei‐ro, Seodaemun‐gu Seoul 03722 Republic of Korea; ^4^ Australian Institute for Bioengineering and Nanotechnology (AIBN) The University of Queensland Brisbane Queensland 4072 Australia; ^5^ Department of Materials Process Engineering Graduate School of Engineering Nagoya University Furo‐cho, Chikusa‐ku Nagoya 464‐8603 Japan

**Keywords:** environmental remediation, MOFs, multifunctional materials, nanocellulose

## Abstract

Metal–organic frameworks (MOFs) are widely used in environmental remediation due to their unique properties. However, their practical applications are significantly limited by its powder crystal form. To address these limitations, MOFs can be integrated with abundant and sustainable biomass‐derived nanocellulose (NC) to construct processable macroscopic architectures. Herein, this review discusses recent advances in the preparation of multi‐dimensional macroscopic materials from MOFs‐NC and their applications in environmental remediation, including dye adsorption and degradation, pharmaceutical removal, heavy metal ion capture, adsorption and degradation of volatile organic compounds (VOCs), CO_2_ capture and separation, particulate matter (PM) separation, and others. A summary of two commonly used strategies for preparing MOFs‐NC composites proposes a valuable insight on how processable macroscopic architectures can be effectively achieved. Furthermore, this review provides an overview of the structure‐property‐function relationship between multi‐dimensional MOFs‐NC composites and highlights their versatile applications in the remediation of polluted environments. The mechanisms, challenges, and future prospects of the material in removing environmental pollutants are also present in detail. This review aims to guide researchers in designing high‐performance, multi‐functional, sustainable, and scalable MOFs‐NC composites for future environmental remediation.

## Graphical abstract

Nanoscale metal–organic frameworks (MOFs) and nanocellulose composites have significant potential for the multi‐functional remediation of the environment. This review focused on the synthesis strategies (in situ growth, ex situ growth, and other approaches), typical forms (aerogel, hydrogel, beads, and membrane), and applications in the removal of common pollutants (dye, pharmaceuticals, heavy metal ions, VOCs, CO_2_, PM, and others) in the environment by MOFs‐nanocellulose (MOFs‐NC) composites. The future perspectives and current challenges of MOFs‐NC composites in environmental remediation applications are also pointed out.

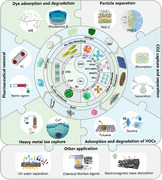

## 1 Introduction

In recent decades, with the acceleration of the industrialization process and population, a large amount of pollutants such as exhaust gas,^[^ ^1^ ^]^ wastewater,^[^ ^2^ ^]^ and solid waste^[^ ^3^ ^]^ have been discharged into the environment. These pollutants can be inorganic and organic in nature,^[^ ^4^ ^]^ and their production has caused serious damage to the ecological environment that humans rely on for survival, such as air, water, and soil.^[^ ^5^ ^]^ Therefore, it is necessary to take effective measures to restore the natural environment that has been polluted or damaged. Currently, the methods commonly used in environmental remediation include adsorption method,^[^ ^6^ ^]^ precipitation method,^[^ ^7^ ^]^ redox reactivity,^[^ ^8^ ^]^ biological remediation technologies,^[^ ^9^ ^]^ membrane separation method,^[^ ^10^ ^]^ and catalytic degradation method.^[^ ^11^ ^]^ The choice of specific method depends on the type of pollutant and the nature of the environment to be remediated. Among these methods, the treatment of pollutants by adsorption and catalytic degradation has attracted ever‐increasing attention, which is mainly attributed to their high efficiency and inexpensiveness.

The green low‐carbon strategy has become the key to escalating environmental concerns for many countries,^[^ ^12^ ^]^ and the demand for high‐performance, recyclable and eco‐friendly adsorbents and catalysts to treat environmental pollutants is becoming increasingly urgent.^[^ ^13^ ^]^ It has therefore shaped a specific research trend for researchers to use activated carbon,^[^ ^14^ ^]^ biomass carbon,^[^ ^15^ ^]^ clay,^[^ ^16^ ^]^ activated alumina,^[^ ^17^ ^]^ bio‐purifiers,^[^ ^18^ ^]^ nanomaterials,^[^ ^19^ ^]^ and photocatalysts^[^ ^20^ ^]^ to purify the polluted environment. Particularly, metal–organic frameworks (MOFs) have been widely used in environmental remediation due to their ordered porous structure, large specific surface area and abundant active sites.^[^ ^21^ ^]^ As an emerging coordination polymer, MOFs are constructed through coordination bonds between metal ions or clusters and organic linkers.^[^ ^22^ ^]^ A series of MOFs have been widely used in the field of environmental remediation, including isoreticular MOFs (IRMOFs),^[^ ^23^ ^]^ porous coordination networks (PCNs),^[^ ^24^ ^]^ materials institute lavoisier frameworks (MILs),^[^ ^25^ ^]^ zeolitic imidazolate frameworks (ZIFs),^[^ ^26^ ^]^ University of Oslo (UiO),^[^ ^27^ ^]^ multivariate (MTV),^[^ ^28^ ^]^ Hong Kong University of Science and Technology (HKUST)^[^ ^29^ ^]^ and so on. Moreover, the controllable and well‐defined porous structure of MOFs offers active sites that facilitate molecular separation, interaction and synergistic processes or effects, allowing advanced adsorption and catalytic functions. The composites or derived materials prepared by MOFs as precursors, such as metal/carbon composites,^[^ ^30^ ^]^ metal oxide materials,^[^ ^31^ ^]^ nitrogen‐doped carbonaceous materials,^[^ ^32^ ^]^ sulfur‐doped carbonaceous materials,^[^ ^33^ ^]^ and phosphorus‐doped carbonaceous materials,^[^ ^34^ ^]^ have great potential applications in the field of environmental remediation. The development of novel types of MOFs has also increased the impetus for their application in the field of environmental remediation.^[^ ^35^ ^]^ Relevant studies have shown that the purpose of improving the adsorption and catalytic performance can be achieved by adjusting the size, morphology and structure of MOFs.^[^ ^36^ ^]^

However, MOFs often exist in the form of powder, thus facing the problems associated with low recovery efficiency, potential blockage of pipelines, and secondary pollution in the actual process of using, which limits practical application.^[^ ^37^ ^]^ Particularly, in aqueous‐phase applications, the powder crystal form of MOFs present significant challenges, including poor dispersion in aqueous media potentially causing secondary pollution, difficulties in solid‐liquid separation, and low regeneration efficiency.^[^ ^37^, ^38^ ^]^ Morphological engineering is considered one of the key strategies to overcome these limitations. For pristine MOFs shaping, one commonly employed method is pelletization, wherein MOF powders are compressed into granules or tablets under high pressure. This technique can enhance mechanical stability and bulk density under appropriate pressure conditions. However, excessive pressure may cause severe aggregation and reduce the number of accessible active sites. In practical applications, binders (e.g., polyvinyl alcohol or silica gel) are often introduced to improve mechanical strength, although they may partially block the pores and consequently impair the adsorption or catalytic performance of the MOFs. Currently, the successful growth of MOFs in various substrates or support materials to synthesize formable and processable multi‐dimensional composites has become a mainstream research strategy to expand their application fields. For example, MOFs can be embedded into polymeric matrices such as polyimide or polyacrylonitrile to form mixed‐matrix monoliths. Alternatively, MOFs can be immobilized onto porous substrates, such as alumina foams or carbon fibers, through in situ growth or vapor deposition techniques to yield structured composite materials. However, these approaches require careful optimization of the interfacial compatibility between MOFs and the supporting matrix to prevent coverage or deactivation of active sites. Future efforts should focus on developing novel shaping strategies and exploring new substrate and support materials that allow for the simultaneous tuning of porosity, mechanical robustness, and structural complexity.

Among various substrate and support structure materials applicable to MOFs, nanocellulose (NC) has attracted widespread interest. In recent years, NC has emerged as one of the most valuable biomass‐derived materials due to abundant raw materials, remarkable biocompatibility, high specific surface area and degradable properties.^[^ ^39^ ^]^ According to morphology and source, it is categorized into cellulose nanofiber (CNF), cellulose nanocrystal (CNC) and bacterial cellulose (BC).^[^ ^40^ ^]^ Among them, CNC and CNF are extracted from cellulose‐rich biomass materials using a top‐down strategy.^[^ ^41^ ^]^ On the contrary, BC is synthesized by bacterial microorganisms through a bottom‐up strategy using low‐molecular‐weight carbon sources as raw materials.^[^ ^42^ ^]^ NC has been widely used in the fields of adsorption,^[^ ^43^ ^]^ biomedical,^[^ ^44^ ^]^ food packaging^[^ ^45^ ^]^ and energy storage.^[^ ^46^ ^]^ As a 1D nano‐polymer material, NC has a large number of active groups on surface, which provides unlimited possibilities for modification reactions such as carboxylation, sulfonation and grafting. The modified NC is normally used as an excellent carrier for constructing novel composites due to the advantages of facile processing.^[^ ^47^ ^]^ Therefore, the innovative design of MOFs with NC as substrate material can improve flexibility and broaden applications.

**Figure 1:**
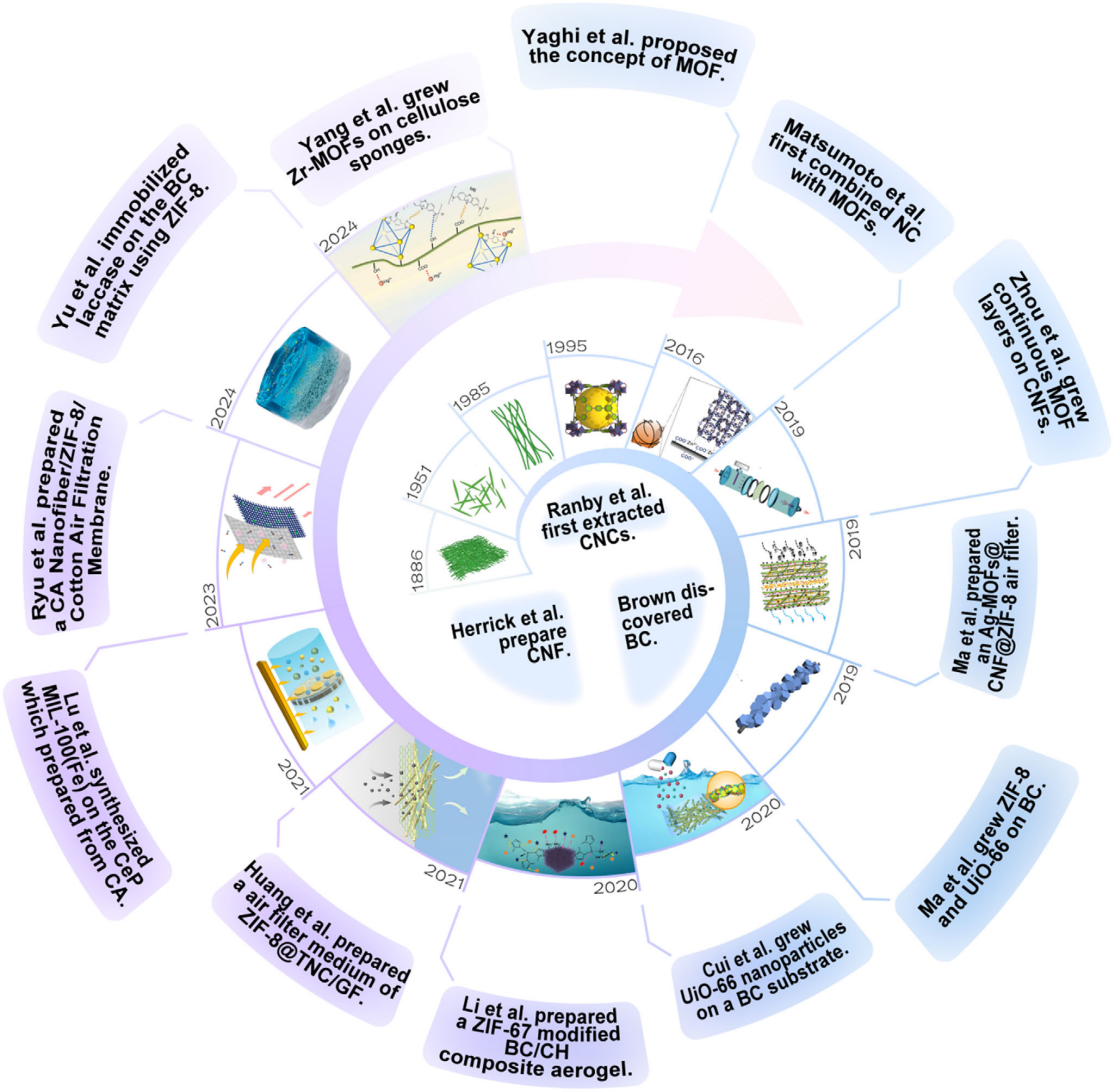
Timeline of key milestones in MOFs‐NC development by representative examples. Reproduced with permission.^[^ ^40^ ^]^ Copyright 2022, Springer Nature B.V. Reproduced with permission.^[^ ^48^ ^]^ Copyright 2016, Wiley‐VCH Verlag GmbH & Co. KGaA. Reproduced with permission.^[^ ^49^ ^]^ Copyright 2019, Wiley‐VCH Verlag GmbH & Co. KGaA. Reproduced with permission.^[^ ^50^ ^]^ Copyright 2019, Elsevier. Reproduced with permission.^[^ ^51^ ^]^ Copyright 2019, Elsevier. Reproduced with permission.^[^ ^52^ ^]^ Copyright 2020, Elsevier. Reproduced with permission. Copyright 2020, Elsevier B.V. Reproduced with permission.^[^ ^54^ ^]^ Copyright 2021, ACS. Reproduced with permission.^[^ ^56^ ^]^ Copyright 2021, Elsevier Ltd. Reproduced with permission.^[^ ^57^ ^]^ Copyright 2023, MDPI. Reproduced with permission.^[^ ^58^ ^]^ Copyright 2024, Elsevier. Reproduced with permission.^[^ ^59^ ^]^ Copyright 2024, Elsevier Ltd.

**Figure 2:**
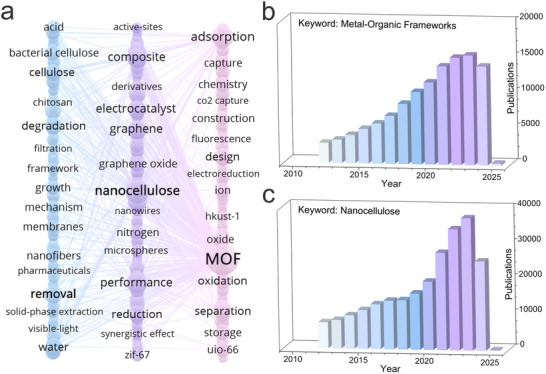
a) Top keywords used about nanoarchitectonics of MOFs‐NC composites for multifunctional environmental remediation. Number of publications with the relevant keywords b) MOFs and c) NC (Web of Science, from January 1, 2010 to March 1, 2025).

**Figure** **1** provides an overview of the development of MOFs‐NC composites. Since the discovery of CNC, BC, and CNF, the unique properties of NC have become increasingly evident.^[^ ^48^, ^49^, ^50^, ^51^, ^52^, ^53^, ^54^, ^55^, ^56^, ^57^, ^58^, ^59^ ^]^ The concept of MOFs was first introduced in 1995,^[^ ^59^ ^]^ and their high specific surface area, adjustable structure, and versatile mechanisms of action have demonstrated significant potential for applications in environmental remediation. However, due to their powder form, MOFs have not been effectively utilized in practical applications. In 2016, Matsumoto and Kitaoka synthesized MOFs with carboxylate groups in situ on CNF prepared via the 2,2,6,6‐tetramethylpiperidin‐1‐oxyl (TEMPO) oxidation method.^[^ ^48^ ^]^ This approach addressed the permeability and selectivity of the composites for CO_2_, highlighting the advantages in environmental remediation. Ma et al. subsequently loaded MOFs onto BC, significantly inhibiting the aggregation of individual MOF nanoparticles.^[^ ^51^ ^]^ In the past five years, the development of MOFs‐NC composites has seen a significant progress, with ongoing research into the performance and mechanisms of MOFs and NC (**Figure** **2**a). For instance, Cui et al. uniformly coated UiO‐66 nanoparticles onto the 3D network structure of BC, ensuring abundant active sites in the material.^[^ ^52^ ^]^ The Zr^4^⁺ metal centers in UiO‐66 formed strong coordination bonds with the oxygen and nitrogen atoms in tetracycline (TC) molecules, thereby significantly enhancing chemical adsorption capacity and exhibiting higher adsorption efficiency compared to physical adsorption. Lu et al. promoted the fixation of hydrophilic MIL‐100 (Fe) crystals by deacetylating cellulose acetate (CA) and partially leaching polyvinylpyrrolidone (PVP) porogen, thereby enabling efficient oil emulsion separation, dye degradation, and Cr⁶⁺ reduction.^[^ ^55^ ^]^ By 2024, Yu et al. introduced a visible light‐driven biomass photoenzyme coupling system (BCPC) that facilitated electron transfer in the catalytic process through the close coupling of photocatalysts and enzyme catalysts, reducing hole recombination and further enhancing photocatalytic efficiency.^[^ ^57^ ^]^ This surge in development is also reflected in the increasing number of publications in the last fifteen years. Figure 2b,c show the number of articles published in the Web of Science database from January 1, 2010, to December 31, 2025, indicating a consistent upward trend in the number of publications based on “MOFs” and “NC” as search keywords.

**Figure 3:**
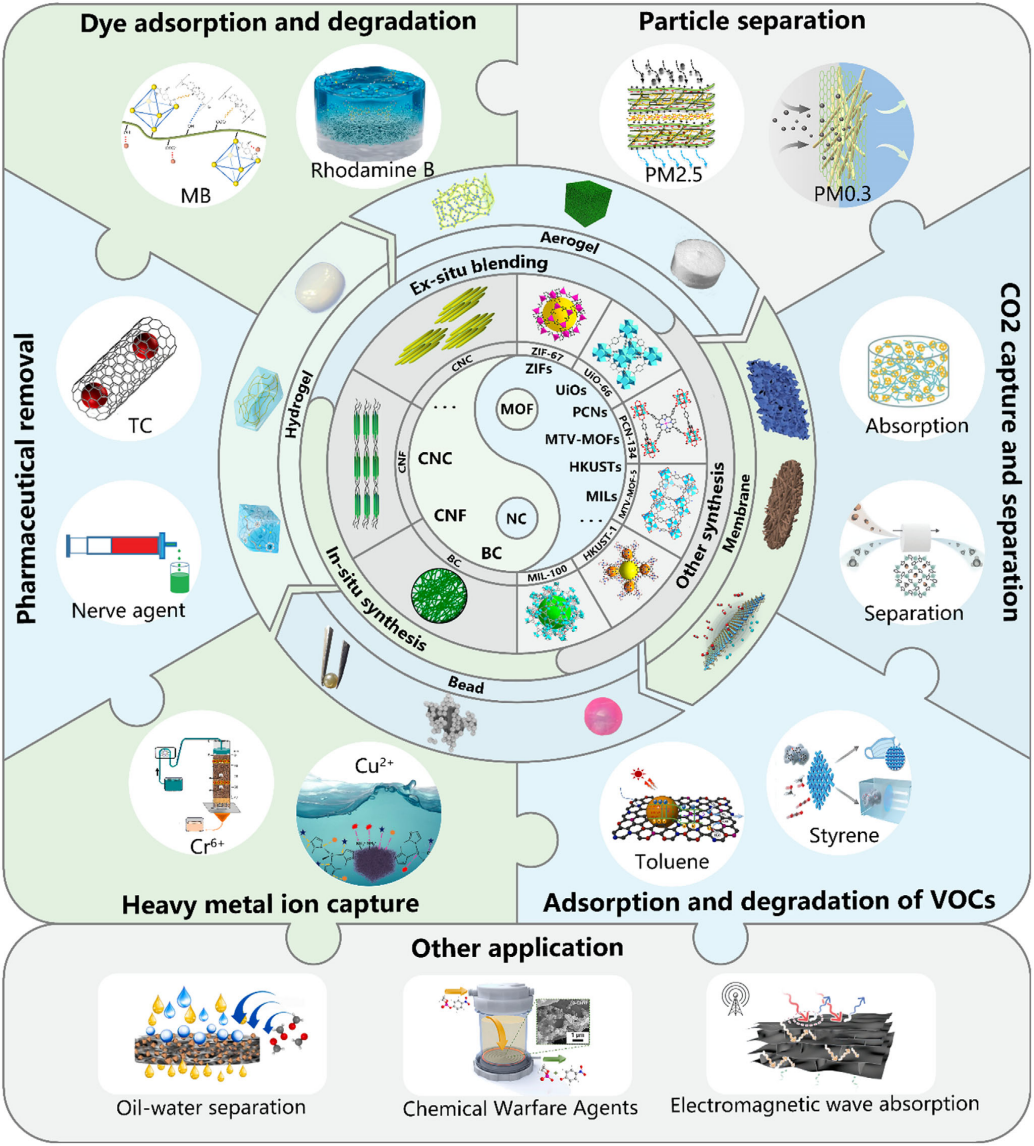
The forms of the MOFs‐NC composites generated from different types of MOFs and NC via various states and preparation methods, as well as their applications in environmental remediation.

In recent years, the use of MOFs‐NC composites in environmental remediation is gaining momentum, paving the way for significant advancements in the field.^[^ ^60^ ^]^ So far, a variety of MOFs‐NC composites have been prepared by combining various kinds of MOFs (e.g., ZIF‐67,^[^ ^61^ ^]^ ZIF‐8,^[^ ^62^ ^]^ ZIF‐L,^[^ ^63^ ^]^ MIL‐100,^[^ ^64^ ^]^ HKUST‐1)^[^ ^65^ ^]^ and NC (CNC, CNF and BC) and applied to the field of environmental remediation (**Figure** **3**). Currently, the conversion of MOFs into multi‐scale macroscopic materials by compositing with NC can be done by in situ growth, ex situ growth and other strategies. Subsequently, the material can be dried (e.g., oven drying, freeze drying, vacuum drying), carbonized, filtered or cross‐linked to form aerogel,^[^ ^66^ ^]^ hydrogel,^[^ ^67^ ^]^ membrane^[^ ^68^ ^]^ or bead.^[^ ^69^ ^]^ Due to the favorable synergistic interaction between MOFs and NC, the resulting composites possess large specific surface area, hierarchical porous structure, superior mechanical and catalytic properties. The excellent physical and chemical properties of these multi‐type MOFs‐NC composites allow interactions with environmental pollutants through hydrogen bonding, electrostatic interactions, and complexation, therefore, achieving the purpose of environmental remediation.^[^ ^70^ ^]^

With the rapid development of MOFs and NC, it is necessary to summarize the preparation methods, physical‐chemical properties, applications, and mechanisms of their composites in the field of environmental remediation. In recent years, several reviews have summarized the use of MOFs or NC and MOFs‐NC in environmental remediation or other fields.^[^ ^71^ ^]^ For instance, Zhang et al. provided a comprehensive review of the wide applications, synthesis methods, challenges, and future trends of MOFs in the field of environmental remediation.^[^ ^71^ ^]^ Similarly, Yohannes et al. introduced the emerging applications of MOFs in environmental remediation, particularly in water treatment and air purification.^[^ ^71^ ^]^ Musarurwa et al. overviewed the application of smart MOFs (thermo‐responsive MOFs, light‐responsive MOFs and magnetic responsive MOFs) during environmental remediation.^[^ ^71^ ^]^ Lu et al. summarized the preparation approaches, and properties of MOF/cellulose nanomaterials composites.^[^ ^71^ ^]^ Besides, they also focused on the emerging applications of the composites, including water remediation, air purification, biomedical applications, electrochemical energy storage and conversion devices, and other emerging applications. Mai et al. highlighted the recent advances in MOFs‐NC composites, and mainly reviewed its applications in sewage treatment, gas separation, energy storage, and biomedicine.^[^ ^71^ ^]^ Although the above review summarizes the latest developments based on MOFs and NC, there are few reviews on the fabrication methods of MOFs‐NC composites and their targeted applications in the field of environmental remediation. Therefore, this review will outline the strategy for the synthesis of MOFs‐NC based macroscopic composites and explore the structure‐property‐function relationships and applications in the field of environmental remediation (i.e., dye adsorption and degradation, pharmaceutical removal, heavy metal ion capture, adsorption and degradation of volatile organic compounds (VOCs), CO_2_ capture and separation, and PM separation). This review will highlight the importance and diversity of MOFs‐NC composites in the field of environmental remediation, and facilitate their future research and development in the broader environmental field.

## 2 Preparation Strategies of MOFs‐NC Composites

### 2.1 Family of Cellulose

**Figure 4:**
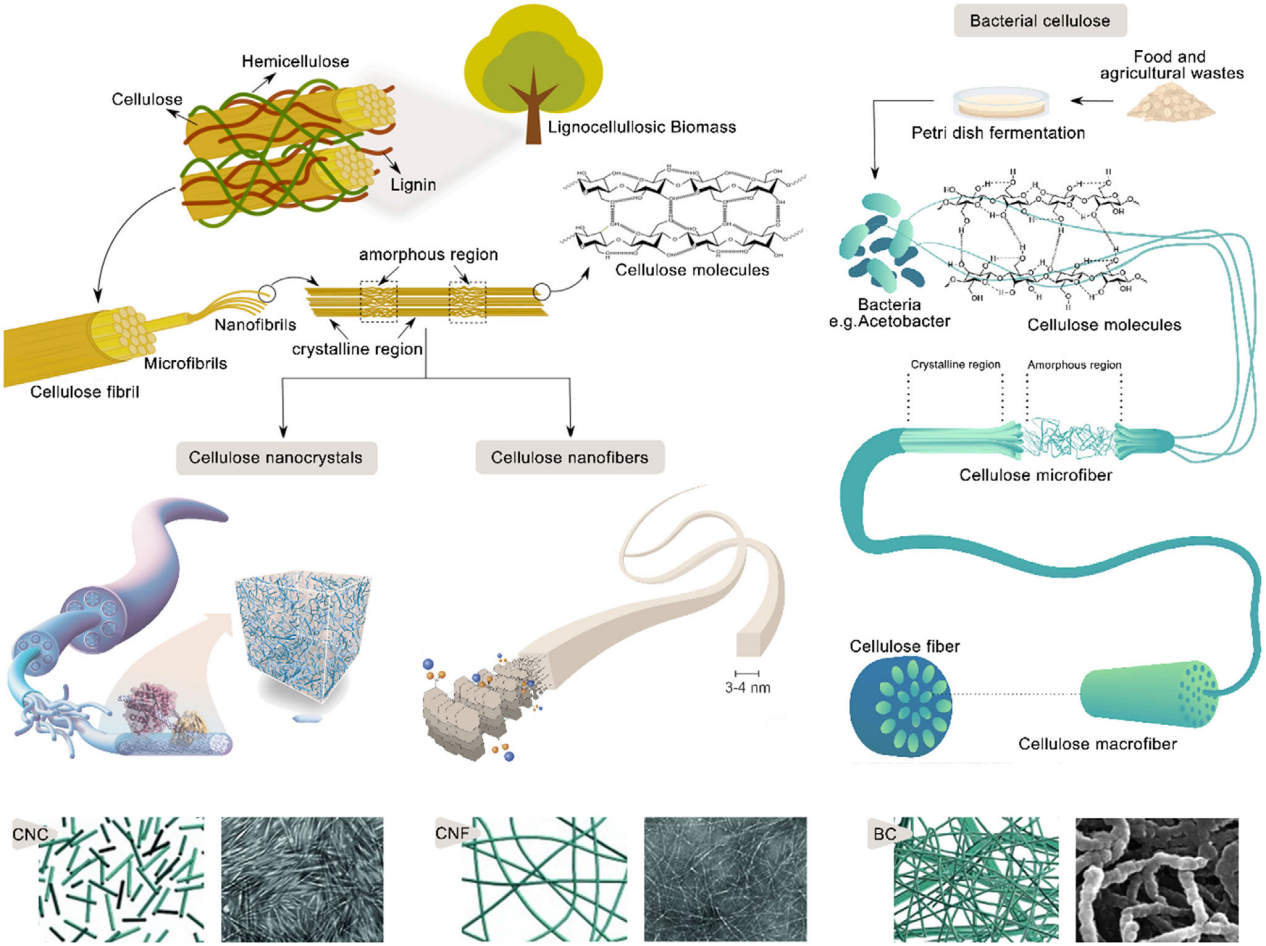
The sources and hierarchical structure of CNC, CNF, BC: CNC, and CNF derived from lignocellulosic biomass (containing cellulose, hemicellulose, and lignin) and BC synthesized via fermentation. Highlighting their distinct morphology and nanoscale features with structural schematic diagram and SEM images. Reproduced with permission.^[^ ^86^ ^]^ Copyright 2020, MDPI. Reproduced with permission.^[^ ^87^ ^]^ Copyright 2020, Springer Nature. Reproduced with permission.^[^ ^88^ ^]^ Copyright 2023, Wiley‐VCH GmbH. Reproduced with permission.^[^ ^89^ ^]^ Copyright 2023, Elsevier Ltd. Reproduced with permission.^[^ ^90^ ^]^ Copyright 2023, the Royal Society of Chemistry.

NC is derived from renewable natural polymeric materials that are abundant on earth, such as wood, cotton, root vegetables, rice straw, bacteria, marine animals, and algae.^[^ ^47^ ^]^ Attributed to its fascinating physical and chemical properties such as nanoscale size, high specific surface area, remarkable hydrophilicity, biodegradability, high tensile strength, and stiffness, it has received ubiquitous attention. NC is often extracted from the hierarchical structure of cellulose‐rich raw materials.^[^ ^72^ ^]^ Due to the hierarchical assembly of cellulose molecular chains, different types of NC can be extracted from different sources of raw materials, including CNC, CNF and BC (**Figure** **4**).^[^ ^40^ ^]^ The commonly used preparation methods include mechanical treatment, chemical method, and biological method. Furthermore, the methods used to prepare different types of NC are also different. Generally, mechanical treatment means to rely on ball milling,^[^ ^73^ ^]^ high pressure homogenization,^[^ ^74^ ^]^ high power ultrasound,^[^ ^75^ ^]^ microfluidization,^[^ ^76^ ^]^ steam blasting,^[^ ^77^ ^]^ freezing, and crushing to produce high strength mechanical external forces (such as impact force, shear force and friction, etc.) to destroy the internal structure of cellulose and extract nano‐sized NC. The chemical method refers to the use of chemical reagents to degrade the amorphous region of cellulose to break the β‐1,4 glycosidic bonds between the glucose units in the cellulose molecular chain, thereby producing NC with high crystallinity. Common chemical methods include inorganic acid hydrolysis,^[^ ^78^ ^]^ organic acid hydrolysis,^[^ ^79^ ^]^ deep eutectic solvent,^[^ ^80^ ^]^ acid vapor method, TEMPO oxidation,^[^ ^81^ ^]^ ionic liquid,^[^ ^82^ ^]^ and periodate oxidation.^[^ ^83^ ^]^ Moreover, biological preparation of NC mainly includes two methods: enzymatic digestion and microbial synthesis. Enzymatic hydrolysis is a method of hydrolyzing the amorphous region of cellulose fibers and preserving the crystalline region by using the active component of glucosanase (EG) in cellulase.^[^ ^84^ ^]^ The microbial synthesis is the fermentation of acetobacterium, rhizobium and agrobacterium to produce BC.^[^ ^85^ ^]^ Among them, mechanical treatment and chemical methods are commonly used for the extraction and separation of CNF and CNC, while BC is usually produced by biological methods.^[^ ^86^, ^87^, ^88^, ^89^, ^90^ ^]^

The morphology, size, crystallinity, thermal stability, and dispersibility of each NC depend on the source of cellulose raw materials, separation and processing conditions, and the methods involved in pre‐treatment or post‐treatment.^[^ ^91^ ^]^ The features of CNC, CNF, and BC are displayed in **Table** **1**.^[^ ^92^, ^93^, ^94^, ^95^, ^96^, ^97^, ^98^ ^]^ CNC, also known as cellulose whiskers, cellulose nanowhiskers (CNW), cellulose crystals, and nanocrystalline cellulose (NCC), is usually rod or whisker shaped with an average width of 3–50 nm and length of 50–500 nm, and its crystallinity is 54–90%. CNC also has high axial stiffness (105–168 GPa), high Young's modulus (20–50 GPa), high tensile strength (≈9 GPa), low thermal expansion coefficient (≈0.1 ppm K^−1^), high thermal stability (≈260°C), high aspect ratio (10–70), low density (1.5–1.6 g cm^−3^), lyotropic liquid crystal behavior and shear thinning phenomenon.^[^ ^98^ ^]^ CNC has been widely used as a filler to reinforce nanocomposites, and it has also gained attention in the fields of biomedicine and food packaging due to its safety and efficacy.^[^ ^99^ ^]^ Furthermore, other common names for CNF include nanofibrillated cellulose (NFC), and microfibrillated cellulose (MFC). CNF is a long soft chain with micron length located in the fiber cell wall, consisting of a bundle of long cellulose chain molecules. CNF is a nanofiber with a diameter of 5–60 nm and a length of several microns entangled, including alternating crystalline regions and amorphous regions. Therefore, it has stronger toughness, bending resistance and impact resistance compared with CNC.^[^ ^100^ ^]^ Due to the excellent mechanical properties of CNF, it has been widely used in packaging, coatings, electronic products, composites, pharmaceuticals and other fields.^[^ ^101^ ^]^ BC is an extracellular insoluble polysaccharide secreted by microbial metabolism, which has many unique properties such as sophisticated network structure, excellent mechanical strength, outstanding biocompatibility and biodegradability.^[^ ^102^ ^]^ BC and plant cellulose have the same chemical structure, which is composed of D‐glucopyranose monomers connected by β‐1,4‐glycosidic bonds. There are 3 hydroxyl groups on each glucose monomer, and they can form hydrogen bonds with hydroxyl groups of adjacent glucose monomers or molecular chains. This bonding not only enhances the mechanical properties of BC, but also allows the molecules to be closely aligned with each other, resulting in an ordered and highly crystalline region. BC is one of the hottest international biomaterials in nowadays, and the research on it mainly focuses on the high value‐added medical biomaterials, such as tissue engineering scaffolds, food industry, artificial skin, biomedical and drug carriers.^[^ ^103^ ^]^

**Table 1:** Comparison table of the features of various NC.

NC Properties	CNC	CNF	BC	References
Form	Rod‐like or filamentous	Filamentous or reticulate	3D mesh structure	[92, 98]
Size	Average width: 3–50 nm; Length: 50–500 nm.	Diameter: 5–60 nm, Length: several microns.	Length: ≈µm, width: 25–86 nm.	[92, 99]
Crystallinity	54–90%	60–80%	60–90%	[92, 100]
Axial stiffness	105–168 GPa	2–7.7 GPa	/	[92, 101]
Young's modulus	20–50 GPa	140–161 GPa	15–30 GPa	[92, 100, 101]
Tensile strength	9 GPa	/	20–300 MPa	[92, 99]
Coefficient of thermal expansion	0.1 ppm K^−1^	5–7 ppm K^−1^	/	[92, 102]
Thermal stability	≈260°C	200°C	190–580°C	[92, 103]
Length‐to‐diameter ratio	10–70	>800	/	[92, 103]
Density	1.5–1.6 g cm^−3^	≈1.6 g cm^−3^	/	[92, 100]

NC contains a large number of overly hydrophilic hydroxyl groups and a dense network of intermolecular hydrogen bonds, making it highly hydrophilic and prone to gelation, which limits its further application. Therefore, modification of NC can reduce its hydrophilicity and endow it with new properties, thereby expanding its application range. Currently, common NC modification strategies include physical adsorption, oxidation, etherification, esterification, amidation, hydrophobicity, non‐covalent surface modification, and graft copolymerization.^[^ ^104^ ^]^ The modification method of physical adsorption is easy to operate, the whole production process is simple, and the integrity of NC can be well preserved. However, NC and the modified substance are combined in the form of weak van der Waals force and hydrogen bond, so the product is weak in shear force resistance, and it is easy to dissociate the adsorbed substance under the action of external force, resulting in instability.^[^ ^105^ ^]^ According to the abundant active groups on the surface of NC, a variety of functional groups can be grafted to chemically modify, thereby endowing it with new functional properties and preparing more promising NC‐based functional materials.^[^ ^104^, ^106^ ^]^ The modified NC has both the functions of the original cellulose and the properties of the modified reactants themselves, which can be used in many fields.

### 2.2 Typical MOFs Suitable for MOFs‐NC Composites

**Figure 5:**
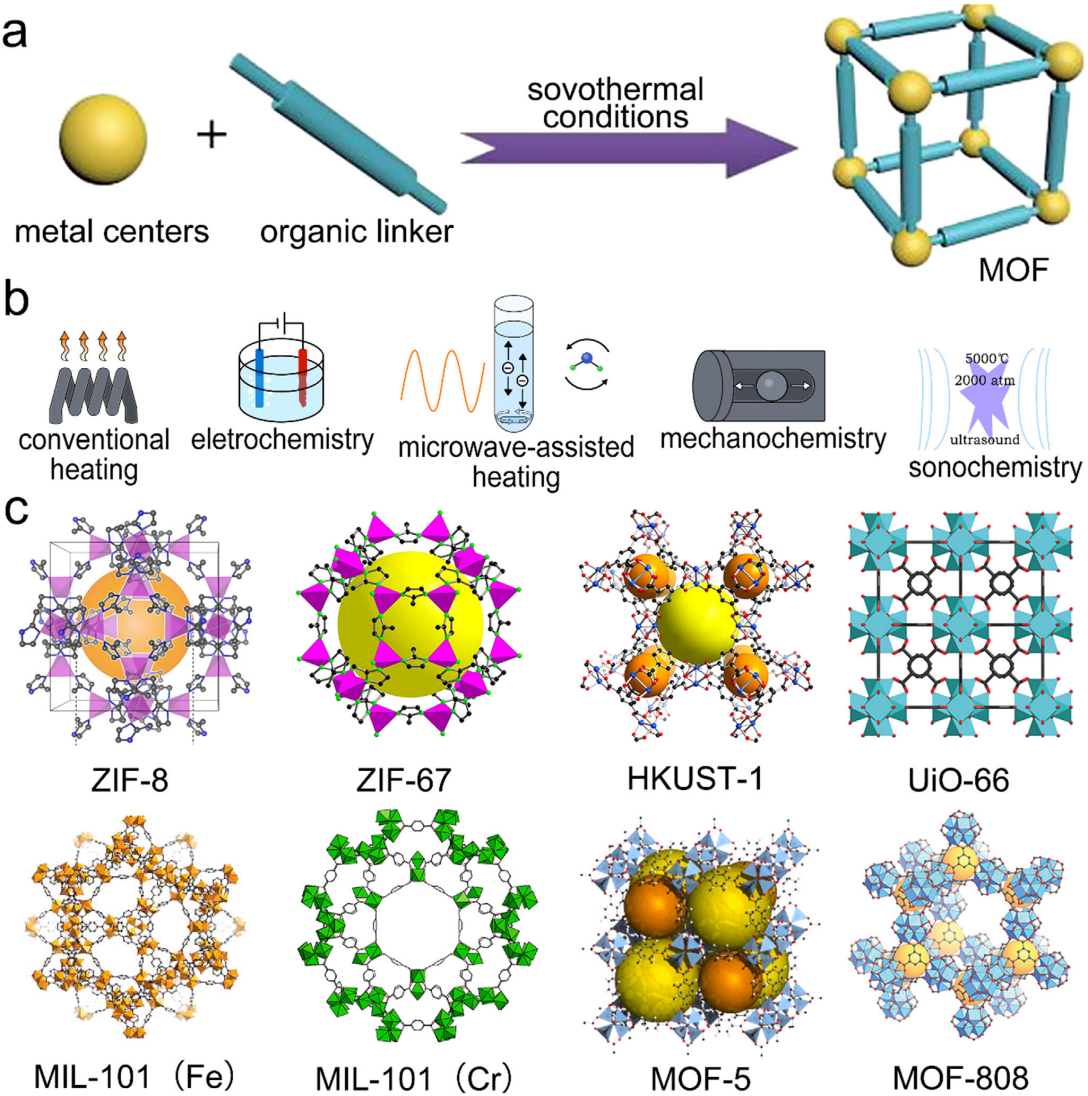
a) The preparation process and b) common preparation strategies of MOFs. Reproduced with permission.^[^ ^107^ ^]^ Copyright 2017 Elsevier. Reproduced with permission.^[^ ^109^ ^]^ Copyright 2011 ACS. c) Typical MOFs for MOFs‐NC composites. Reproduced with permission.^[^ ^115^ ^]^ Copyright 2021 Springer Nature. Reproduced with permission.^[^ ^117^ ^]^ Copyright 2021 Elsevier. Reproduced with permission.^[^ ^127^ ^]^ Copyright 2024 Elsevier. Reproduced with permission.^[^ ^128^ ^]^ Copyright 2018 Wiley‐VCH GmbH. Reproduced with permission.^[^ ^129^ ^]^ Copyright 2018 ACS.

MOFs are formed by extending and expanding different types of organic ligands to form framework networks by using different types of metal ions as connectors (**Figure** **5**a).^[^ ^107^ ^]^ Furthermore, the secondary structural units that make up MOFs are small structural units formed by combining coordination groups with metal ions, which to a certain extent determine the final topology of the material skeleton. These factors have led to the fabrication of MOFs with adjustable pore sizes, diverse structures, many open sites, ease of modification, great variety, wide source of raw materials, and a huge number of moieties.^[^ ^108^ ^]^ To date, various types of MOFs have been developed using different synthetic strategies (Figure 5b), such as hydrothermal/solvothermal, electrochemical, mechanochemical, microwave, and ultrasonic.^[^ ^109^ ^]^ Although the fascinating structure of MOFs has attracted widespread research interest, their powdery crystalline structure creates the disadvantage of poor processability and difficulty in recycling. Moreover, to the best of the authors’ knowledge, typical MOFs suitable for MOFs‐NC composites are limited to a few major MOF families, which mainly depends on the required hydrothermal temperature, chemical reagents, mechanical stability, simple synthesis conditions, and lower cost. The typical MOFs suitable for MOFs‐NC composites include ZIF family, MIL series, UiO type and others (Figure 5c). And the typical MOFs used in MOFs‐NC composites and corresponding topologies are shown in **Table** **2**.

**Table 2:** Summary of representative MOFs suitable for MOFs‐NC composites.

MOFs		Metal	Ligands	Topology	References
ZIF family	ZIF‐8	Zn	EMIM	SOD	[115]
	ZIF‐L	Zn	Hmim	SOD	[116]
	ZIF‐67	Co	2‐MIM	SOD	[117]
	ZIF‐90	Zn	ICA	SOD	[49]
MIL series	MIL‐100 (Fe)	Fe	H_3_BTC	MTN	[118]
	Al‐MIL‐53	Al	TPA	–	[119]
UiO series	UiO‐66	Zr	BDC	FCU	[120]
	UiO‐66‐NH_2_	Zr	NH_2_‐BDC	FCU	[121]
Others	HKUST‐1	Cu	H_3_BTC	TBO	[122]
	MOF‐74	Zn	DHTA	ROD	[123]
	Ln‐MOFs	Ln	H_3_imdc	BTB	[124]
	MOF‐808	Zr	BTCA	SPN	[125]

The uniform distribution of MOF particles enhances the overall mechanical strength of the material, while the unique 3D crystal structure provides additional mechanical support and facilitates the formation of larger, wider pores.^[^ ^110^ ^]^ The resulting material retains the thermal and chemical stability of MOFs, enabling to maintain performance during the adsorption‐desorption cycle. Consequently, the material can be regenerated through methods such as physical extrusion or chemical elution, while maintaining a high adsorption capacity during subsequent cycles.^[^ ^111^ ^]^ The diverse pore architectures of MOFs arise from the combination of organic linkers of varying lengths and metal clusters with different geometrical configurations. This structural diversity results in a wide range of topologies, which in turn influences pore size distribution. As a result, MOFs possess significantly higher specific surface areas compared to traditional adsorbents. Based on this, MOFs offer abundant adsorption sites and channels for dye molecule uptake.^[^ ^112^ ^]^ By precisely tuning the size of organic linkers or metal nodes, pore size can be controlled, enabling selective capture of specific pollutants in complex contaminant matrices. For instance, introducing structural defects or substituting metal ions (e.g., replacing Zr with Ti) can enhance CO_2_ adsorption capacity.^[^ ^113^ ^]^ In addition to size‐selective sieving, MOFs selectivity can also be governed by specific chemical interactions, including electrostatic forces, hydrogen bonding, π–π stacking, and coordination bonding. Functional group modifications on organic linkers (e.g., ─NH_2_, ─SO_3_H, ─OH) can significantly enhance affinity toward target pollutants. Likewise, tailoring the coordination environment of metal centers improves adsorption selectivity. Pre‐loading MOF pores with specific guest molecules (e.g., ionic liquids, crown ethers) further enhances selectivity via host‐guest interactions. Incorporating nanoparticles (e.g., Pd, TiO_2_) or polymers into MOF pores can also synergistically enhance catalytic or adsorption properties. For example, Ag‐loaded ZIF‐8 demonstrates both efficient adsorption and photocatalytic degradation of organic contaminants. Additionally, their active sites enable chemical and physical adsorption, further enhancing their adsorption performance. Moreover, MOFs promote the generation and transfer of electrons. For instance, Jia et al. biomineralized ZIF‐8 and introduced chitosan (CS) and NC unidirectional conversion into aerogels, creating anisotropic hybrid aerogels with highly oriented porous structures.^[^ ^114^ ^]^ ZIF‐8 served as an electronic medium, facilitating electron transfer from the highest occupied molecular orbital (HOMO) to the lowest unoccupied molecular orbital (LUMO). This enhanced the generation of electrons in water, promoted their transfer to dye molecules, and improved adsorption performance.

Li et al. developed a novel adsorbent (U‐EDTACCA) with fibrous, columnar, and lamellar pore structures via directional freeze‐drying.^[^ ^111^ ^]^ UiO‐66 in the composite supported the formation of anisotropic pore structures, which enhanced adsorption efficiency and accelerated adsorption kinetics. The electronic band structure of MOFs can be finely tuned by altering the organic ligands or metal centers. Such MOFs can generate reactive oxygen species (ROS), such as hydroxyl radicals (•OH) and superoxide anion radicals (•O_2_⁻), under light irradiation, enabling the degradation of organic pollutants like phenol and antibiotics. Iron‐based MOFs, such as MIL‐53(Fe), can catalyze Fenton‐like reactions to efficiently degrade dyes. Some MOFs also enhance the antibacterial properties of materials. For example, Yang et al. prepared HKUST‐1@lignocellulose nanofibrils (LCNFs) using a green in situ strategy and subsequently mixed HKUST‐1@LCNFs with a CA casting solution to prepare a composite ultrafiltration membrane with superior performance.^[^ ^110^ ^]^ The Cu^2+^ ions released by HKUST‐1 acted as oxidants, disrupting bacterial cell walls and causing microbial death. As a result, the blend membrane demonstrated excellent antibacterial properties, while the presence of hydrophilic functional groups further improved the pure water flux, reaching 207.32 L m^−2^ h^−1^.

Compared to conventional adsorbents, MOFs exhibit distinct advantages in terms of structural and functional attributes (**Table** **3**). Their intrinsic high surface area and tunable porosity contribute not only to high adsorption capacities but also to precise molecular sieving. For example, ZIF‐8 features a specific surface area up to 1200 m^2^ g^−1^, significantly outperforming traditional materials like activated carbon.^[^ ^115^ ^]^ ZIF‐90 demonstrates remarkable selectivity and permeability in gas separation to the level that is rarely achieved by other adsorbents, thus effectively distinguishing CO_2_ from CH_4_.^[^ ^49^ ^]^ More importantly, MOFs overcome the functional limitations of traditional adsorbents.^[^ ^116^ ^]^ For instance, ZIF‐67 not only adsorbs pollutants but also exhibits photocatalytic activity, allowing it to degrade adsorbed species into harmless byproducts.^[^ ^117^ ^]^ Some MOFs possess environmentally responsive behaviors, such as pH‐sensitive MIL‐100, which can dynamically regulate urea release‐features seldom observed in conventional bio‐adsorbents.^[^ ^118^ ^]^

**Table 3:** Advantages and disadvantages of different MOFs type in environmental remediation.

MOFs	Advantages	Disadvantages	Refs.
ZIF‐8	High adsorption capacity; Chemical stability; Adjustable functionality; Diversification of applications.	Water stability needs to be improved; Limited selectivity for adsorption; Complex recycling and reuse; Insufficient mechanical stability.	[115]
ZIF‐L	High specific surface area and layered structure; Designability; Antibacterial activity; Controlled synthesis.	Poor structural stability; Insufficient recycling capacity; Limited selectivity for adsorption; Insufficient mechanical stability.	[116]
ZIF‐67	High specific surface area and porosity; Visible light response, Good chemical stability, Excellent catalytic properties.	Insufficient aqueous phase stability; Potential environmental toxicity of cobalt; Poor reusability.	[117]
ZIF‐90	Rich functional groups; High gas selectivity; High permeability; Good chemical reaction activity.	High cost; Poor water stability; Limited environmental adaptability; Lower mechanical strength.	[49]
MIL‐100 (Fe)	Green and low‐toxicity; Highly sustainable; Strong chemical stability; Multi‐stage hole structure; Good Fenton catalytic activity High specific surface area; pH response; Enhanced moisture retention capability.	Harsh and costly synthesis process; Specific surface area lower than that of ZIF‐MOFs; Limited adsorption capacity for specific gases; Limited environmental adaptability; Limited release speed control technology.	[118]
Al‐MIL‐53	High specific surface area and porosity; Good chemical stability; Efficient adsorption performance; Good reusability; Environmental friendliness.	Limited adsorption selectivity; Limited environmental adaptability; Lower catalytic activity.	[119]
UiO‐66	High specific surface area and porosity; Good chemical and thermal stability; Adjustable pore size and surface properties; Multi‐functionality; Reusability.	High cost and operational complexity; Limited environmental adaptability; Pore‐size limitation of adsorption efficiency; Slow adsorption kinetics.	[120]
UiO‐66‐NH_2_	High specific surface area and porosity; Good chemical and thermal stability; Adjustable pore size and surface properties; Multi‐functionality; Reusability	High cost and operational complexity; Limited environmental adaptability; Pore‐size limitation of adsorption efficiency.	[121]
HKUST‐1	High specific surface area and porosity; Good chemical and thermal stability; Adjustable pore size and surface properties.	High cost and operational complexity; Limited environmental adaptability; Pore‐size limitation of adsorption efficiency.	[122]
MOF‐74	High specific surface area and porosity; Good chemical and thermal stability; Aadjustable pore size and surface properties; Multi‐functionality; Reusability	High cost and operational complexity; Limited environmental adaptability; Pore‐size limitation of adsorption efficiency.	[123]
Ln‐MOFs	High quantum efficiency and long lifetime; Color modulation and optical switching functions; Good mechanical and optical properties; Versatility.	Photoluminescence modulation is difficult; Low transparency; Poor film formation.	[124]
MOF‐808	High specific surface area and porosity; Excellent catalytic performance; Good solvent stability; Environmental friendliness; Multi‐functionality.	High cost and operational complexity; Limited environmental adaptability.	[125]

However, despite these advantages, MOFs still face significant challenges in practical applications. Mass transport in MOFs is often diffusion‐limited due to micropore‐dominated structures, resulting in slow adsorption kinetics.^[^ ^119^, ^120^, ^121^ ^]^ Additionally, many MOFs suffer from poor structural stability, limiting their operational lifetime in harsh environments. The synthesis of high‐performance MOFs is frequently associated with high costs, which hinders scalability.^[^ ^122^, ^123^, ^124^, ^125^ ^]^ Moreover, certain synthesis strategies may pose environmental risks due to the use of heavy metals.^[^ ^126^, ^127^, ^128^, ^129^ ^]^ Addressing these issues will require continuous refinement of MOFs fabrication methods to balance performance, stability, cost, and environmental compatibility.

### 2.3 Synthetic Strategies of MOFs‐NC Composites

**Figure 6:**
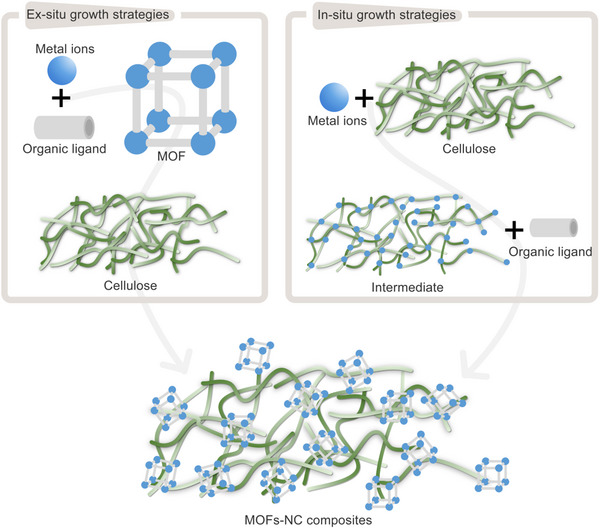
Schematic illustration of in situ growth and ex situ blending methods for the preparation of MOFs‐NC composites.

MOFs‐NC composites integrate the inherent properties of their individual components, while also exhibiting unique characteristics resulting from the synergistic interaction between MOFs and NC. Currently, there are two primary strategies for synthesizing MOFs‐NC composites by incorporating or introducing NC into MOFs (**Figure** **6**): one involves using NC as a template, introducing into the precursor solution prior to MOFs growth; while the other entails covalently or non‐covalently bonding (e.g., via hydrogen bonding or electrostatic interactions) MOFs with pre‐formed NC aggregates. Based on these strategies, the preparation methods for MOFs‐NC composites are typically classified into in situ and ex situ growth approaches. In situ growth strategies is commonly regarded as the preferred method, as it enables the formation of unique layered structures and addresses the issue of structural shrinkage frequently encountered in various MOFs‐NC composite forms. The resulting composites exhibit chemical stability and have found extensive applications in areas such as flame retardancy, thermal insulation, adsorption, degradation, electromagnetic shielding, energy storage, and sensing. In contrast, the ex situ growth method has successfully loaded MOFs, including ZIF‐8, ZIF‐67, HKUST‐1, UiO‐66, and MIL‐100, onto the NC matrix framework. This method can generate specialized microstructures, such as layered hierarchical porous structures, which enhance the functional properties of MOFs‐NC composites. These composites are increasingly used as novel materials in fields such as water treatment, energy storage, and green building applications. Furthermore, this study systematically collates various preparation methods, with a comprehensive comparative analysis of all involved preparation strategies provided in **Table** **4**.

**Table 4:** Comparison of different synthesis methods used for preparation of MOFs‐NC composites.

Synthesis methods	Advantages	Disadvantages	Improvement	Refs.
In situ growth method	Facile fabrication; MOF disperses evenly; Significant functional synergies; Strong interface bonding; Stable thermodynamics.	Uncontrollable reaction conditions; Insufficient performance repeatability; Difficulty in controlling MOF crystallinity.	Optimize the synthesis conditions; Interface engineering enhanced bonding; Precisely control the growth of MOF.	[52, 56]
ex situ growth strategies	Flexibility to choose composite strategies; High feasibility of large‐scale production; Tunable loading amount and morphology of MOF.	Laborious preparation steps; Uneven MOF loading; Limited composite performance; Low interfacial bonding strength; Unstable thermodynamic properties	Enhanced interface interactions; Improved dispersion uniformity Optimization of the composite process.	[131]
Self‐crosslinking method	Process simplicity and efficiency; Uniform composite; Structural stabilization; Environmentally friendly; Functional synergy.	Restricted reaction conditions; Difficult to control cross‐linking degree; Insufficient interfacial bond strength; Challenges of scaling up.	Optimization of cross‐linking mechanisms; Regulation of composite structures; Development of gentle and universal processes; Feature expansion.	[132]
Sol‐gel process	Homogeneous Composite; Structural Controllability; Excellent mechanical flexibility; Tunable MOF content and size; Mild reaction conditions; High design flexibility.	Long reaction time; Structural shrinkage and pore collapse; Difficulty in controlling MOF crystallinity; Insufficient interface integration.	Optimization of gelation and drying processes; Enhanced MOF crystallization and interfacial bonding; Development of low‐temperature compatible MOF system; Green processes and scaled production.	[133]
3D printing method	Customization of complex structures; High material utilization; Rapid Prototyping; Suitable for a variety of MOFs Enhanced mechanical properties.	Poor material compatibility; Post‐processing complexity; Insufficient interfacial bond strength; Print resolution and speed limitations.	Ink formulation optimization; Development of low‐temperature/green processes; Multi‐scale structural regulation; Scale and intelligent production.	[134]
Mechanical synthesis method	Process simplicity and efficiency; Strong interface bonding; Scale‐up potential; Wide material compatibility.	Risk of structural damage; Poor dispersion uniformity; Low reaction controllability; Limited functional synergies.	Optimization of process parameters; Surface modification to enhance bonding; Performance enhanced design; Intelligent and green upgrade.	[135]
Ultrasonication method	Highly efficient dispersion; Homogeneous compounding; Rapid response and energy efficiency; Eco‐friendly.	High sensitivity of ultrasound parameters; Scale‐up production challenges; Limited interfacial bond strength; Limited functional synergies.	Enhanced interfacial chemical bonding; Hierarchical structural design; Scale up equipment innovation; Multifunctional collaborative development.	[136]

#### 2.3.1 In Situ Growth Strategies

The in situ growth method involves incorporating NC as a matrix into the precursor solution (metal or binder) of MOFs. When the appropriate conditions for the reaction between the metal ion precursor and the organic ligand are met, MOF crystals begin to nucleate, grow, and precipitate on the NC matrix template. The MOFs‐NC composites are then obtained through washing and drying. During this process, NC acts as a micro‐reactor for MOFs growth. The numerous crystallization nucleation sites on the surface of NC provide a high MOFs loading capacity. Moreover, the presence of NC during the crystallization of the MOFs precursor accelerates crystal growth, leading to controlled morphology and reduced crystal size.

**Figure 7:**
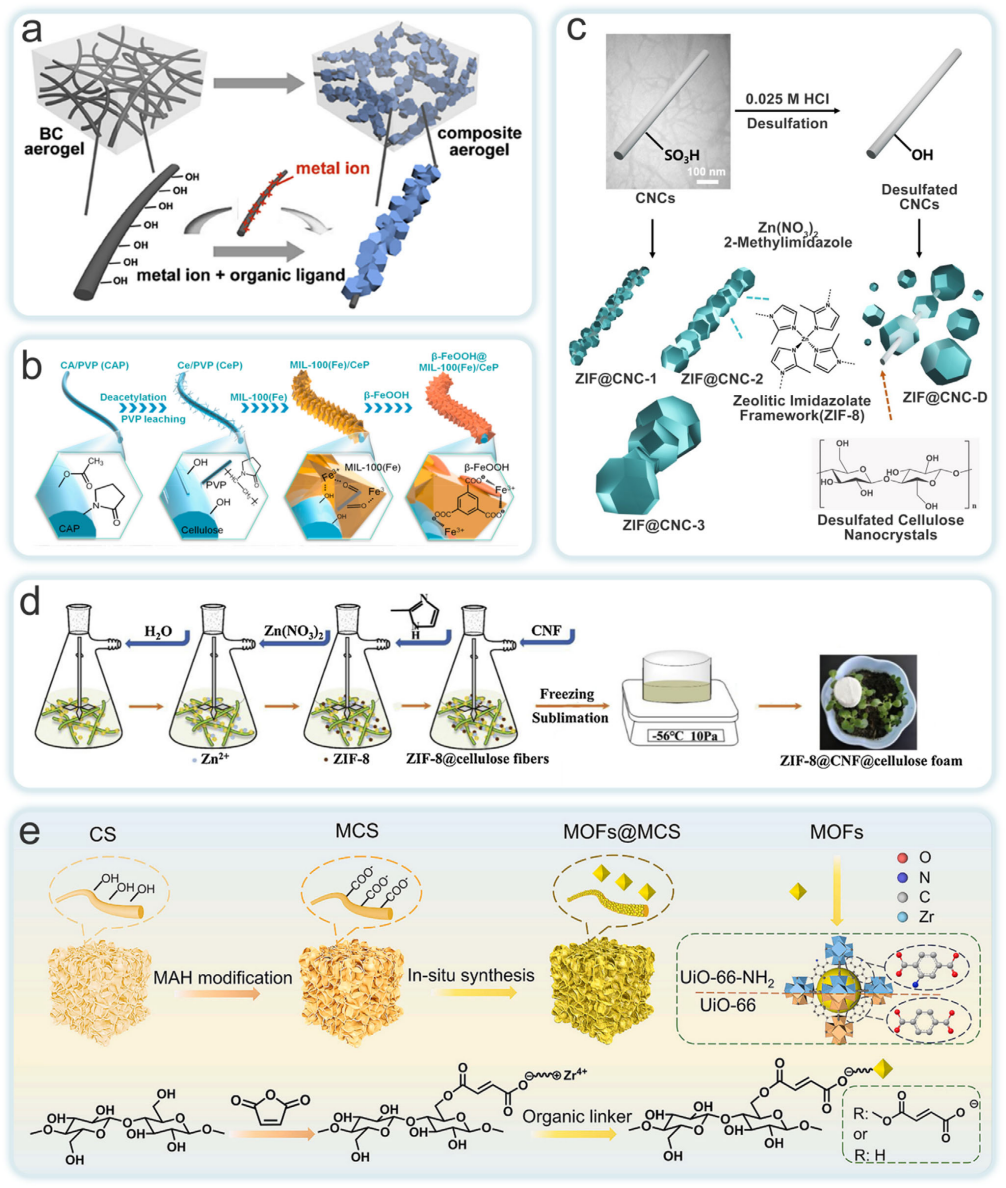
Schematic illustration of in situ growth for the preparation of CelloMOFs composites. a) BC@MOF. Reproduced with permission.^[^ ^52^ ^]^ Copyright 2019, Elsevier B.V. b) β‐FeOOH@MIL‐100(Fe)/CeP ENM. Reproduced with permission.^[^ ^56^ ^]^ Copyright 2021, Elsevier Ltd. c) Zn MOP@CNC. Reproduced with permission.^[^ ^130^ ^]^ Copyright 2023, Wiley‐VCH GmbH. d) ZIF‐8@CNF@cellulose foam. Reproduced with permission.^[^ ^126^ ^]^ Copyright 2019, Elsevier Ltd. e) MOFs@MCS. Reproduced with permission.^[^ ^59^ ^]^ Copyright 2024, Elsevier Ltd.

The inherent crystalline properties of MOFs have long hindered their practical applications in various fields. Conventional extrusion processing, for instance, is detrimental to preserving the porosity and catalytic activity of MOFs monomers. While typical methods for fabricating 3D MOFs structures can address some of these limitations, they still fail to provide adequate mechanical stability and MOFs yield. To overcome these challenges, Ma et al. investigated two MOFs materials, ZIF‐8 and UiO‐66 (**Figure** **7**a).^[^ ^52^ ^]^ Flexible BC aerogels were used as scaffold model, they introduced abundant reaction sites through the hydroxyl groups at the aerogel's ends. This approach not only facilitated the nucleation and growth of MOFs but also promoted the uniform distribution of MOFs particles on the BC. The resulting MOF aerogels contained numerous hydrogen bonds between BC nanofibers, enabling the material to withstand substantial strain. The composite aerogel not only preserved the high porosity of the MOFs but also retained the plasticity, flexibility, layered porosity, and low‐density characteristics of BC. This effectively addressed the longstanding challenge of processing MOFs into flexible, customizable structures. In a separate study, Lu et al. developed a green and efficient electrospun nanofiber membrane (ENM) for treating complex wastewater. They used electrospun deacetylated cellulose acetate (CA)/polyvinylpyrrolidone (CeP) nanofibers as the core structure, while in situ synthesizing a β‐hydroxy Fe oxide‐modified Fe‐based MOFs (β‐FeOOH@MIL‐100(Fe)) heterojunction as the photocatalytic shell to create a hybrid ENM (Figure 7b).^[^ ^56^ ^]^ The core‐shell structured ENM exhibited an exceptionally high MIL‐100(Fe) loading (78 wt%), a large surface area (1105 m^2^ g^−1^), and well‐dispersed β‐FeOOH nanorods. Due to the porous hydrophilic nature of MIL‐100(Fe) and the strong photocatalytic‐Fenton synergistic effect of β‐FeOOH@MIL‐100(Fe), the prepared ENM demonstrated outstanding performance, including high removal efficiencies for oil (99.5%), dye (99.4%), and chromium ions (99.7%), along with excellent cyclic stability.

While the in situ growth method is simple to operate and energy‐efficient, the amount of raw material can influence the uniformity of the MOFs loading. To address this issue, Cho et al. selected CNC as the matrix and controlled the formation of ZIF‐8 on the CNC surface by varying the ratio of ZIF‐8 precursor to CNC (Figure 7c).^[^ ^130^ ^]^ The researchers found that when the raw material was insufficient, ZIF‐8 could not fully cover the CNC surface, while an excess of material led to the binding of large individual ZIF‐8 crystals to each CNC particle. The average diameter of the formed ZIF‐8 crystals increased with the increasing ratio of raw materials. At the optimal ratio, ZIF‐8 nanocrystals were uniformly loaded on the surface of individual CNCs. When desulfurized CNC was used as the matrix at the same ratio, the lack of sulfate half‐ester groups hindered the anchoring of ZIF‐8 particles, resulting in incomplete loading on the CNC surface. The ZIF@CNC was used as a precursor to prepare MOP@CNC and Zn MOP@CNC, both of which demonstrated good performance and chemical stability in catalyzing the conversion of epichlorohydrin to carbonic acid vinyl chloride for CO_2_ fixation. Researchers successfully prepared various novel MOFs‐NC composites with functional properties using this method, and performance studies were conducted. Ma et al. employed a simple in situ green growth method to prepare a lightweight, porous ZIF‐8@CNF@cellulose foam (Figure 7d).^[^ ^126^ ^]^ Using pure water as the base and a green process without dimethylformamide (DMF), ZIF‐8 nanocrystals functionalized the cellulose fibers. Then, using CNF as a crosslinking agent, they prepared ZIF‐8@cellulose‐functionalized fiber composite foam materials by freeze‐casting. The presence of CNF effectively reduced or even prevented the shedding of ZIF, and exhibited excellent mechanical properties. The compressive strength of ZIF‐8/cellulose‐based foam containing 40 wt.% CNF reached 1.30 MPa. Yang et al. developed a general and effective strategy to load functional zirconium‐based MOF (Zr‐MOF) onto maleic anhydride dehydrogenated CNF sponge (MCS) (Figure 7e).^[^ ^59^ ^]^ Through the in situ growth method, they synthesized a porous sponge with outstanding mechanical properties, i.e., MOFs@MCS composites. The porous MCS contained abundant carboxyl groups and exhibited excellent mechanical performance, serving as the support for MOFs. The composites demonstrated superior reusability and structural stability. Furthermore, the carboxyl groups of MCS can chelate the central Zr^4+^ ions causing the assembled MOFs to firmly entangle together, providing additional adsorption capacity for effectively removing metal ions and organic dyes.

#### 2.3.2 Ex Situ Growth Strategies

**Figure 8:**
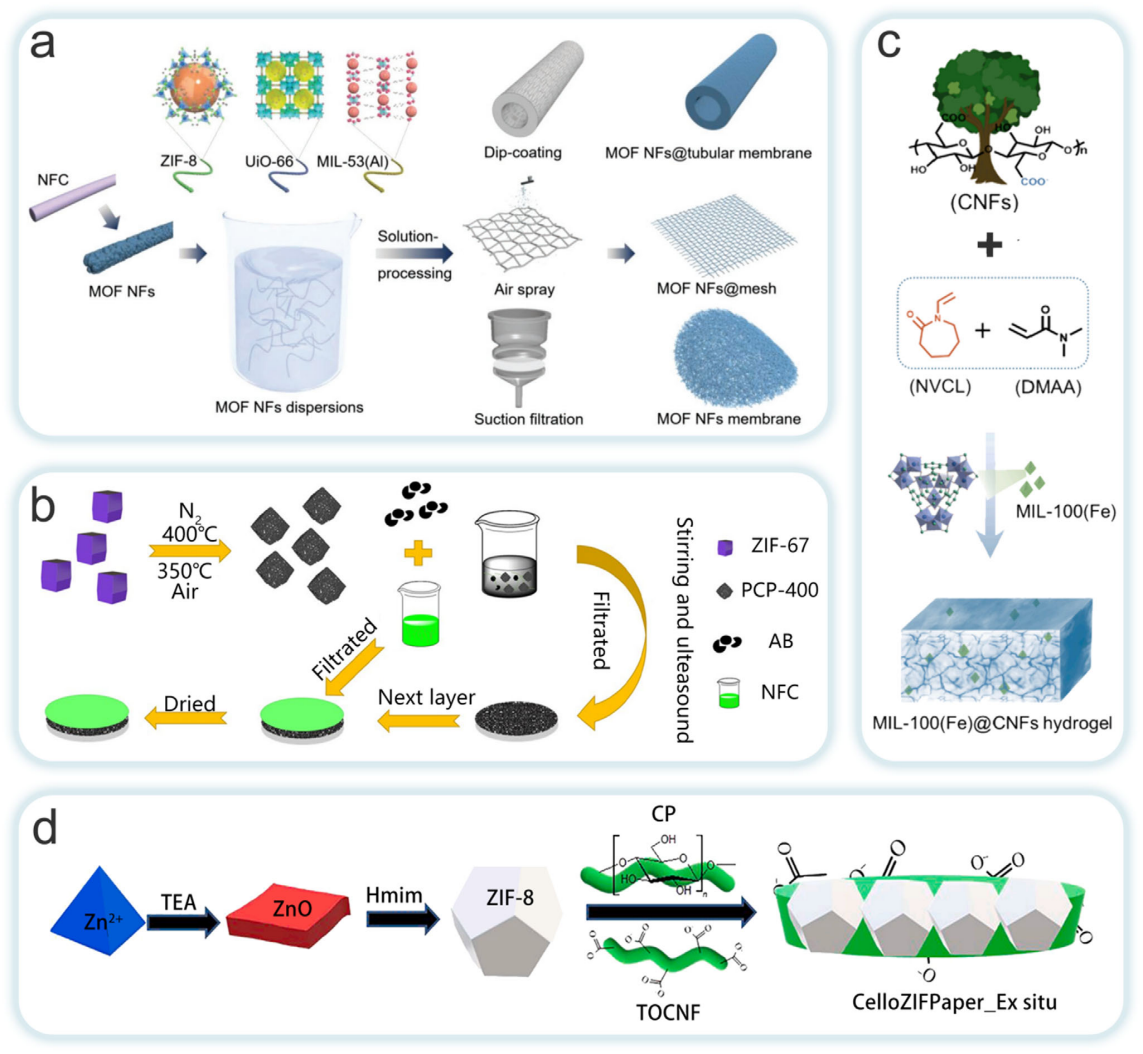
Schematic illustration of ex situ blending methods for the preparation of CelloMOFs composites. a) Blending MOF nanoparticles with nanofibrillated cellulose (NFC) through an ex situ approach to prepare NFC@MOF. Reproduced with permission.^[^ ^131^ ^]^ Copyright 2022, Wiley‐VCH GmbH. b) Nanocellulose/Porous Co_3_O_4_ Polyhedron (NPC). Reproduced with permission.^[^ ^131^ ^]^ Copyright 2021, Springer Nature Switzerland AG. c) MIL‐100(Fe)@CNFs hydrogel(MC). Reproduced with permission.^[^ ^131^ ^]^ Copyright 2021, Elsevier B.V. d) CelloZIFPaper. Reproduced with permission.^[^ ^131^ ^]^ Copyright 2022, Elsevier B.V.

The ex situ growth method involves the physical mixing of pre‐synthesized MOFs with NC in a solvent or solution. Both materials possess abundant active functional groups on their surfaces, allowing for interactions such as electrostatic forces, hydrogen bonding, and van der Waals forces between MOFs and NC. These interactions enhance their adhesion, resulting in a completed composite. Although this method is more complex and time‐consuming, requiring additional crosslinking agents for modification, which increases production costs. It offers the advantage of directly regulating the structure and properties of MOFs. This enables the targeted design and adjustment of MOFs‐NC composites. The synthesis process of this method involves the direct preparation of NC through mechanical fibrillation, followed by the fabrication of MOFs‐NC composites with excellent physicochemical properties using related techniques. Therefore, the ex situ growth strategy has significant potential in the preparation of MOFs‐NC composites in the future. Li et al. proposed a universal strategy by templating pre‐synthesized MOFs (such as ZIF‐8 and UiO‐66) onto tunicate‐derived NC, in combination with continuous‐flow synthesis, to fabricate high‐aspect‐ratio MOFs‐NC composites (**Figure** **8**a).^[^ ^131^ ^]^ This method preserves the crystalline structure and dispersibility of MOFs while enabling solution‐processable membrane fabrication, making it suitable for adsorption and catalytic applications. Building upon this, Xiao et al. employed a water‐based layer‐by‐layer papermaking approach to integrate MOF‐derived Co_3_O_4_ polyhedra with a NC scaffold (Figure 8b),^[^ ^131^ ^]^ yielding binder‐free, flexible, and freestanding electrode films. These composites exhibited excellent areal capacitance and mechanical flexibility, providing promising material platforms for next‐generation supercapacitors.

Meanwhile, Lin et al. introduced pre‐synthesized MIL‐100(Fe) particles into a NC matrix via free‐radical polymerization to construct a hydrogel that is simultaneously responsive to both temperature and pH (Figure 8c).^[^ ^131^ ^]^ The resulting hydrogel demonstrated outstanding environmental adaptability for controlled urea release. Abdelhamid et al. reported a method for producing hierarchically porous ZIF‐8 paper via Rapid–Köthen (R.K.) processing (Figure 8d).^[^ ^131^ ^]^ This method addressed issues related to synthesis, processing, and environmental friendliness, overcoming a significant barrier to the commercial application of MOFs.

Collectively, these studies demonstrate that ex situ MOFs‐NC composite strategies allow precise control over composition, structural tunability, and enhanced processability. This approach provides a versatile platform for developing functional materials that overcome the constraints of traditional in situ synthesis. However, partial MOFs‐NC composites such as CelloZIFPaper prepared through in situ synthesis, demonstrated superior performance compared to ex situ synthesis. This may be attributed to the fact that in the material prepared by ex situ synthesis, the MOF crystals and cellulose were loosely bound, leading to easier detachment of the crystals during practical applications, which in turn affected the material's stability and performance.

#### 2.3.3 Other Strategies

In addition to the two mainstream preparation strategies mentioned above, researchers are continually exploring alternative synthesis methods to further enhance the development of high‐performance MOFs‐NC composites.

**Figure 9:**
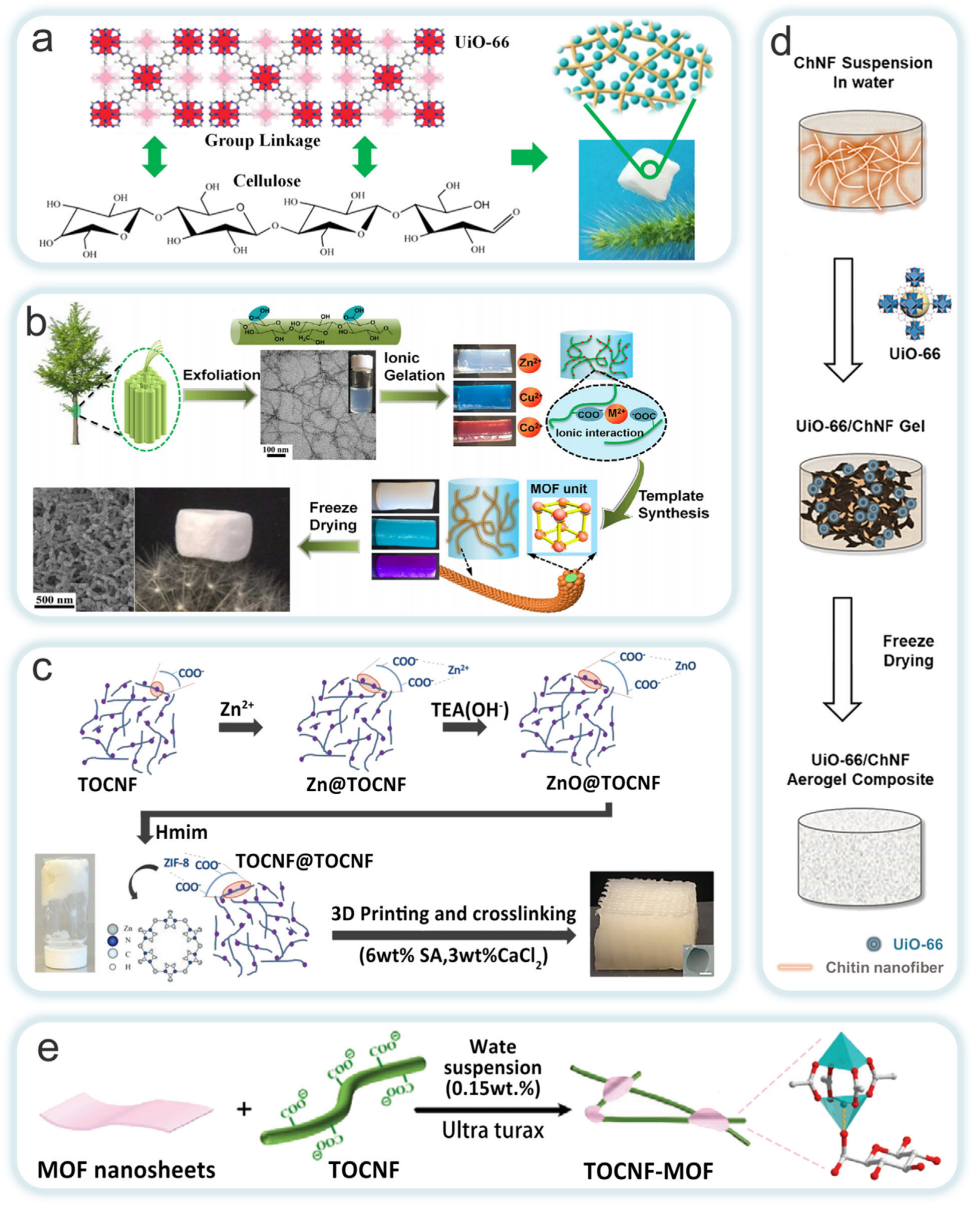
a) Construct UiO‐66/NC aerogels through a self‐crosslinking strategy. Reproduced with permission.^[^ ^132^ ^]^ Copyright 2019, Elsevier B.V. b) Sol‐gel process utilized to fabricate TEMPO‐CNFs@MOF aerogel. Reproduced with permission.^[^ ^133^ ^]^ Copyright 2018, ACS. c) Prepare CelloZIF8 by method which combines one‐pot synthesis and 3D printing technology. Reproduced with permission.^[^ ^134^ ^]^ Copyright 2018, WILEY‐VCH Verlag GmbH & Co. KGaA, Weinheim. d) Prepare ChNF/UiO‐66 through a combination of in situ chemical synthesis and mechanical strategies. Reproduced with permission.^[^ ^135^ ^]^ Copyright 2024, Elsevier. e) Fabrication AIEgen‐MOF/CNFs through combining ultrasonic synthesis and ex situ growth methods. Reproduced with permission.^[^ ^136^ ^]^ Copyright 2022, Wiley‐VCH GmbH.

Existing strategies are often constrained by the complexity of the manufacturing process, leading to challenges such as poor interfacial interactions and high production costs. To address these issues, Wang et al. employed a self‐crosslinking method, omitting chemical binders, to prepare a cellulose aerogel composite with hierarchical pores and high UiO‐66 loading (**Figure** **9**a).^[^ ^132^ ^]^ The oxygen‐containing groups (Zr‐OH) on UiO‐66 crosslink with the hydroxyl groups on the cellulose chains via hydrogen bonding, ensuring high dispersion of UiO‐66 particles while preserving micropores. This manufacturing strategy not only maintains the adsorption capacity of UiO‐66 but is also applicable to other oxygen‐containing group MOFs (e.g., ZIF‐8, MOF‐5, MIL‐100, etc.). Zhu et al. used TOCNF as a template to synthesize MOFs, achieving a high micropore/mesopore ratio in the MOF crystals while combining the layered porosity, flexibility, moldability, and low density of cellulose aerogels (Figure 9b).^[^ ^133^ ^]^ After mixing the CNF suspension with an M^2+^ (Zn^2+^, Cu^2+^, or Co^2+^) solution, the carboxyl groups on the TOCNF surface chelate with M^2+^, resulting in a CNFs‐M^2+^ hydrogel. The corresponding ligand solution was then added allowing the MOF crystals to grow. During this process, NC promoted crystal nucleation rather than growth, reducing the MOF crystal size and preventing particle aggregation, thus preserving the optimal performance of the MOFs particles. The results showed that the obtained aerogels exhibited high adsorption capacity and rapid adsorption rates for various molecules, along with high mechanical strength, demonstrating significant practical application potential. Sultan et al. introduced a novel MOFs processing method by combining TOCNF with ZIF‐8 and MIL‐100 (Fe) via 3D printing to fabricate multifunctional CelloMOF hydrogels, synthesizing and printing with water as a solvent at room temperature (Figure 9c).^[^ ^134^ ^]^ The mixed ink exhibited excellent shear‐thinning properties, extrudability, and shape fidelity post‐printing. During preparation, drugs such as curcumin were encapsulated into the CelloMOF system, and the subsequent release of guest molecules was controllable through pH, enabling medical applications. Moreover, the addition of curcumin enhanced the dispersion of ZnO nanoparticles within the cellulose fibers. This synthesis method broadens the potential applications of 3D printed CelloMOF in water and air purification, catalysis, sensors, and other fields, offering a novel approach for MOFs processing.

Seo et al. proposed a green synthesis strategy that combines both chemical and physical methods (Figure 9d).^[^ ^135^ ^]^ In this work, UiO‐66 was incorporated with chitosan nanofibers (ChNFs) and then freeze‐dried to create a highly stable, high surface‐area, porous ChNF/UiO‐66 composite aerogel. The strong interaction between ChNFs and UiO‐66 ensured stable loading of UiO‐66 onto the continuous nanoporous channels of the ChNF reaction membrane. The Brønsted basic functional groups on ChNFs facilitated the formation of robust interchain hydrogen bonds and metal–ammonia interactions with UiO‐66, ensuring that the catalyst remained stably incorporated into the reaction membrane even at higher UiO‐66 content. This interaction enhanced both the catalytic activity and recyclability of UiO‐66. In a related study, Tan et al. synthesized fluorescent composites with humidity‐sensing and UV‐shielding functions by combining aggregation‐induced emission (AIEgen)‐based MOF with CNFs (Figure 9e).^[^ ^136^ ^]^ A uniform suspension of MOF crystals was mixed with a mechanically homogenized TOCNF aqueous solution, and transparent films were then synthesized via solution casting. The MOF nanosheets increased the adhesion between CNFs, thereby enhancing the tensile strength and modulus of the composite films, and improving the mechanical properties. Additionally, the incorporation of MOF nanosheets improved UV light absorption and scattering. The open metal sites on the MOF interacted with carboxyl groups on the CNF surface, reducing π‐π stacking and effectively suppressing the non‐radiative motion of TPE phenyl rotors. This enhanced both the fluorescence and UV‐shielding performance of the composites, thereby expanding potential applications across a range of fields.

## 3 Forms of MOFs‐NC Composites

**Figure 10:**
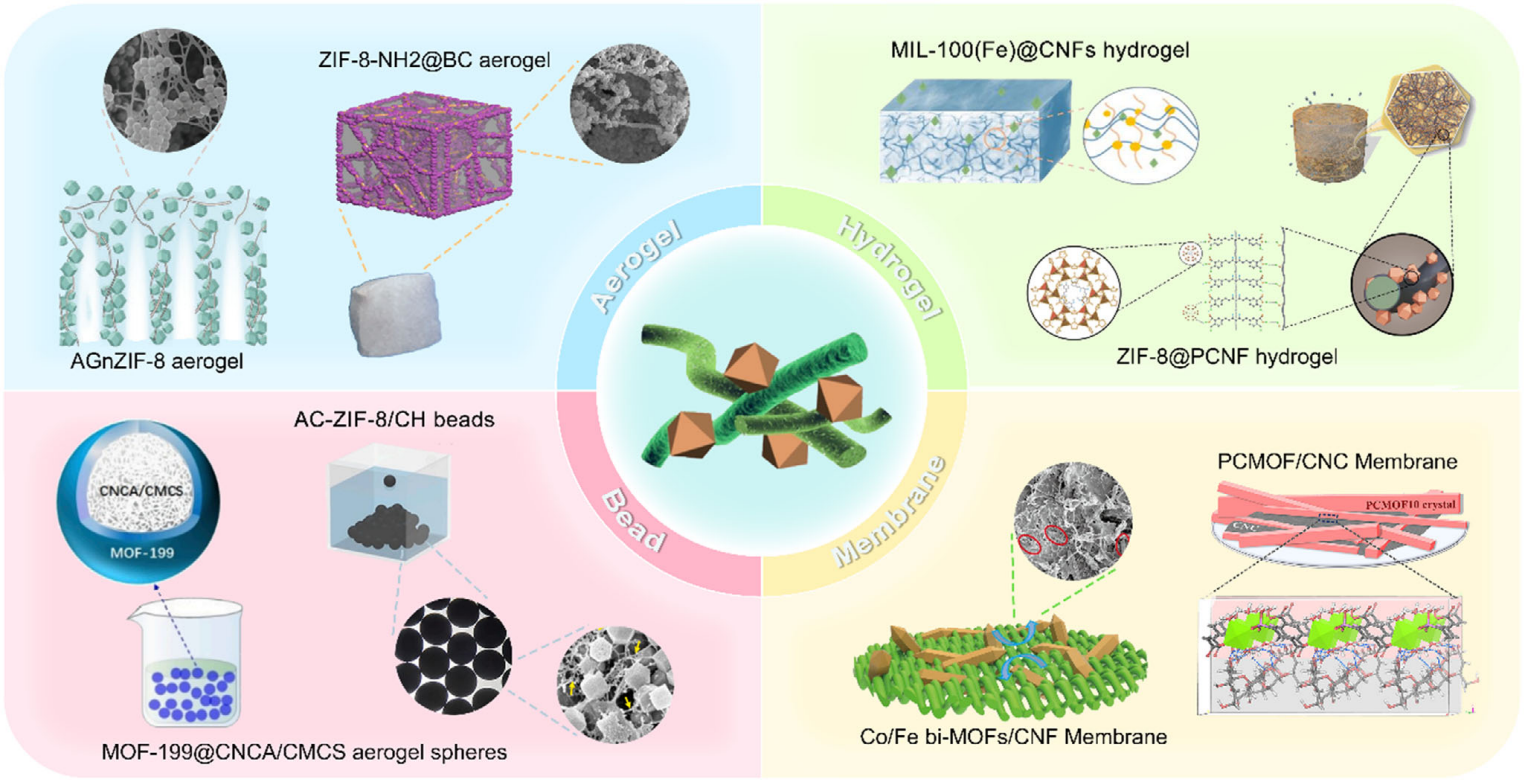
Comparisons of various forms of MOFs‐NC composites.

MOFs‐NC composites exhibit a range of distinct morphologies by various preparation strategies, providing a solid foundation for their application in environmental remediation.^[^ ^137^ ^]^ Common forms of MOFs‐NC composites include aerogels,^[^ ^66^ ^]^ hydrogels,^[^ ^67^ ^]^ membranes,^[^ ^68^ ^]^ beads,^[^ ^69^ ^]^ and others (**Figure** **10**). These morphological variations not only significantly influence the performance mechanisms of MOFs‐NC composites but also substantially alter their functionality in real‐world applications. By combining and optimizing different forms, these materials can offer more efficient and sustainable solutions to specific environmental challenges. This section will discuss the preparation strategies and microstructures of various forms of MOFs‐NC composites, as well as their performance response mechanisms in the context of environmental remediation.

### 3.1 Aerogel

**Figure 11:**
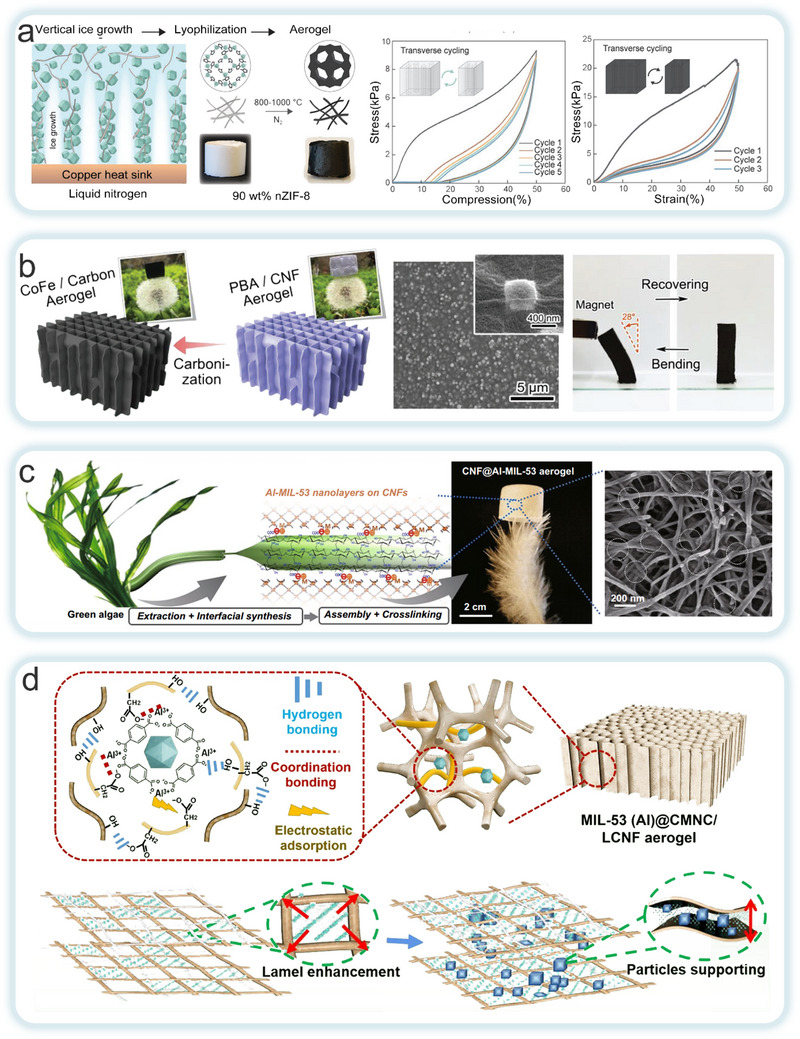
a) The formation of MOAs and CAGs by freeze‐drying and carbonization in turn and structural schematics, and compressive stress‐strain curves for AGnZIF‐8. Reproduced with permission.^[^ ^139^ ^]^ Copyright 2022, Wiley‐VCH GmbH. b) Structural diagram, SEM images and bending performance of CoFe/Carbon aerogel. Reproduced with permission.^[^ ^140^ ^]^ Copyright 2024, Wiley‐VCH GmbH. c) CNF@AI‐MIL‐53 ultralight aerogel prepared from CNF extracted from algae, and SEM images. Reproduced with permission.^[^ ^141^ ^]^ Copyright 2020, Springer Nature. d) Structural grading diagram of MIL‐53 (Al)@CMNC/LCNF aerogel and schematic illustration of enhancement mechanism of mechanical strength in M@CLA by MIL‐53(Al) nanoparticles. Reproduced with permission.^[^ ^142^ ^]^ Copyright 2024, Springer Nature Switzerland AG.

MOFs‐NC aerogels are materials characterized by extremely low density and high specific surface area, which are primarily fabricated via freeze‐drying. A homogeneously dispersed suspension of MOFs and CNF or CNC is first frozen, followed by the sublimation of ice crystals to remove water, resulting in a 3D network with a hierarchical porous structure. This process requires precise control of the freezing rate and solid content to balance porosity and mechanical strength, thereby avoiding collapse of the pore channels caused by NC network shrinkage.^[^ ^70^ ^]^ Benefiting from the templating effect of NC and the intrinsic microporosity of MOFs, the resulting MOFs‐NC aerogels exhibit high specific surface area and synergistic adsorption performance. Moreover, the thermal stability of NC and its encapsulation of MOF particles contribute to the structural integrity of the composites under high‐temperature conditions. However, to maintain stability in long‐term aqueous environments, crosslinking modification is necessary. Surface modifications, either chemical or physical, such as the introduction of specific functional groups or metal ions, can further enhance their capacity to target and remove specific pollutants.^[^ ^138^ ^]^ Rostami et al. developed a multifunctional aerogel (MOAs) with up to 90% MOF particle content, as well as derived carbon aerogel (CAGs) through a simplified aqueous synthesis and freeze‐casting process (**Figure** **11**a).^[^ ^139^ ^]^ The initial adsorption of MOFs at the CNF/water interface was governed by van der Waals forces. While electrostatic repulsion between like charges prevents macroscopic flocculation, thereby preserving the inherent properties of both materials. The high aspect ratio of CNF results in the formation of a highly entangled gel network at low concentrations, which transformed into a fine and robust grid structure upon dehydration. The high specific surface area and volume ratio of MOF facilitated their uniform dispersion within the CNF matrix, thereby significantly enhancing the mechanical properties and durability of the aerogel. Additionally, Qiao et al. developed a bioinspired, lightweight, elastic, multifunctional porous magnetic carbon aerogel using the in situ growth strategy of prussian blue analogs (PBA) on CNF (Figure 11b).^[^ ^140^ ^]^ Magnetic nanocapsules derived from PBA exhibited excellent dispersion and interface bonding. And when embedded within the carbon framework, they markedly enhanced the material's mechanical and electromagnetic properties. The PBA/CNF hybrid material aligned between ice crystals by utilizing a unidirectional ice template method, excluding from the freezing front. This arrangement forms oriented pore walls between the ice crystals, creating an anisotropic honeycomb structure that further improved the material's flexibility and toughness. This structural design enabled the material to excel in multifunctional applications such as thermal insulation, flame retardancy, and electromagnetic shielding. This work offered new directions for the development of high‐performance aerogels. On the other hand, Zhou et al. employed a stepwise assembly method to grow Al‐MIL‐53 nanosheets in situ on CNF surfaces, creating a hybrid CNF@MOF aerogel with a unique core‐shell nanostructure (Figure 11c).^[^ ^141^ ^]^ This structure further extended into a cross‐linked nanofiber network, imparting the aerogel with exceptional mechanical strength and superelasticity. The honeycomb network structure and hierarchical pore distribution of the CNF@MOF aerogel effectively prevent inward heat transfer. Li et al. employed a carboxymethylated NC (CMNC) bridging technique to design an MOF@cellulose nanofiber aerogel (Figure 11d).^[^ ^142^ ^]^ Combining CMNC with MIL‐53 (Al) nanoparticles significantly increased pore structure complexity and inhibited intramolecular hydrogen bonding between fibers, thereby improving the stability of the fiber network. The strong molecular interactions between MIL‐53 (Al) and the fibers not only improved the aerogel's mechanical strength but also significantly enhanced the flame‐retardant properties.

### 3.2 Hydrogel

MOFs‐NC hydrogels are prepared using methods such as in situ growth, physical mixing, post‐treatment modification, and chemical or physical crosslinking. The MOFs‐NC hydrogels offer greater functional diversity compared to traditional hydrogels.^[^ ^143^ ^]^ The formation of hydrogels is based on the synergistic effect of physical and chemical crosslinking. By mixing MOFs precursors with a NC solution, the hydrogen‐bonded network of NC forms a physical scaffold. Simultaneously, coordination interactions occur between the metal nodes of MOFs and the oxygen‐containing functional groups of NC, establishing chemical crosslinking. This dual mechanism ultimately leads to the construction of composite hydrogels with a 3D porous architecture. Owing to the structural tunability of MOFs and the biocompatibility of NC, MOFs‐NC hydrogels exhibit strong environmental responsiveness, enabling smart reactions to external stimuli such as pH, temperature, and light. However, during actual synthesis, excessive MOFs loading may cause material embrittlement due to the aggregation of MOFs particles, which disrupts the NC network. Furthermore, in hydrophobic MOFs systems, surface modification of NC is required to enhance interfacial compatibility and maintain a balance between porosity and hydrophilicity.

**Figure 12:**
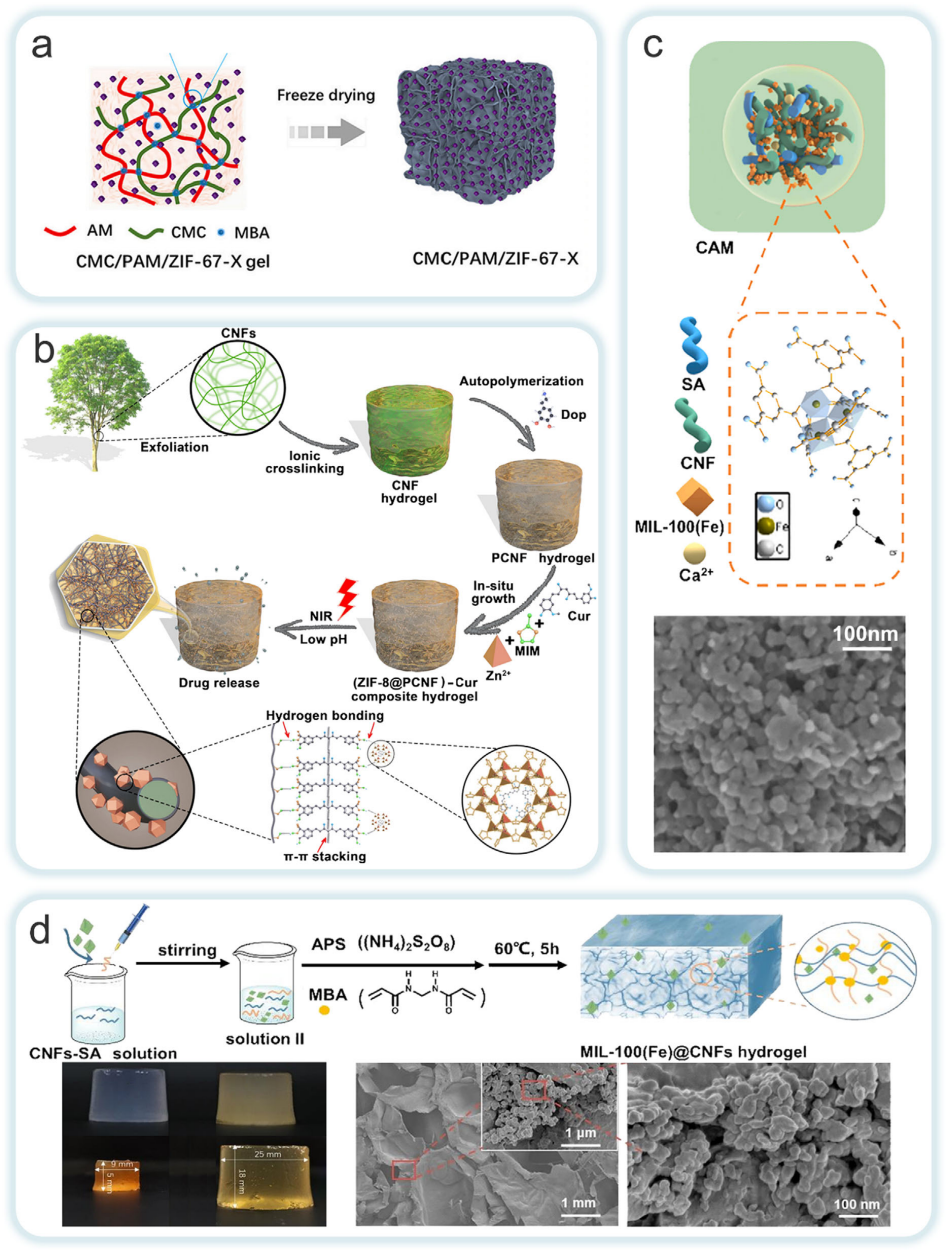
a) The synthesis line of CMC/PAM/ZIF‐67‐X. Reproduced with permission.^[^ ^144^ ^]^ Copyright 2024, Elsevier B.V. b) The schematic plot of the preparation of ZIF‐8@PCNF hydrogel and schematic diagram of hierarchical structure. Reproduced with permission.^[^ ^145^ ^]^ Copyright 2021, Springer Nature B.V. c) The scheme of the preparation of the U‐CAM and SEM image of CAM. Reproduced with permission.^[^ ^118^ ^]^ Copyright 2021, Elsevier B.V. d) Preparation, macroscopic morphology, SEM image of MIL‐100(Fe)@CNFs hydrogel. Reproduced with permission.^[^ ^131^ ^]^ Copyright 2024, Springer Nature Switzerland AG.

For instance, Yang et al. employed a network repair strategy to incorporate ZIF‐67 into a carboxymethyl cellulose/polyacrylamide (CMC/PAM) matrix, thereby preparing a composite hydrogel (**Figure** **12**a).^[^ ^144^ ^]^ The carboxyl and amino groups on the surface of the CMC/PAM matrix promoted the uniform growth of ZIF‐67 through coordination bonds, resulting in a directed and penetrating microchannel array. This approach effectively mitigated the aggregation problem of ZIF‐67 and improved the interface compatibility between the MOFs and the polymer, enhancing the composites's uranium adsorption performance from seawater. Dynamic coordination repair further enhanced the mechanical properties and durability of the hydrogel after the incorporation of ZIF‐67, enabling to exhibit higher stability and adsorption efficiency in turbulent seawater environments. Liu et al. prepared a composite hydrogel with improved mechanical properties by in situ growing ZIF‐8 on the surface of polycrystalline nanofiber (PCNF)‐coated poly(3,4‐dihydroxyphenylalanine) (PDA) hydrogels (Figure 12b).^[^ ^145^ ^]^ The Zn^2^⁺ ions and the PCNF matrix significantly increased the physical strength of the composite hydrogel through chelation, π‐π interactions, van der Waals forces, and hydrogen bond synergistic effects. In a separate study, Wang et al. designed a pH‐sensitive urea‐controlled release fertilizer (U‐CAM) by incorporating cellulose‐based hydrogels with MIL‐100(Fe) MOF (Figure 12c).^[^ ^118^ ^]^ Similarly, Lin et al. employed a free‐radical polymerization strategy to combine CNFs with MIL‐100(Fe), resulting in the preparation of a MIL‐100(Fe)@CNFs hydrogel (MC) (Figure 12d).^[^ ^131^ ^]^ The results demonstrated that the hydrophilic groups in cellulose‐based hydrogels contribute significantly to pH‐sensitive properties. The introduction of the MIL‐100(Fe) composite optimized the hydrogel's porous structure while reducing the hydrogen bonding interactions between the hydrophilic groups. Consequently, the degree of physical entanglement in the hydrogel decreased, which slowed the rapid release of urea and enhanced the stability and efficiency of the controlled‐release fertilizer.

### 3.3 Beads

MOFs‐NC beads are promising candidates for high‐performance adsorption composites due to their excellent hydrophilicity, plasticity, high porosity, and large specific surface area. The core preparation strategy focuses on achieving particle size uniformity through emulsion templating and spray drying techniques, thereby meeting the requirements for continuous industrial‐scale operations. The emulsion templating method utilizes reverse‐phase emulsion polymerization to encapsulate MOFs‐NC material into microspheres. This approach is capable of incorporating sensitive biomolecules; however, it necessitates the removal of organic solvents in subsequent processing. In contrast, spray drying directly converts MOFs‐NC suspensions into microspheres via atomization and rapid drying. Although this method is highly efficient, the elevated temperature during drying may compromise the structural integrity of MOFs. Compared to traditional beads, MOFs‐NC beads can achieve tailored functionalities by selecting different types of MOFs or modifying the NC surface. In addition, due to their precisely tunable particle sizes in the micron‐to nanometer‐scale range and high mechanical strength, such bead‐like materials exhibit excellent applicability in industrial settings such as packed beds or fluidized beds.

**Figure 13:**
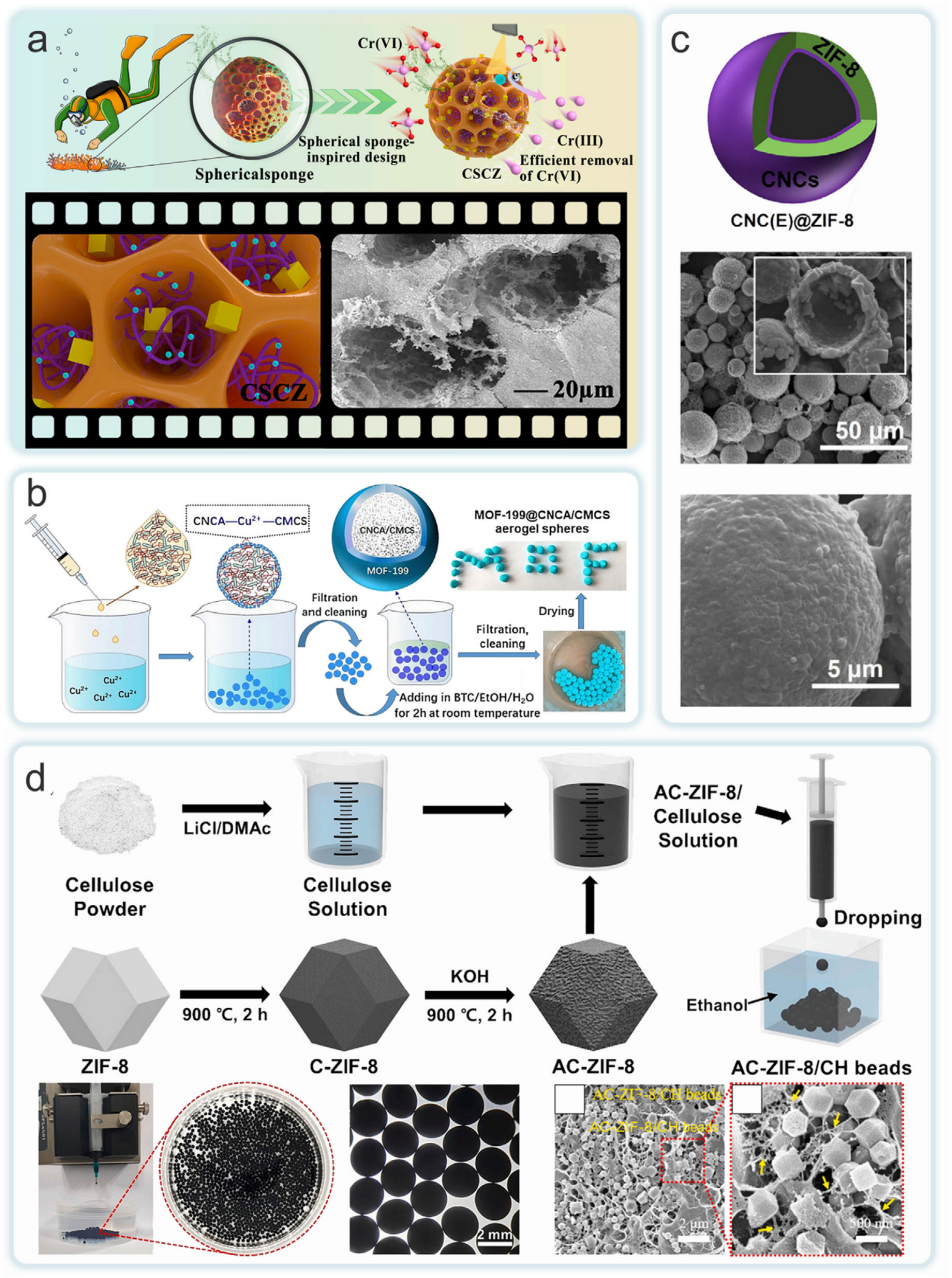
a) Spherical sponge‐inspired strategy, morphological features of main components and SEM image of CSCZ. Reproduced with permission.^[^ ^146^ ^]^ Copyright 2024, Elsevier B.V. b) Schematic illustration of the synthetic process of MOF‐199@CNCA/CMCS aerogel sphere. Reproduced with permission.^[^ ^147^ ^]^ Copyright 2022, Elsevier B.V. c) Structural schematic diagram and SEM images of CNCs@ZIF‐8 hollow microspheres. Reproduced with permission.^[^ ^148^ ^]^ Copyright 202, Elsevier Inc. d) Schematic diagram and photographic images of AC‐ZIF‐8/CH bead formation process. Optical microscopic images and FE‐SEM images of AC‐ZIF‐8/CH beads. Reproduced with permission.^[^ ^149^ ^]^ Copyright 2024, Elsevier.

Zhang et al. developed chitosan/silver cluster‐loaded CNF/Cu‐ZIF‐8 gel beads (CSCZ), inspired by spherical sponges (**Figure** **13**a).^[^ ^146^ ^]^ The porous channels of the chitosan matrix serve to protect the internal active components. The cellulose nanofibrils possessed a high surface area and contained nitrogen‐functional groups, both of which facilitate amino‐induced Cr^6^⁺ adsorption. Furthermore, UV light induces Cu‐ZIF‐8 to generate electron‐hole pairs, thereby promoting the reduction of Cr^6^⁺ and significantly enhancing the removal efficiency of heavy metal ions. Zhang et al. employed a combination of ion crosslinking and coordination bonding to in situ grow MOF‐199 crystals on the surface of carboxylated cellulose nanocrystals (CNCA) and carboxymethyl chitosan (CMCS) templates, thereby preparing core‐shell aerogel (Figure 13b).^[^ ^147^ ^]^ The dense MOF‐199 crystal shell and mesoporous structure effectively increased the adsorption capacity for methylene blue (MB) through the negative charge effect of BTC ligands, which efficiently bind to the target molecules. Ma et al. employed a pickering emulsion‐induced in situ growth method to prepare composite hollow microspheres based on CNC and ZIF‐8 (Figure 13c).^[^ ^148^ ^]^ The dense ZIF‐8 shell and the hydrophilic CNC layer worked synergistically, demonstrating excellent performance in the removal of MB. Lee et al. utilized cellulose dissolution‐regeneration technology to incorporate mesoporous activated carbon (AC) and ZIF‐8 powder into a cellulose matrix, thus creating microsphere composites for efficient adsorption of rhodamine B (RhB) (Figure 13d).^[^ ^149^ ^]^ The hydrophilic cellulose matrix facilitated the penetration of dye molecules, while the micro/mesoporous structure of AC‐ZIF‐8 along with multiple interactions with the dye molecules further enhanced the adsorption capacity.

### 3.4 Membrane

Compared to other materials, the feature more regular pore structures and greater mechanical strength, enabling selective screening of substances based on pore size and chemical properties.^[^ ^150^ ^]^ The principal fabrication techniques for MOFs‐NC composite membranes include in situ growth, solution blending, layer‐by‐layer self‐assembly, and interfacial synthesis. Among these, the blending method is the most widely adopted due to its operational simplicity and scalability. This method involves homogeneously mixing MOF particles with NC suspensions, followed by membrane formation through vacuum filtration or casting. It not only offers a straightforward process but also allows flexible tuning of component ratios while leveraging the reinforcing effect of NC. Key considerations during fabrication include the uniform dispersion of MOFs, precise control of NC concentration, optimization of drying conditions, and enhancement of interfacial adhesion. The synergistic integration of MOFs and NC yields membranes with hierarchical pore structures, high mechanical strength, abundant surface functional sites, and excellent environmental compatibility, making them promising for water purification and gas separation.

**Figure 14:**
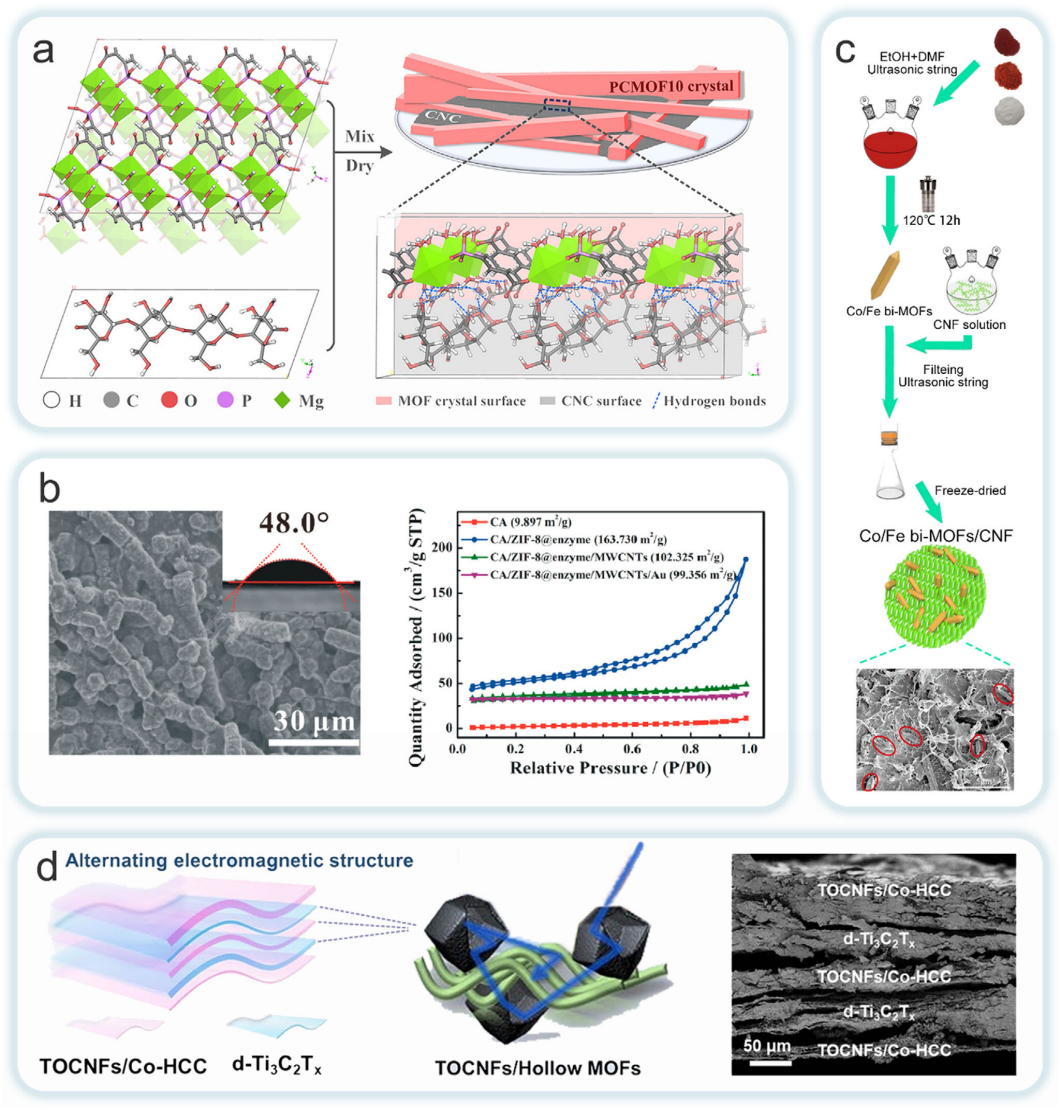
a) Structure and schematic diagram of the assembly of PCMOF10 and CNCs. Reproduced with permission.^[^ ^151^ ^]^ Copyright 2023, Elsevier Inc. b) FE‐SEM images, static water contact angle, and BET analysis of CA/ZIF‐8@enzyme/MWCNTs/Au membrane. Reproduced with permission.^[^ ^152^ ^]^ Copyright 2020, Elsevier B.V. c) Schematic illustration of the preparation process of Co/Fe bi‐MOFs/CNF membrane, and the SEM images. Reproduced with permission.^[^ ^153^ ^]^ Copyright 2020, Elsevier. d) Alternating electromagnetic structure, and SEM image of HMN membrane. Reproduced with permission.^[^ ^154^ ^]^ Copyright 2024, Springer Nature Switzerland AG.

Lin et al. reported a proton‐conducting MOF (PCMOF)/CNC membrane with a structurally and chemically stable composition, achieving exceptionally high MOF loading (**Figure** **14**a).^[^ ^151^ ^]^ The abundant intermolecular hydrogen bonds between PCMOF and CNC provided sufficient stability for fabricating mechanically, thermally, and hydrolytically stable membranes. However, despite the strong hydrogen bonds promoting adhesion between CNC and MOF crystals, more dynamic hydrogen bonding was required to facilitate proton conduction. Consequently, the proton conductivity of the PCMOF/CNC membrane did not exceed that of the pure MOF samples. Li et al. in situ grew ZIF‐8 on electrospun cellulose acetate (CA) nanofiber membranes to create a highly hydrophilic conductive electrode/ZIF‐8@enzyme membrane (Figure 14b).^[^ ^152^ ^]^ ZIF‐8 crystals were encapsulated within CA nanofibers, forming a unique beaded structure. The protective layer formed by ZIF‐8 on the surface of CA nanofibers effectively prevents rapid heat transfer. The porous structure of ZIF‐8 not only provided a high specific surface area but also absorbs and disperses heat reducing localized overheating. Furthermore, the incorporation of ZIF‐8@enzyme, MWCNTs, and AuNPs promoted hydrogen bond formation between cellulose chains, enhancing interaction and increasing the material's rigidity and thermal stability. However, the addition of ZIF‐8 increased the breaking stress of CA, while the water‐swelling characteristics of cellulose reduced mechanical strength. Additionally, the crosslinked structure of cellulose fibers was easily disrupted under the same temperature and pressure. Hou et al. synthesized rod‐shaped Co/Fe dual MOFs using a simple one‐step solvothermal method and loaded onto CNFs via vacuum filtration (Figure 14c).^[^ ^153^ ^]^ The vacuum‐filtered CNFs formed high‐density hydrogen bonds and physical entanglements due to the high aspect ratio and fiber interactions of CNFs, maintaining original shape even after multiple catalytic experiments. Mai et al. employed an in situ epitaxial growth method to prepare core‐shell ZIF‐8@ZIF‐67 structures, with ZIF‐8 as the core and Co^2^⁺ attracted to the surface through electrostatic interactions (Figure 14d).^[^ ^154^ ^]^ Organic ligand C_4_H_6_N_2_ was then added to promote the growth of ZIF‐67 on the surface. During the subsequent pyrolysis process, the metal nodes in ZIF‐8 gradually evaporated from the “core center” at high temperatures, forming MOF‐derived cobalt hollow carbon cages (Co‐HCC). Using an alternating vacuum‐assisted filtration (AVAF) method, a stable HMN composite membrane was formed. The results showed that the HMN membrane had a distinct gradient alternating structure, which helped leverage the individual advantages of the different components in the material. Additionally, the tight connections between layers resulted in slight infiltration, further stabilizing the membrane structure.

## 4 Application of MOFs‐NC Composites in Environmental Remediation

MOFs‐NC composites have attracted considerable attention in recent years for environmental remediation, owing to unique structural features and exceptional performance. These composites demonstrate remarkable efficacy in applications such as dye removal, pharmaceutical pollution control, heavy metal removal, VOCs degradation, greenhouse gas mitigation, and PM clearance. MOFs, with extremely high surface area, tunable pore structures, and functionalized surfaces, show substantial promise for environmental remediation. However, the low stability and limited regenerability of MOFs pose significant challenges for practical applications. The incorporation of NC enhances the stability and regenerability of MOFs, while also improving the economic viability of the MOFs‐NC composites. Due to the biodegradability of NC and the tunable pore structure of MOFs, the MOFs‐NC composites can be easily regenerated through physical methods (e.g., pyrolysis, UV radiation) or chemical methods (e.g., solvent washing, acid‐base treatment). This characteristic reduces the long‐term costs of environmental remediation and enables large‐scale, sustainable environmental restoration. By combining the strengths of both materials, MOFs‐NC composites markedly improve their effectiveness in environmental remediation. The application potential of MOFs‐NC composites in environmental remediation is expected to expand with ongoing advancements in preparation technologies, offering increasingly sustainable solutions for global pollution control.

### 4.1 Dye Adsorption and Degradation

Dye wastewater is characterized by high concentrations, deep chromaticity, complex chemical structures, and difficult biodegradation, posing a significant threat to the environment and ecosystem.^[^ ^155^ ^]^ The high specific surface area and rich porosity of MOFs‐NC composites provide abundant adsorption sites for dye molecules, enabling superior dye removal performance. Surface functional groups such as hydroxyl and carboxyl form chemical bonds with dye molecules, while π‐π stacking interactions between aromatic structures further enhance adsorption capacity. In photocatalytic degradation, MOFs‐NC composites can generate photogenerated electrons and holes under light irradiation, leading to the formation of highly •OH and O_2_⁻, which oxidize dye molecules. Furthermore, MOFs metal centers can catalyze the activation of persulfates to produce sulfate and •OH, facilitating dye degradation.^[^ ^156^ ^]^ Furthermore, the combination of MOFs and the hydrophilic carrier NC overcomes the limitations of powdered MOFs, such as poor dispersibility and recovery,^[^ ^157^ ^]^ significantly expanding application potential, particularly in the adsorption of cationic, anionic, and non‐polar dyes.^[^ ^158^ ^]^

**Figure 15:**
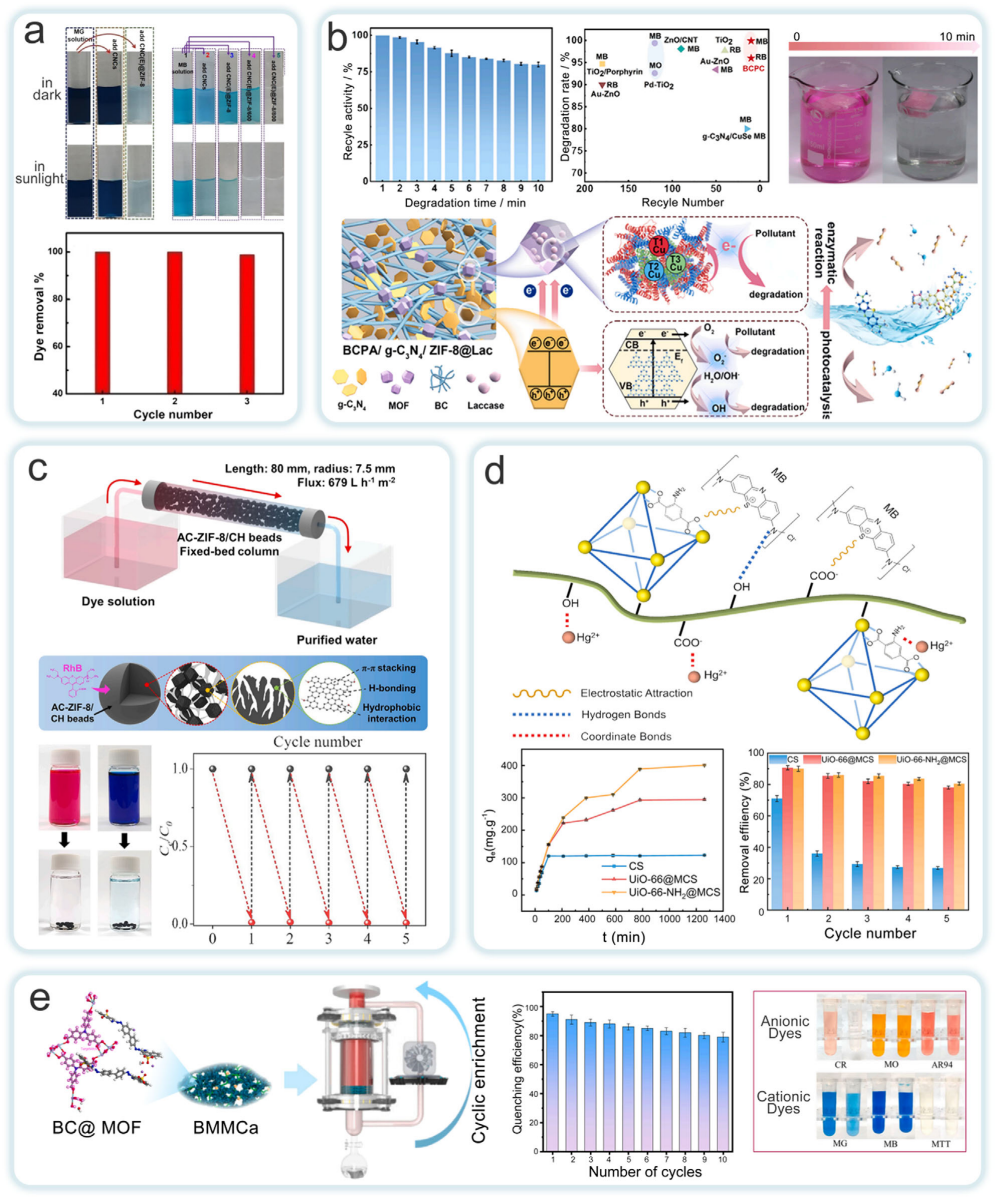
a) The degradation effect of CNCs/ZIF‐8 composite microspheres on MG, MB in dark and sunlight, and the cycling performance. Reproduced with permission.^[^ ^148^ ^]^ Copyright 2020, Elsevier Inc. b) Catalytic and recycle performance, degradation efficiency, and catalytic degradation mechanism of BCPC for dye molecules in wastewater. Reproduced with permission.^[^ ^58^ ^]^ Copyright 2024, Elsevier Ltd. c) Schematic illustration of continuous adsorption of RhB through AC‐ZIF‐8 bed column, the adsorption mechanisms, and cyclicity. Reproduced with permission.^[^ ^149^ ^]^ Copyright 2024, Elsevier Ltd. d) Molecular structure and application schematic diagram, adsorption efficiency and cycle performance of UiO‐66‐NH_2_@MCS composites. Reproduced with permission.^[^ ^59^ ^]^ Copyright 2024, Elsevier. e) DFT calculations of BC@MOF. Schematic diagram of the application facilities of BMMCa membrane and cycling performance. The adsorption effect of BMMCa on different dyes. Reproduced with permission.^[^ ^164^ ^]^ Copyright 2024, Elsevier B.V.

These strategies have been demonstrated to improve the adsorption efficiency and selectivity of MOFs‐NC composites to dye molecules in aqueous solution, assummarized in **Table** **5**. Ma et al. employed the pickering emulsion template method to in situ synthesize hollow CNCs/ZIF‐8 microspheres, which significantly enhanced the material's recyclability for the removal of malachite green (MG) and MB (**Figure** **15**a).^[^ ^148^ ^]^ The material boasted a specific surface area of 1240 m^2^ g^−1^ and exhibited remarkable hydrophilicity, with an adsorption capacity of 1060.2 mg g^−1^ for MG. The CNCs/ZIF‐8 shell consisted of dense ZIF‐8 crystals, and both the inner and outer surfaces were uniformly coated with a hydrophilic CNCs layer. This hydrophilicity strengthened the interaction between the material and dye molecules in the aqueous phase,^[^ ^159^ ^]^ while the porous ZIF‐8 network provided abundant adsorption sites for the dye molecules.^[^ ^160^ ^]^ Based on the 3D network structure of BC, Yu et al. integrated g‐C_3_N_4_ nanosheets with MOF‐loaded laccase (Lac) through self‐assembly to construct a photoenzyme‐coupled catalytic system (Figure 15b).^[^ ^58^ ^]^ The BC's porous network not only increased the loading capacity of the photocatalyst^[^ ^161^ ^]^ and enzymed but also effectively protected the enzyme from radical‐induced damage. The visible light responsiveness of g‐C_3_N_4_ combined with the protective encapsulation of laccase by the MOF synergistically enhances the transfer efficiency of photogenerated electrons and the catalytic reaction rate. This results led to the rapid and efficient degradation of MB and RhB. The system demonstrated superior catalytic performance and excellent reusability compared to other materials from the same period, showing great potential for application in dye wastewater treatment.

Lee et al. employed carbonization and activation technology to treat ZIF‐8 particles, resulting in a high specific surface area (1412.8 m^2^ g^−1^) and rich micro/mesoporous structures. These particles were then combined with NC hydrogel to prepare porous composite microbeads (Figure 15c).^[^ ^149^ ^]^ The nanopores generated during the activation process significantly enhanced the material's adsorption capacity for dye molecules, promoting adsorption through various interactions such as van der Waals forces, π‐π stacking, and hydrogen bonding. The hydrophilic environment and mechanical support provided by the NC hydrogel improved the dispersibility and stability of the ZIF‐8 particles, making the particularly effective in the recycling of dye molecules such as RhB. The composite microbeads demonstrated excellent dye adsorption performance in a fixed‐bed continuous treatment system, especially in the removal of RhB, showcasing excellent industrial applicability. Yang et al. constructed a high‐porosity, structurally stable MOFs composites by in situ growing UiO‐66 and UiO‐66‐NH_2_ on modified cellulose sponge (MCS) (Figure 15d).^[^ ^59^ ^]^ The 3D porous network of MCS not only provided chelation sites for Zr^4^⁺ but also ensured the structural integrity of the material in complex water environments through excellent mechanical properties and water resistance. The material performed well in MB cycling experiments. Additionally, the amino functional groups of UiO‐66‐NH_2_ enhanced electrostatic and hydrogen bonding interactions with MB molecules, significantly improving the adsorption capacity.^[^ ^162^ ^]^ Ebrahimi‐Koodehi et al. prepared Ni/Mn‐MOF via the reflux method and grew it on BC.^[^ ^163^ ^]^ The resulting Ni/Mn‐MOF@BC achieved an 84% degradation efficiency for MB under visible light within 3 h. Yan et al. designed a composite nanofiber material (BC@MOF) based on BC and MOF using an in situ growth method. They successfully prepared a self‐supporting porous membrane (BMMCa) with a high specific surface area (651 m^2^ g^−1^) and demonstrated excellent reusability in multiple cycles of filtration and adsorption of congo red (CR) (Figure 15e).^[^ ^164^ ^]^ BMMCa exhibited superior performance in treating wastewater containing anionic or cationic dyes due to hierarchical porous structure, good fluid permeability, MOF‐host interactions, and the salting‐out effect of Ca^2^⁺. The multiple intermolecular interactions between BC@MOF and CR were presented by molecular dynamics simulation and visualization, including hydrogen bonds, π‐π stacking, electrostatic interactions. Based on this, the adsorption capacity of CR reached 3518.6 mg g^−1^.

**Table 5:** The data related to the application of MOFs‐NC composites for dye adsorption and degradation are presented.

NC	MOFs	Metal	Organic linkers	Pollutants	Initial conditions	Kinetics models	Isotherms types	Adsorption capacity/efficiency	Mechanism	Refs.
CNC	ZIF‐8	Zn	2‐MIM	MB	10 mg L^−1^	PSO dynamic	Type I, Type IV	99.80%	Porous structure, hydrophilic	[148]
				MG	100 mg L^−1^			1060.2 mg g^−1^	Porous structure, Hydrophilic, π‐π conjugation	
BC	ZIF‐8	Zn	2‐MIM	MB	20 mg L^−1^, visible light	PFO dynamic	/	100.00%	Photocatalysis, enzyme catalysis, electron transfer	[58]
				RhB	20 mg L^−1^, visible light			96.10%		
CNF	ZIF‐8	Zn	2‐MIM	RhB	10–2000 mg L^−1^, 293 K	PSO dynamic	Langmuir model	99.00%	Hydrophobic interactions, π‐π interactions, van der Waals forces, hydrogen bonds	[149]
CNF	UiO‐66	Zr	PTA	MB	20–1300 mg L^−1^, 25 °C, pH 7	PSO dynamic	Freundlich model	294.7 mg g^−1^	Electrostatic interaction, hydrogen bond	[59]
	UiO‐66‐NH_2_	Zr	H_2_BDC‐NH_2_					400.9 mg g^−1^		
BC	Ni/Mn‐MOF	Mn Ni	H_2_BDC	MB	10 ppm, 7502 W lamp, source distance: 10 cm	PFO dynamic	/	84%	Separation and transfer of photo‐generated carriers, free‐radical mechanism, separation of electrons and holes	[163]
BC	Zr‐MOF	Zr	H_3_TCA	CR	5–300 mg L^−1^, 25 °C	PSO dynamic	Langmuir model	3518.6 mg g^−1^	Hydrogen bond, π‐π stacking, coordinate bond, electrostatic interaction, molecular size sieving	[164]

### 4.2 Pharmaceutical Removal

Pharmaceutically active compounds (PhACs) including antibiotics, antidepressants, hormones, and analgesics, are commonly found in water bodies and pose significant environmental concerns due to toxicity, bioaccumulation, and resistance to degradation.^[^ ^165^ ^]^ MOFs‐NC composites offer a promising solution to this issue thanks to low toxicity, excellent reproducibility, and environmentally friendly characteristics. The removal mechanisms of pharmaceutical contaminants are broadly similar to those of dyes, involving the synergistic effects of adsorption, π‐π interactions, and photocatalytic degradation.^[^ ^158^, ^166^ ^]^ However, in contrast to dyes, which typically possess higher molecular weights and complex aromatic structures, pharmaceutical molecules are generally smaller and contain a greater number of polar functional groups.^[^ ^167^ ^]^ These groups enhance the propensity of pharmaceutical compounds to engage in chemisorption and electrostatic interactions with functional moieties on the surface of MOFs‐NC composites. Due to their smaller molecular size, pharmaceuticals often require longer diffusion times and more gradual formation of chemical bonds during actual application. In addition to radical‐mediated oxidative degradation, pharmaceutical molecules can also degrade through specific chemical pathways. Hydrophilic surface modifications enhance this process by promoting chemisorption and electrostatic interactions.^[^ ^155^ ^]^

**Figure 16:**
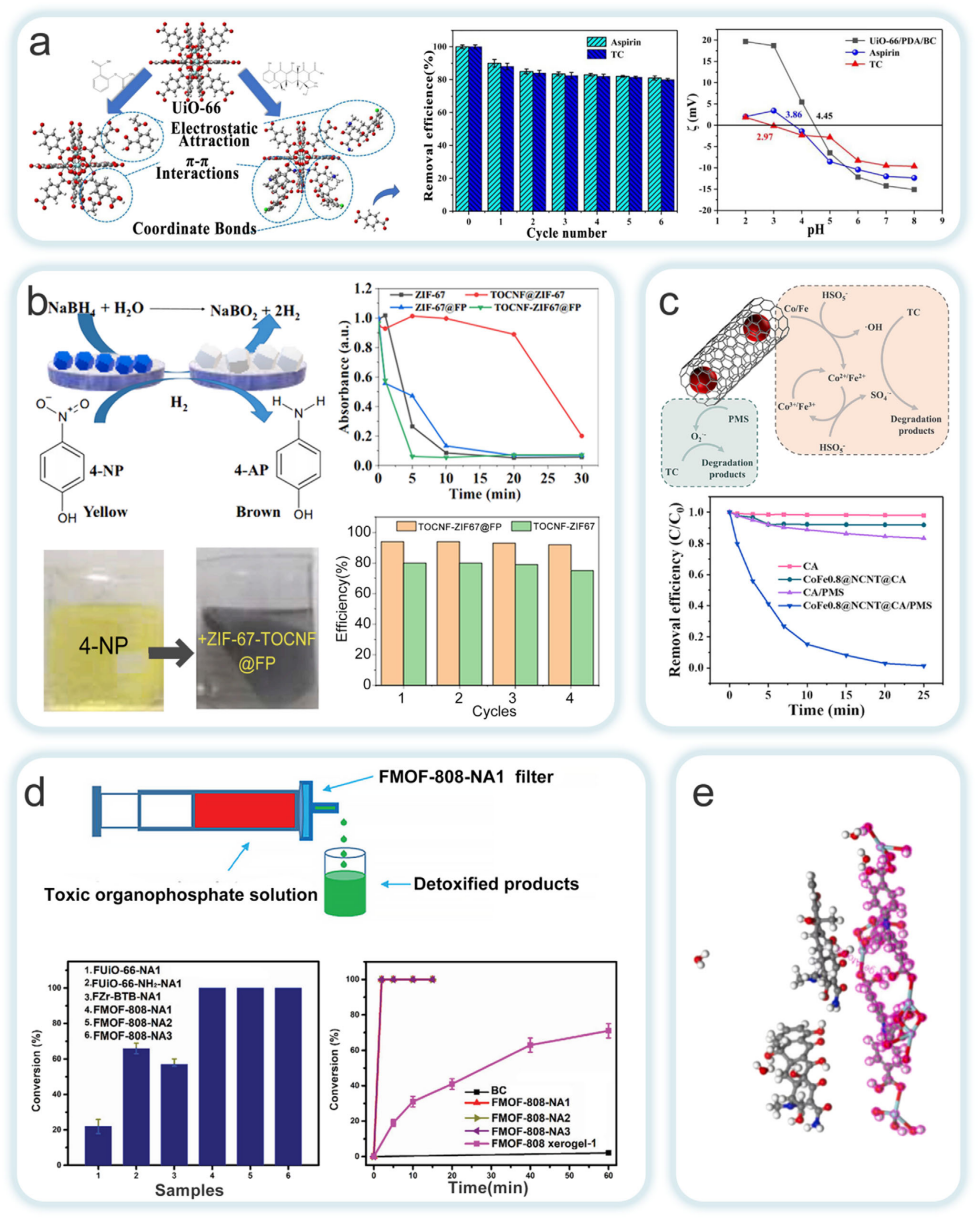
a) The adsorption mechanism, cycling performance, and zeta potential of UiO‐66/PDA/BC under different pH conditions. Reproduced with permission.^[^ ^53^ ^]^ Copyright 2020, Elsevier B.V. b) Degradation mechanism, efficiency, circulation performance of ZIF67‐TOCNF@FP. Reproduced with permission.^[^ ^169^ ^]^ Copyright 2021, Elsevier Ltd. c) Degradation mechanism, efficiency of CoFe@NCNT@CA. Reproduced with permission.^[^ ^170^ ^]^ Copyright 2022, Elsevier. d) Schematic representation of the continuous hydrolysis of toxic organophosphate nerve agent simulant reactor with FMOF‐808‐NA1. Efficiency and cycle performance of FMOF‐808‐NA1. Reproduced with permission.^[^ ^173^ ^]^ Copyright 2023, Wiley‐VCH GmbH. e) BMAT_3_H_5_‐water‐ TC conffgurations by MS simulations. Reproduced with permission.^[^ ^175^ ^]^ Copyright 2025, Elsevier Ltd.

The following content primarily emphasizes the adsorption and degradation mechanism of MOFs‐NC composites for drug molecules (**Table** **6**). Cui et al. used BC as a scaffold to prepare a soft foam composite of UiO‐66/polydopamine/BC (UiO‐66/PDA/BC) with a 3D network structure and high hydrophilicity (**Figure** **16**a).^[^ ^53^ ^]^ The uniform coating of UiO‐66 nanoparticles ensured a high surface area and provided active sites for adsorbing target pollutants.^[^ ^168^ ^]^ The removal efficiency of aspirin and TC remained above 83% after six cycles. Further investigation into the adsorption mechanism revealed that the adsorption behaviors for aspirin and TC were different. The adsorption of aspirin was mainly physical adsorption, which was controlled by weak intermolecular interaction, and the chemical environment of Zr had no obvious change. In contrast, TC adsorption involved electrostatic interactions, π–π interactions, and the potential formation of new Zr‐N coordination bonds, leading to a stronger adsorption effect., The electrostatics promoted adsorption in the pH range of 3 to 4, which was below the isoelectric point, while the opposite occured when the pH increased to 5 or higher. However, the presence of other interactions mitigated this effect such as hydrogen bonding or π‐π interactions, which significantly broadening the practical application range of the adsorbent. 4‐Nitrophenol (4‐NP), a hazardous pollutant with high resistance to biodegradation, posed a significant threat to human health. Abdelhamid et al. proposed a rapid and cost‐effective wet chemical method to prepare ZIF‐67 with TOCNF on Whatman® Filter Paper (ZIF67‐TOCNF@FP) (Figure 16b),^[^ ^169^ ^]^ achieving high reduction efficiency in a short reaction time. The ZIFs are known for high biocompatibility and cost‐effectiveness. By using TOCNF as a template and growing crystals around the TOCNF molecules embedded in the cellulose fibers of the filter paper, the structural properties of the material were significantly enhanced. However, the alkalinity of TOCNF limited the adsorption performance of 4‐NP in the TOCNF‐ZIF‐8 system, which was weaker than that of ZIF‐8‐based materials. The chemical reduction of 4‐NP was visually confirmed by a color change from yellow to brown, and the material showed no significant degradation after multiple recycling cycles.

Wu et al. proposed a composite aerogel (CoFe@NCNT@CA) containing bimetallic MOF‐derived carbon nanotubes (CNTs) integrated into a cellulose aerogel for the activation of peroxymonosulfate (PMS) to degrade TC (Figure 16c).^[^ ^170^ ^]^ The aerogel exhibited excellent catalytic performance, achieving a 97.1% degradation rate of TC within 20 min. This attributed to the synergistic effects of its porous network structure, bimetallic catalytic sites, and graphite nitrogen (N) structure. The presence of CoO and FeO facilitated the reversible redox reactions between Co^3^⁺/Co^2^⁺ and Fe^3^⁺/Fe^2^⁺, providing catalytic activity to activate PMS.^[^ ^171^ ^]^ The high Co^2^⁺ content further promoted the redox reactions, enhancing the electron conversion efficiency. Additionally, the coexistence of graphite N‐characterized by high electronegativity, Co and Fe single metal particles accelerated electron transfer between the metal components and PMS, further promoting the redox process. Furthermore, the semi‐encapsulated structure formed by the MOF‐derived CNT and cellulose aerogel not only exposed a large number of active sites but also effectively enhanced CNT adhesion.^[^ ^172^ ^]^ This contributing to the strong stability and excellent regeneration of the CoFe@NCNT@CA/PMS system. Ma et al. developed a rapid synthesis method for preparing a processable monolithic aerogel composite with high MOF loading by introducing a Zr‐MOF nanozyme coating into CNF (Figure 16d).^[^ ^173^ ^]^ The low‐connectivity Zr nodes in the MOF‐808 structure provided abundant catalytic active sites.^[^ ^174^ ^]^ Moreover, the large pore size facilitates the diffusion of substrates and products between the catalytic sites. The hierarchical pore structure formed by the macroporous CNF aerogel matrix and microporous Zr‐MOF nanozyme effectively enhanced access to MOF‐based catalytic sites. The composite aerogel demonstrated excellent performance in the catalytic hydrolysis of the organophosphorus nerve agent simulant DMNP and the pesticide dichlorvos. Regeneration could be achieved through simple water washing, indicating significant practical application potential. Additionally, the rapid gelation method was adaptable to various Zr‐MOF. And different functions can be introduced by changing the precursor, providing valuable insights for further development of environmental remediation materials.

Yan et al. developed a bifunctional aerogel microsphere (BMATXHY) based on NC and Zr‐MOF for efficient fluorescent detection and rapid adsorption removal of TC in water.^[^ ^175^ ^]^ Molecular simulations revealed π‐π stacking and hydrogen bonding interactions between TC and BMAT_3_H_5_, as well as the bridging role of water molecules, which collectively enhanced the adsorption stability of TC (Figure 16e). These findings provide theoretical insights for understanding drug adsorption mechanisms and designing high‐performance adsorbent materials. Abd El‐Monaem et al. fabricated UiO‐66/ZIF‐8/PDA@CA composite materials in buoyant bead form.^[^ ^176^ ^]^ This innovative design achieved highly efficient TC removal while resolving the separation challenges of conventional MOF adsorbents through its inherent buoyancy properties. The composite demonstrated excellent reusability, providing an economically viable and highly effective solution for antibiotic removal from aquatic environments.

**Table 6:** The data related to the application of MOFs‐NC composites for pharmaceutical removal are presented.

NC	MOFs	Metal	Organic linkers	Pollutants	Initial conditions	Kinetics model	Isotherms types	Adsorption capacity/efficiency	Mechanism	Refs.
BC	UiO‐66	Zr	PTA	Aspirin	20–200 mg L^−1^ 25 °C	PSO dynamic	Langmuir model	141.1 mg g^−1^	Electrostatic interactions, π–π stacking	[53]
TC	20–200 mg L^−1^ 25 °C	184.3 mg g^−1^	Chemical adsorption
TOCNF	ZIF‐8	Zn	2‐MIM	4‐NP	1 µg mL^−1^ NaBH_4_ as reducing agent	/	/	/	/	[169]
ZIF‐67	Co	2‐MIM	4‐NP	1 µg mL^−1^ NaBH₄ as reducing agent	/	/	92–94%	Electron transfer mechanism, free radical mechanism, synergistic effect
CNF	CoFe‐MOF	Co Fe	HMTA‐DCA	TC	40 mg L^−1^ PMS environment	PFO dynamic	/	97.10%	Electron transfer mechanism, free radical mechanism, synergistic effect	[170]
CNF	MOF‐808	Zr	BTC	DMNP DDVP	25 µmol room temperature	/	/	100.00%	Catalytic hydrolysis, synergistic effect of pore structure	[173]
BC	Zr‐MOF	Zr	H_3_TCA	TC	2 µM–40 mg L^−1^ 25°C pH 7	PSO dynamic	Langmuir model	317.6 mg g^−1^ 92.10%	Hierarchical hole structure, π–π stacking, electrostatic interaction	[175]
CA	UiO‐66	Zr	BDC	TC	50–300 mg L^−1^, pH 7	PSO dynamic	Temkin model	290.69 mg g^−1^ 83.20%	π–π interactions, hydrogen bond, Coordination, electrostatic interaction	[176]
ZIF‐8	Zn	2‐MIM

### 4.3 Heavy Metal Ion Capture

Trace metals play a crucial role in the proper functioning of cellular biological processes. However, an excess of metal ions can bind to protein sites, disrupting normal cellular function.^[^ ^177^ ^]^ When metal ions accumulate through the food chain, they can induce biological toxicity in the human body, leading to diseases such as minamata disease,^[^ ^178^ ^]^ alzheimer's disease, and parkinson's disease.^[^ ^179^ ^]^ The surfaces of MOFs‐NC composites are enriched with negatively charged functional groups, such as carboxyl and hydroxyl, which enable the rapid capture of heavy metal ions (e.g., Pb^2^⁺, Cu^2^⁺) via electrostatic interactions.^[^ ^158^ ^]^ Introducing specific functional groups onto the MOFs‐NC composites surface can optimize surface charge characteristics and enhance selective metal adsorption. MOF metal nodes (e.g., Zr‐OH, Zn‐OH) can participate in ion‐exchange processes with heavy metals. For redox‐active metal species, photocatalytic reduction can convert highly toxic high‐valence metals into lower‐valence states, reducing toxicity and promoting precipitation. The 3D porous network constructed by NC not only prevents MOF agglomeration but also facilitates ion diffusion via its hierarchical porosity. Collectively, these features confer high adsorption capacity, selectivity, and regenerability, positioning MOFs‐NC composites as uniquely advantageous for heavy metal remediation.^[^ ^60^ ^]^

**Figure 17:**
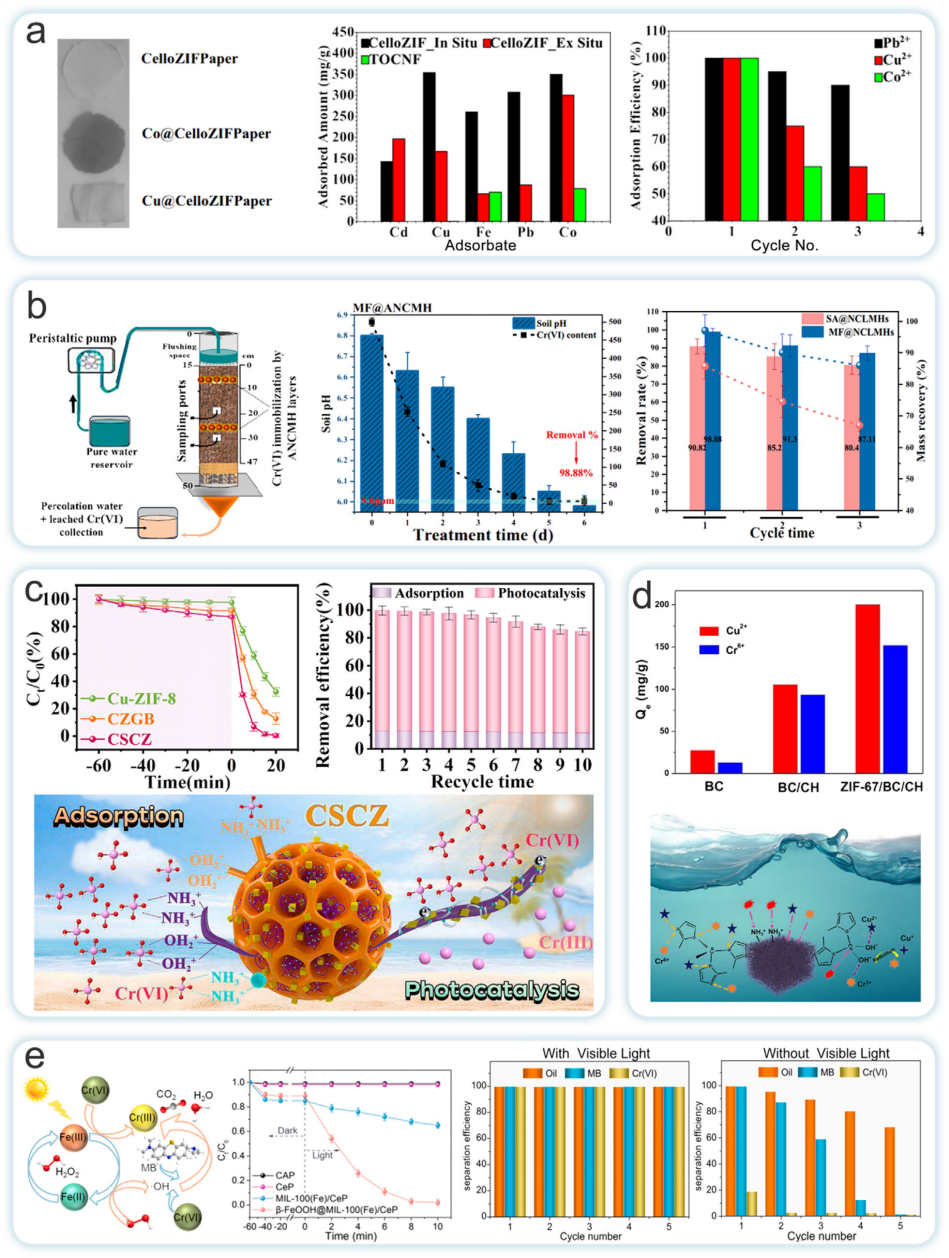
a) The optical photograph, adsorption capacity, and recycle performance for heavy metal ion by CelloZIFPaper. Reproduced with permission.^[^ ^131^ ^]^ Copyright 2022, Elsevier B.V. b) Schematic diagram of the device. Distribution patterns of Cr^6+^ concentrations in soils and the percentages of soil OM after different soil remediation treatments. Reproduced with permission.^[^ ^181^ ^]^ Copyright 2025, Elsevier B.V. c) Adsorption and photocatalysis performances, circulation performance, adsorption mechanism of CSCZ. Reproduced with permission.^[^ ^146^ ^]^ Copyright 2024, Elsevier B.V. d) Adsorption capacity and mechanism of ZIF‐67/BC/CH aerogels for Cu^2+^ and Cr^6+^. Reproduced with permission.^[^ ^54^ ^]^ Copyright 2020, Elsevier B.V. e) The adsorption and photocatalytic degradation mechanism and performance of Cr^6+^ and its recycle performance. Reproduced with permission.^[^ ^56^ ^]^ Copyright 2021, Elsevier.

The performance, mechanisms, and other effect of different MOFs‐NC composites for heavy metal ions as shown in **Table** **7**. Abdelhamid et al. synthesized CelloZIFPaper hybrid materials by loading ZIF‐8 crystals into cellulose pulp (CP) or TOCNF through in situ and ex situ methods, which can effectively adsorb heavy metal ions from water (**Figure** **17**a).^[^ ^131^ ^]^ The CP facilitated the adjustment of the TOCNF‐ZIF network structure and promoted the formation of flat plates. The incorporation of CP and TOCNF compensated for the insufficient functional groups in MOF, thereby enhancing metal ion adsorption capacity. The adsorption process occurred through coordination or electrostatic interactions between heavy metal ions and the carboxyl groups of TOCNF without ion exchange.^[^ ^180^ ^]^ However, tetrahedral ions such as Cu^2^⁺ or Co^2^⁺ coordinate with the functional groups of ZIF‐8 during the adsorption process, which resulted in relatively low recyclability for these ions. The as‐prepared CelloZIFPaper demonstrated an excellent adsorption capacity for heavy metal ions, reaching 345.0 mg g^−1^. Additionally, the preparation process was entirely water‐mediated, making the operation not only simpler but also more environmentally friendly. Shaghaleh et al. developed an innovative aminated MOFs‐NC hydrogel nanocomposite adsorbent for the sustainable remediation of agricultural soils contaminated with hexavalent chromium, in combination with soil flushing technology (Figure 17b).^[^ ^181^ ^]^ A‐CNFs, prepared via two‐phase air plasma/NaOH pretreatment and TEMPO oxidation, provided abundant amino groups that were protonated under acidic conditions, enhancing the adsorption capacity for Cr^6^⁺. The hydrogel's high swelling property enabled rapid absorption and released of water at different pH values, facilitating the adsorption and desorption of Cr^6^⁺ in soil environments. The 3D porous structure of ANCMH offered a large specific surface area, while the dense distribution of amino and carboxyl groups enhanced electrostatic adsorption and complexation of Cr^6^⁺.^[^ ^182^ ^]^ The theoretical maximum adsorption capacity of ANCMH was 338.24 mg g^−1^, with an adsorption efficiency of 98.9% at pH 6.8. After three adsorption‐desorption cycles, the removal rate of Cr^6^⁺ remained between 80.4% and 87.1%, demonstrating good regeneration ability. Zhang et al. proposed a chitosan/silver cluster‐loaded CNF/Cu‐ZIF‐8 gel bead (CSCZ) designed to mimic a spherical sponge structure for efficient removal of Cr^6^⁺ from water (Figure 17c).^[^ ^146^ ^]^ The SC@CN enhanced the surface area and N content of functional groups, providing more adsorption sites. The 3D porous structure of CSCZ enriched with surface functional groups such as amino (‐NH_2_) and hydroxyl (‐OH), which facilitated the adsorption of Cr^6^⁺ ions through electrostatic attraction and hydrogen bonding. The high specific surface area of Cu‐ZIF‐8 acted as a photocatalyst under UV irradiation, generating electron‐hole pairs. The electrons were captured by the amino groups in SC@CN and transferred to CSCZ, promoting the reduction of Cr^6^⁺ to Cr^3^⁺. Simultaneously, some electrons reacted with oxygen to generate •O_2_⁻, which further participated in the reduction process of Cr^6^⁺. The maximum adsorption capacity of CSCZ was 171.2 mg g^−1^. Moreover, it retained 84.9% of initial removal rate after 10 cycles of adsorption and photocatalytic experiments, indicating excellent stability and reusability.

Li et al. modified BC/chitosan aerogels to create a novel adsorbent based on ZIF‐67 (Figure 17d).^[^ ^54^ ^]^ The intrinsic high porosity of the aerogels was retained by incorporating ZIF‐67, which addressed the issue of pore structure reduction caused by the addition of chitosan. This modification resulted in ZIF‐67/BC/CH aerogels exhibiting a significantly higher specific surface area compared to BC/CH aerogels. The inclusion of BC also enhanced the mechanical properties of the composite aerogels, improving the reusability. The Cr^6^⁺ and Cu^6^⁺ removal rates remained at 72% and 81% of the initial values after five cycles of reuse, respectively. The ZIF‐67/BC/CH aerogels demonstrated strong potential for heavy metal ion adsorption, with Cu^6^⁺ and Cr^6^⁺ adsorption capacities reaching 200.6 mg g^−1^ and 152.1 mg g^−1^, respectively. However, in practical applications, the adsorption capacity may decline to varying degrees due to differences in water matrices. Lu et al. introduced a cellulose‐based electrospun nanofibrous membrane (ENM) with a core‐sheath structure and robust photocatalytic activity, capable of simultaneously and efficiently separating oil emulsions, degrading dyes, and reducing Cr^6^⁺ (Figure 17e).^[^ ^56^ ^]^ This ENM enhanced the absorption and utilization of visible light through the β‐FeOOH@MIL‐100(Fe) heterostructure, promoted electron‐hole separation, and rapidly degraded pollutants via the photo‐Fenton synergistic effect. Its exceptionally high MIL‐100(Fe) loading (78 wt%) and large specific surface area (1105 m^2^ g^−1^) provided abundant active sites. The porous, hydrophilic MIL‐100(Fe) and well‐dispersed β‐FeOOH nanorods contribute to the superior performance of ENM in underwater oil rejection, enrichment, and photocatalytic‐fenton synergies. This material achieved a Cr^6^⁺ removal efficiency of 99.7%. Moreover, the membranes remained largely intact after five filtration cycles under illumination thanks to the self‐cleaning capability of the photocatalytic β‐FeOOH@MIL‐100(Fe)/CeP ENMs, demonstrating strong potential for practical applications. Zhang et al. fabricated MCC/CS/ZIF‐8 hybrid nanofibers by combining electrospinning with an in situ growth method, which enhanced the structural and thermal stability of the nanofibers.^[^ ^183^ ^]^ The formation of a CuO‐ZnO heterojunction further improved the adsorption capacity and efficiency, achieving a maximum Cu^2^⁺ adsorption capacity of 204.08 mg/g.

**Table 7:** The data related to the application of MOFs‐NC composites for heavy metal ion capture are presented.

NC	MOFs	Metal	Organic linkers	Pollutants	Initial conditions	Kinetics model	Isotherms types	Adsorption capacity/efficiency	Mechanism	Refs.
TOCNF	ZIF‐8	Zn	2‐MIM	Cd^2^⁺	1000 ppm room temperature(23 ± 2°C)	/	Langmuir mode	143.0 mg g^−1^	Ion exchange, coordination, electrostatic	[131]
Co^2^⁺	350 mg g^−1^
Cu^2^⁺	354.0 mg g^−1^
Fe^3^⁺	260.8 mg g^−1^
Pb^2^⁺	307.3 mg g^−1^
A–CNFs	MIL‐100(Fe)	Fe	H_3_BTC	Cr^6^⁺	500 ppm pH 6.8	PSO dynamic	Freundlich model	338.24 mg g^−1^	Electrostatic adsorption, ion exchange, reduction reaction, coordination, pore diffusion	[181]
CNF	Cu‐ZIF‐8	Cu	2‐MIM	Cr^6^⁺	20 mg L^−1^ 25°C pH 2	PSO dynamic	Langmuir mode	171.2 mg g⁻^1^/97.1%	Electrostatic adsorption, chemical adsorption, reduction, electron transfer, adsorption‐photocatalytic synergistic effect	[146]
BC	ZIF‐67	Co	2‐MIM	Cu^2^⁺	1 g L^−1^ room temperature pH 6	PSO dynamic	/	200.6 mg g^−1^	Electrostatic adsorption, chemical adsorption, reduction, ion exchange, synergistic effect	[54]
Cr^6^⁺	152.1 mg g^−1^
CNF	MIL‐100(Fe)	Fe	H_3_BTC	Cr^6^⁺	10 mg L^−1^ room temperature pH 7	PSO dynamic	/	99.70%	Electrostatic adsorption, chemical adsorption, separation and transfer of photogenerated carriers, separation of electrons and holes, photo‐catalytic‐Fon synergistic effect	[56]
MCC	ZIF‐8	Zn	2‐MIM	Cu^2^⁺	4–100 mg L^−1^, pH 2–6, 0–600 min	PSO dynamic	/	204.08 mg g^−1^	Physical adsorption, chemical adsorption, Ion exchange reaction	[183]

### 4.4 Adsorption and Degradation of VOCs

As the primary precursor of photochemical smog and ozone formation, VOCs emissions pose significant risks to human health. It also contribute to environmental issues, including stratospheric ozone depletion, climate change, and plant degradation.^[^ ^184^ ^]^ The MOFs‐NC composites, owing to porous structure and functionalized surface, exhibit excellent adsorption performance for VOCs molecules, such as formaldehyde, benzene, and toluene.^[^ ^185^ ^]^ The underlying mechanisms encompass physical adsorption, chemical adsorption, photocatalytic degradation, thermal catalytic degradation, and their synergistic effects. These composites possess high specific surface area and porosity, offering numerous binding sites for VOCs molecules. The incorporation of functional groups (e.g., amino, carboxyl) enhances chemisorption capacity.^[^ ^186^ ^]^ For photocatalytic degradation, coupling MOFs with semiconductors (e.g., ZnO, TiO_2_) improves the separation efficiency of photogenerated carriers, thereby enhancing VOCs degradation. Thermal catalytic degradation leverages metal ions (e.g., Fe^2^⁺, Cu^2^⁺) in MOFs as active sites to facilitate VOCs oxidation. Moreover, MOFs‐NC composites can enrich VOCs at the catalyst surface through adsorption, thereby realizing synergistic effects between adsorption and catalytic degradation. By tailoring pore architecture and surface functionality, selective adsorption and degradation of specific VOCs species can be achieved. As shown in **Table** **8**, these mechanisms collectively enable MOFs‐NC composites to exhibit high efficiency, stability, and reusability in VOCs removal, offering new avenues for advanced VOCs abatement technologies.

**Figure 18:**
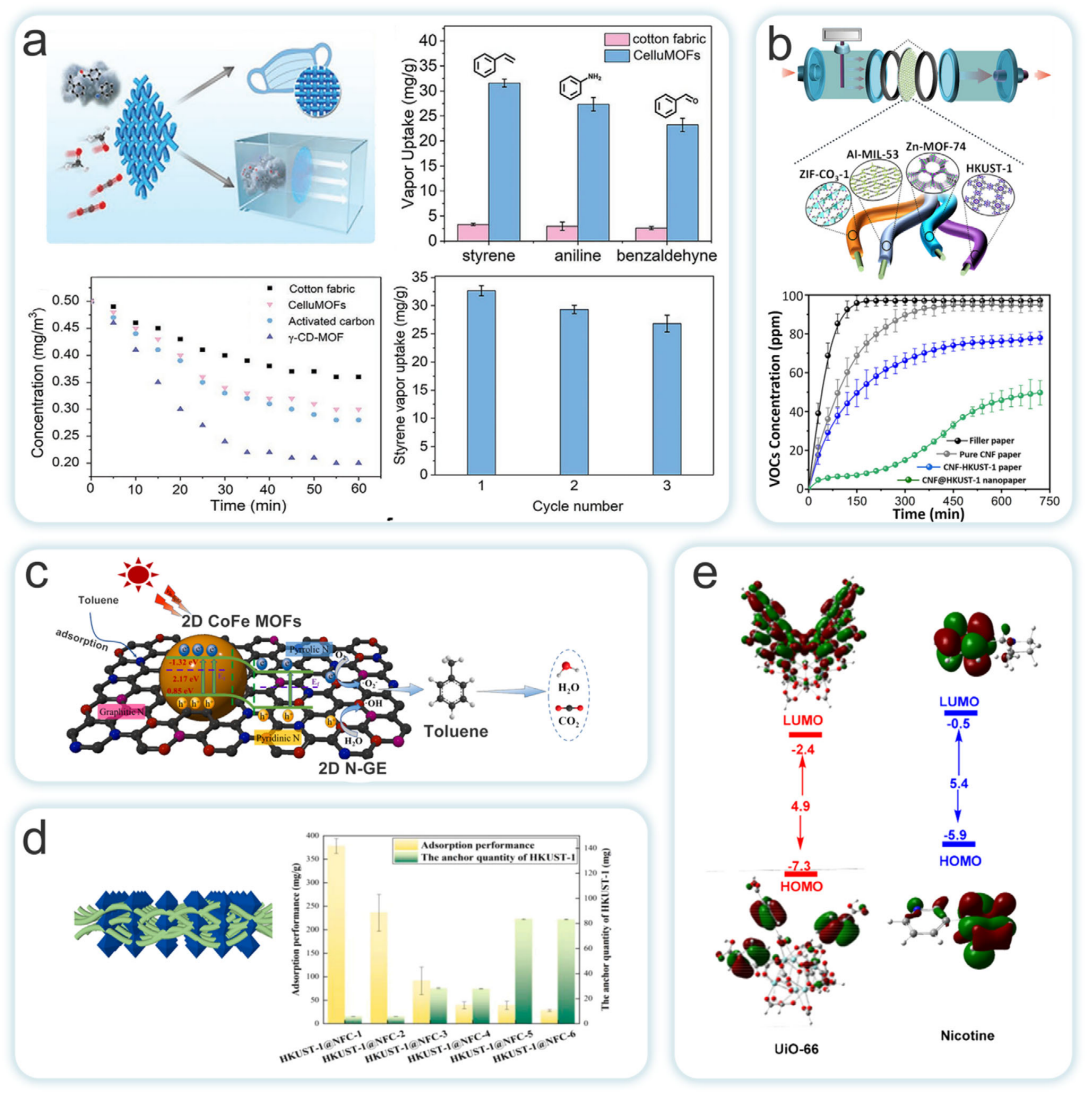
a) The schematic diagram, adsorption efficiency, and circulation performance of removing VOCs by CelluMOF. Reproduced with permission.^[^ ^187^ ^]^ Copyright 2021, Wiley‐VCH GmbH. b) The VOCs separation test device and adsorption performance of CNF@MOF nanopapers. Reproduced with permission.^[^ ^50^ ^]^ Copyright 2019, Wiley‐VCH GmbH. c) Photocatalytic degradation mechanism of CLGM‐A composites for toluene. Reproduced with permission.^[^ ^189^ ^]^ Copyright 2024, Elsevier. d) Schematic diagram of HKUST‐1 on the interior and the surface of the NFC. The formaldehyde adsorption performance of HKUST‐1 in the composite membranes. Reproduced with permission.^[^ ^65^ ^]^ Copyright 2022, Elsevier B.V. e) DFT calculation of the energy difference between HOMO and LUMO of UiO‐66. Reproduced with permission.^[^ ^191^ ^]^ Copyright 2023, ACS.

Since the chemical composition of TOCNF contains glucose molecules similar to γ‐cyclodextrin (γ‐CD), it can function as a coordination unit for complexation with potassium ions. Zhang et al. proposed a strategy to directly synthesize γ‐CD MOF on the surface of nanofibers, demonstrating the feasibility of this method for various cellulose fiber‐based materials (**Figure** **18**a).^[^ ^187^ ^]^ This approach avoids the use of toxic and hazardous reagents and harsh reaction conditions, as it does not require pretreatment. The results showed that the composites exhibited a VOCs adsorption capacity (for styrene, aniline, and benzaldehyde) ≈10 times greater than that of the original fiber (2.3–3.5 mg g^−1^), and retained a strong VOCs adsorption capacity over three cycles (using styrene as an example). Additionally, CelluMOF (with γ‐CD‐MOFs content ≈17 wt%) demonstrated significantly higher formaldehyde adsorption capacity compared to the original cellulose fiber, due to the strong hydrogen bonding and host‐guest interactions between the γ‐CD MOF and formaldehyde.^[^ ^188^ ^]^ Zhou et al. successfully synthesized four continuous MOFs layers (HKUST‐1, Al‐MIL‐53, Zn‐MOF‐74, and ZIF‐CO_3_‐1) on ultrafine CNFs by precisely controlling the charge density of CNFs and the amount of PVP via interfacial reactions. In a conceptual application for independent VOCs separation, CNF@MOF nanopaper was tested using formaldehyde as an example (Figure 18b).^[^ ^50^ ^]^ The results revealed that the CNF@HKUST‐1 nanopaper consistently maintained high separation efficiency throughout the experiment. This performance can be attributed to the assembly of interconnected nanostructures, achieved by wrapping CNFs with MOFs layers, as well as the synergistic effect of carboxylated CNFs and the PVP surfactant used in the synthesis. The low cost and simple preparation process of the hybrid CNF@MOF nanofiber enable large‐scale production and the creation of independent flexible nanopaper with hierarchical porosity, high transparency, high thermal stability, and high mechanical strength. This provides a pathway for the application of MOFs‐NC composites in VOCs separation.

Wang et al. developed a 3D composite aerogel catalyst (CLGM‐A) consisting of a CNF skeleton‐supported 2D CoFe bimetallic MOFs and a 2D N‐doped graphene (2D N‐GE) heterostructure via freeze‐drying. This composite was successfully applied to the photocatalytic degradation of toluene (Figure 18c).^[^ ^189^ ^]^ The heterostructure origined from the 2D/2D interface was characterized by close contact and abundant active sites, which formed a built‐in electric field, significantly altering the photogenerated carrier pathways. Rapid electron transfer and the suppression of electron backflow considerably improve the separation efficiency of photogenerated electron‐hole pairs.^[^ ^190^ ^]^ Simultaneously, N doping promoted the generation of more unsaturated metal‐O bonds in the graphene material (GM), enhanced internal electron transfer. Moreover, it also accelerated the separation of e/h+ pairs, thereby increasing the production of reactive oxygen species. In toluene degradation, O^2^⁻ radicals play a dominant role, while •OH facilitate VOCs degradation through oxidation and radical chain reactions. Meanwhile, h⁺ ions contribute by directly oxidizing VOCs molecules and generating •OH. Based on these effects, CLGM‐A exhibited a photocatalytic toluene conversion rate of 95.4% and a mineralization rate of 83.6% under visible light irradiation, showing outstanding stability and reusability in the corresponding continuous cycle test. Chen et al. prepared a composite membrane (HKUST‐1@NFC) by anchoring HKUST‐1 onto NFC at room temperature using a green, DMF‐free method (Figure 18d).^[^ ^65^ ^]^ This study revealed that the addition of HKUST‐1 significantly increased the specific surface area of the NFC membrane without affecting the properties of NFC. At high ligand concentrations, HKUST‐1 anchors both inside the membrane and on its surface. The resulting hierarchical porous structure exposes active adsorption sites, enhancing HKUST‐1's formaldehyde adsorption capacity per unit mass. In addition to the porous structure, the adsorption process of formaldehyde by the composite membrane was influenced by the electrostatic interaction between the positively charged copper ions on HKUST‐1 and the formaldehyde molecules. Under optimal conditions, the maximum formaldehyde adsorption capacity of the HKUST‐1@NFC‐1 composite membrane reached 378.09 mg g^−1^, significantly higher than that of unsupported HKUST‐1 powder. Furthermore, the composite membrane exhibited excellent regeneration performance. It maintained high formaldehyde adsorption capacity after five cycles, demonstrating its potential for application in formaldehyde adsorption.

Qu et al. integrated UiO‐66‐NH_2_ nanoparticles into PVA/CNF aerogels to develop a composite aerogel (CPU/A) with high hydrophobicity and superior adsorption performance.^[^ ^121^ ^]^ By optimizing the pore structure through adjusting the UiO‐66‐NH_2_ content, the material demonstrated excellent reusability in formaldehyde adsorption during multiple cyclic tests, highlighting its economic viability and sustainability. Wan et al. successfully developed a series of highly efficient nicotine adsorption materials (MOF@CF) by incorporating different types of MOFs with bamboo pulp cellulose fibers.^[^ ^191^ ^]^ Among these materials, UiO‐66@CF exhibited outstanding nicotine adsorption performance, achieving a removal efficiency of up to 90.07%. Density functional theory (DFT) calculations revealed that the HOMO‐LUMO energy gap of UiO‐66 (3.7 eV) was the closest to that of nicotine (4.9 eV), indicating a better electronic structure match between UiO‐66 and nicotine. This enhanced electronic compatibility contributes to its superior adsorption capacity (Figure 18e).

**Table 8:** The data related to the application of MOFs‐NC composites for adsorption and degradation of VOCs are presented.

NC	MOFs	Metal	Organic linkers	Pollutants	Initial conditions	Kinetics model	Adsorption capacity/efficiency	Mechanism	Refs.
CNF	γ‐CD‐MOF	K	γ‐CD	Styrene Aniline Benzaldehyde formaldehyde	/	/	22.5–32.7 mg g^−1^	Physical adsorption, hydrogen bonding or host–guest interactions	[187]
CNF	HKUST‐1	Cu	H_3_BTC	Formaldehyde	100 ppm N_2_ environment	/	/	Physical adsorption, chemical adsorption	[50]
	Al‐MIL‐53	Al	H_2_BDC						
	Zn‐MOF‐74	Zn	H_2_DOBA						
	ZIF‐CO_3_‐1	Zn	2‐MIM						
CNF	2D CoFe MOFs	Co Fe	/	Toluene	100 ppm illuminance:160 mW cm^−^^2^ source distance: 10 cm	PFO dynamic	95.40%	2D/2D heterostructures, separation and transfer of photo–generated carriers, free–radical mechanism	[189]
CNF	HKUST‐1	Cu	H_3_BTC	Formaldehyde	3.0 ± 0.2 mg m^−3^ 25°C static test mode	/	378.09 mg g⁻¹ 69.83%	Porous structure, charge interaction	[65]
CNF	UiO‐66‐NH_2_	Zr	NH_2_‐BDC	Formaldehyde	1.5 ppm	PFO dynamic	98 mg g^−1^ 98%	Physical adsorption, chemical adsorption	[121]
CNF	ZIF‐8	Zn	2‐MIM	Nicotine	0.8 mg 800 mL min^−1^ 0.035 MPa	/	84.70%	Coordination, hydrogen bond, electrostatic interaction	[191]
	HKUST‐1	Cu	BTA				82.45%		
	MOF‐74	Mg	Tph				78.78%		
	UiOs	Zr	Tph				86.30%–90.07%		

### 4.5 CO_2_ Capture and Separation

The capture and utilization of CO_2_ is a crucial strategy for achieving carbon neutrality.^[^ ^192^ ^]^ MOFs‐NC composites offer essential support for efficient adsorption and separation processes. The CO_2_ capture mechanism primarily involves surface modification and functionalization of NC, the intrinsic high surface area and porosity of MOFs, synergistic interactions between chemisorption and physisorption, as well as recyclability and cycle stability. Chemical modification of NC introduces functional groups such as amino and carboxyl groups that interact with CO_2_ via chemisorption to form stable chemical bonds, thereby improving capture efficiency. Simultaneously, the large surface area and porous nature of MOFs provide abundant adsorption sites, facilitating CO_2_ diffusion and uptake. The combination of NC and MOFs further enhances the synergistic effect between chemisorption and physisorption, improving overall performance and mechanical robustness. These composites can be regenerated through simple thermal or chemical treatments after CO_2_ adsorption, exhibiting excellent cyclic stability and offering economic and practical viability for real‐world applications (as shown in **Table** **9**).^[^ ^193^ ^]^

**Figure 19:**
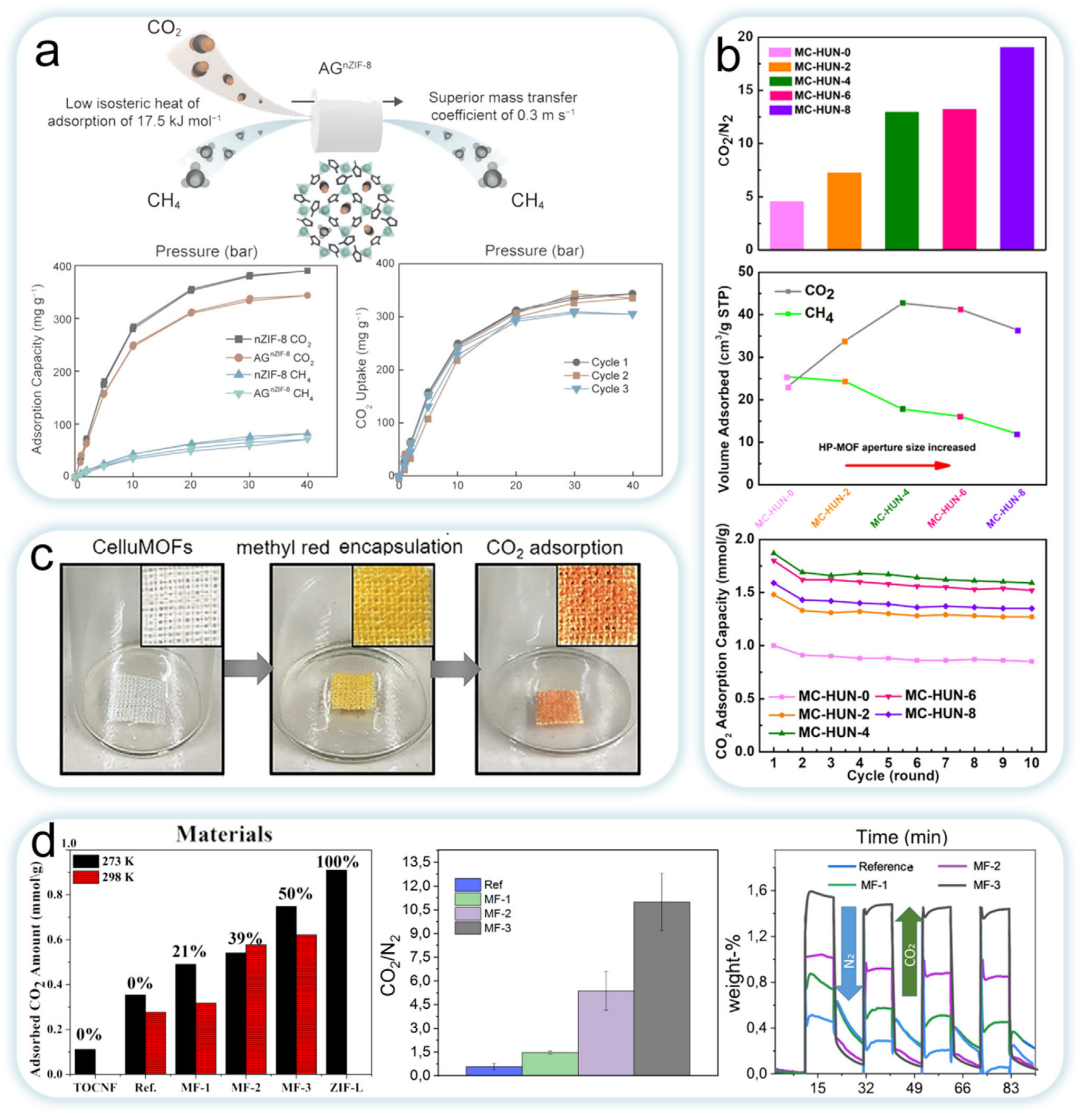
a) Schematic illustration of the selective adsorption of AGnZIF‐8 aerogel. Adsorption isotherms of CO_2_ and CH_4_ on AGnZIF‐8 aerogel at 293 K, and the cyclic CO_2_ adsorption isotherm. Reproduced with permission.^[^ ^139^ ^]^ Copyright 2022, Wiley‐VCH GmbH. b) Adsorption capacity, CO_2_/N_2_ selective adsorption, circulation performance of MC‐HUN‐X composites. Reproduced with permission.^[^ ^194^ ^]^ Copyright 2022, Springer. c) Schematic diagram of CO_2_ adsorption process of CelluMOFs. Reproduced with permission.^[^ ^187^ ^]^ Copyright 2021, Wiley‐VCH GmbH. d) Adsorption capacity, adsorption selectivity, cyclical of NC/ZIF‐L foams. Reproduced with permission.^[^ ^196^ ^]^ Copyright 2019, Elsevier.

To address the challenge of shaping MOF in powder form, Rostami et al. combined MOF with tailored, high‐aspect‐ratio cationic CNFs of the same charge to create strong, wet‐stable, multifunctional MOF‐based aerogels (**Figure** **19**a).^[^ ^139^ ^]^ These cationic CNFs form highly entangled networks even at low concentrations. This enables the ice‐templating method to create a parallel tubular pore structure aligned with the growth direction of ice crystals, with the pore walls coated in ZIF‐8 particles, enhancing the material's directional physical properties. This anisotropic feature facilitates gas flow through the MOF, promoting gas filtration, adsorption, and separation. Due to the strong interaction between CO_2_ molecules and the ZIF‐8 structure, the aerogel exhibits a higher adsorption capacity for CO_2_ than CH_4_, reaching up to 343 mg g^−1^, and demonstrates superior performance in CO_2_/CH_4_ gas separation. Unlike chemical adsorption in zeolites, CO_2_ is physically adsorbed in AGnZIF‐8, and its adsorption capacity remains stable under high pressure, maintaining good structural stability. In a similar study, Yu et al. combined cellulose aerogels with hierarchical porous MOFs (HP‐MOFs) to explore the effect of pore size on CO_2_ adsorption performance in hybrid aerogels (Figure 19b).^[^ ^194^ ^]^ By adjusting the chain length of monocarboxylic acid (MA) to modify the pore size, the researchers observed that CO_2_ adsorption increased with larger pore sizes. The adsorption capacity initially rose, peaked at 0.90 mmol g^−1^ in the mesoporous state, and then declined. This behavior is attributed to the larger pore size facilitating CO_2_ transfer to the interior of HP‐MOFs and improving contact with internal active sites, while also restricting coordination between metal ions and BDC‐NH_2_.^[^ ^195^ ^]^ Furthermore, HP‐UiO‐66, containing amino groups (‐NH_2_), reacted with CO_2_ to form carbamates and hydrogen bonds. The strong interaction between its active sites and CO_2_ molecules further enhances CO_2_ adsorption. The CO_2_ adsorption capacity retains 85% of its original value after ten cycles.

Zhang et al. prepared a composites of CelluMOFs for CO_2_ capture and adsorption by in situ growing γ‐CD MOFs on microcrystalline cellulose (MC) aerogel (Figure 19c).^[^ ^187^ ^]^ MC provided flexibility and environmental friendliness, while the synthesized MOFs impart the required porosity and unique chemical properties to the material. γ‐CD‐MOFs had large hydrophilic pores and capture CO_2_ by forming carbonates with the hydroxyl groups of γ‐CD. In the experiment, a CelluMOFs sample containing methyl red was exposed to a CO_2_ environment, and its color changed from yellow to light orange. This color change indicated that CO_2_ reacted with components in γ‐CD‐MOFs, enabling the capture and adsorption of CO_2_. The CelluMOFs (containing ≈17 wt.% γ‐CD‐MOFs) adsorbed up to 4.93 cm^3^ g^−1^ of CO_2_ under low‐pressure conditions (*P*/*P*₀ = 0.001). During the first adsorption and desorption cycle, some active sites in the composite were occupied by N_2_, resulting in a significant decrease in adsorption capacity. However, the material still demonstrated excellent reusability in multiple CO_2_ cycling experiments. Valencia et al. synthesized a NC leaf‐like zeolitic imidazole framework (ZIF‐L) foam at room temperature using water as the solvent (Figure 19d).^[^ ^196^ ^]^ Compared to the tetrahedral structure of other ZIFs, ZIF‐L crystals feature large 0D pores or cavities that facilitate CO_2_ adsorption via a gate‐opening mechanism.^[^ ^197^ ^]^ In multiple CO_2_ cycling experiments, the adsorbent exhibited high adsorption capacity, selectivity, long‐term stability, and excellent reusability. Furthermore, the CO_2_ weight gain remaining largely unaffected in repeated CO_2_/N_2_ cycles.

Gan et al. innovatively composited MIL‐100(Fe) with surface‐carboxylated cellulose nanocrystals (OCN) and incorporated the hybrid into polyvinyl alcohol (PVA) aerogel.^[^ ^198^ ^]^ The resulting MIL‐100(Fe)@OCN/PVA aerogel demonstrated a CO_2_ adsorption capacity of 0.357 mmol/g while maintaining biodegradability, showcasing its potential as a lightweight and environmentally friendly CO_2_ capture material. Muhamed et al. achieved excellent interfacial integration between HKUST‐1 and NC through a copper ion pretreatment and carboxylate anchoring approach.^[^ ^122^ ^]^ This interfacial integration significantly enhanced the composite material's performance in CO_2_/N_2_ gas separation. Compared to standalone MOF, the composite demonstrated ≈300% improvement in CO_2_/N_2_ adsorption selectivity.

**Table 9:** The data related to the application of MOFs‐NC composites for CO_2_ capture and separation are presented.

NC	MOFs	Metal	Organic linkers	Initial conditions	Isotherms types	Adsorption capacity/efficiency	Mechanism	Refs.
CNF	ZIF‐8	Zn	2‐MIM	CO_2_/CH_4_ = 50mol/50mol 293 K 40 bar	/	22.5–32.7 mg g^−1^	Strong interaction	[139]
CNC	HP‐UIO‐66‐NH_2_	Zr	BDC‐NH_2_	298 K 1 bar	Type I	50%	Physical adsorption, chemical adsorption, pore size effect, selective adsorption	[194]
CNF	γ‐CD‐MOF	K	γ‐CD	room temperature sealed	/	95.4%	High specific surface area and porous structure, host–guest interaction, chemical adsorption, van der Waals force	[187]
TOCNF	ZIF‐L	Zn	2‐MIM	0°C 1 bar	Type I	378.09 mg g⁻¹/69.83%	Gate control mechanism, synergistic effect, hierarchical porous structure	[196]
OCN	MIL‐100(Fe)	Fe	H_3_TBC	0.216 g cm^−^^3^	/	0.357 mmol g^−1^	Micro‐pore adsorption, hierarchical hole structure	[198]
Na@NC	HKUST‐1	Cu	BTC	298 K 1 bar CO₂/N₂ = 15/85 (v/v)	Langmuir model	2.7 mmol g^−1^	Interface engineering, interaction	[122]

### 4.6 PM Separation

In addition to reducing atmospheric visibility, fine particulate matter (PM2.5) can penetrate deep into the alveoli and bloodstream, causing cardiovascular and respiratory diseases.^[^ ^199^ ^]^ The deposition of particulate matter can also disrupt plant photosynthesis and contaminate water and soil. MOFs‐NC materials serve as highly efficient agents for PM removal through the synergistic actions of physical interception, electrostatic adsorption, and molecular sieving. Their multilayered architecture and hierarchical porosity enable stepwise capture of PM. The NC fiber network provides structural support, prevents MOFs aggregation, and ensures full exposure of active sites. The incorporation of MOFs enhances surface charge and polarity, facilitating the electrostatic adsorption of charged particles. Moreover, the uniform microporous structure of MOFs allows for size‐selective adsorption of targeted species. These characteristics collectively endow MOFs‐NC composites with superior performance in PM filtration and remediation (as shown in **Table** **10**).^[^ ^60^ ^]^

**Figure 20:**
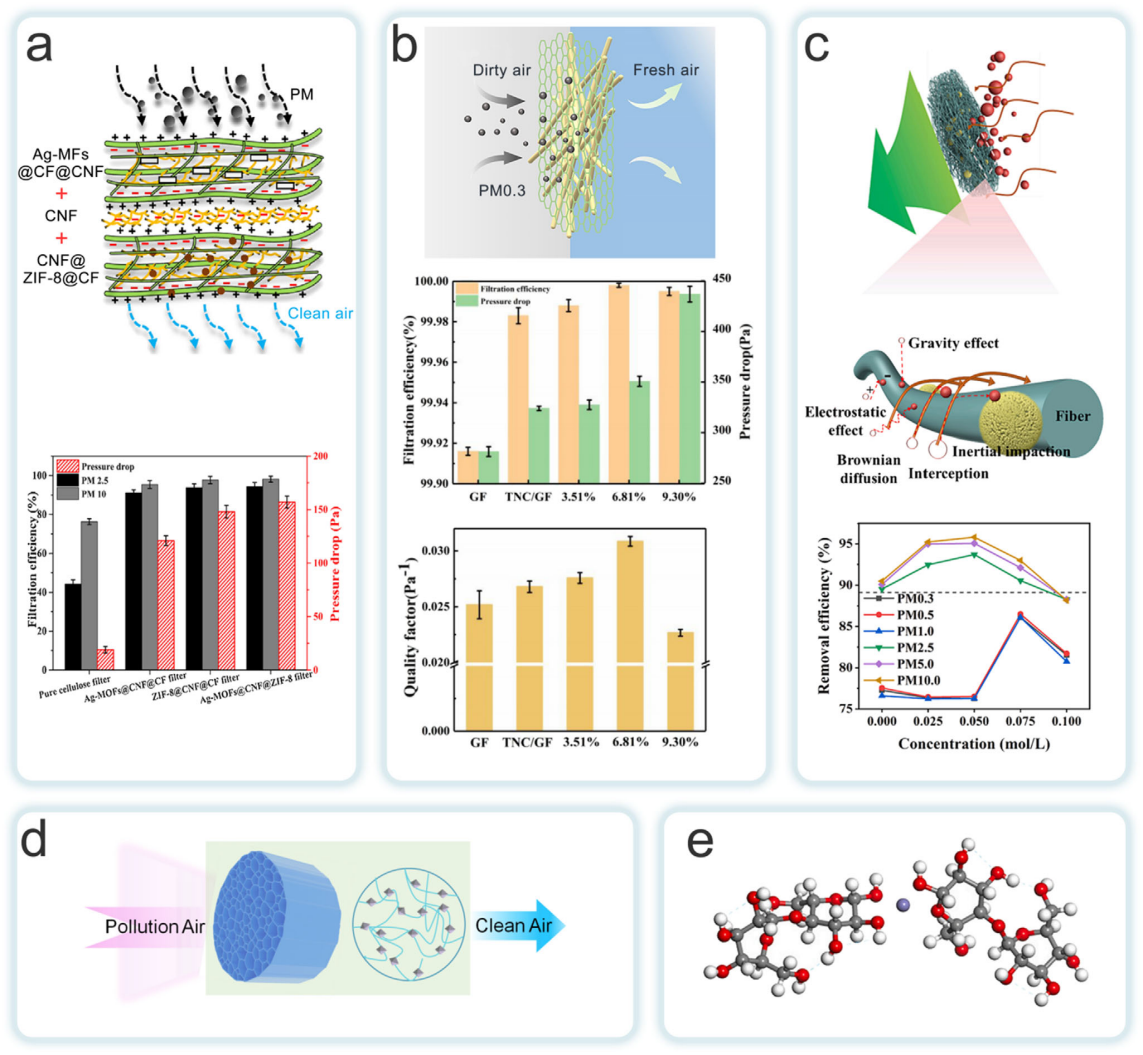
a) The filtration mechanism diagram and the filtration performance of Ag‐MOFs@CNF@ZIF‐8 filter. Reproduced with permission.^[^ ^51^ ^]^ Copyright 2019, Elsevier. b) Application diagram, filtration efficiency, pressure drop and quality factor of the ZIF‐8@TNC/GF membrane. Reproduced with permission.^[^ ^55^ ^]^ Copyright 2021, ACS. c) Schematic diagram of the filtration mechanism of ZIF‐8@TOCNFs aerogel and its adsorption effect on different PM sizes. Reproduced with permission.^[^ ^202^ ^]^ Copyright 2023, Elsevier B.V. d) Illustration of filtration mechanism of MIL‐125‐NH_2_@BCH. Reproduced with permission.^[^ ^203^ ^]^ Copyright 2022, Elsevier. e) DFT calculations of strong interfacial interactions between NH_2_‐MIL‐101(Fe) and the cellulose matrix. Reproduced with permission.^[^ ^205^ ^]^ Copyright 2016, ACS Publications.

Ma et al. designed a biodegradable air filter (Ag‐MOFs@CNF@ZIF‐8) reinforced by a two‐component MOFs structure and CNF (**Figure** **20**a).^[^ ^51^ ^]^ This unique multilayer structure enhanced particle interception through a gradient filtration method, increasing the effective collision frequency between the filter and PM. The MOFs, with high specific surface area and rich pore structure, increased the contact area and interaction opportunities between the filter and PM. The polar surface of Ag‐MOFs can polarize PM particles, promoting interaction with the unbalanced metal ions in the MOFs.^[^ ^200^ ^]^ Additionally, the MOF deposition improved the pore structure of the filter, making it more complex and dense, which further promoted PM capture. Moreover, the CNF acted as a bridging agent and formed strong hydrogen bonds with cellulose fibers, creating a layered structure that strengthens the material's mechanical properties. In a separate study, Huang et al. reported a high‐efficiency air filtration material consisting of a 3D network structure of core‐shell ZIF‐8‐coated sea squirt NC (TNC)/glass fiber (GF) for PM0.3 (fine particulate matter) removal (Figure 20b).^[^ ^55^ ^]^ This material utilized a rich pore structure and large specific surface area (50.3 m^2^ g^−1^) to enhance adsorption and interception of various PM types. The core‐shell structure minimized TNC aggregation, further increasing porosity and specific surface area. Notably, the surface defects of ZIF‐8 nanocrystals can polarize PM particles^[^ ^200^, ^201^ ^]^ and enhanced the electrostatic interactions between the particles and ZIF‐8, thereby improving PM capture efficiency. With the optimal ZIF‐8 ratio, the filtration efficiency of the ZIF‐8@TNC/GF membrane for PM0.3 reached 99.998%. At a TNC concentration of 0.0015 wt%, the filtration efficiency of the TNC/GF air filter was maximized due to the formation of a 2D nanostructure network between the glass fibers, which also results in the highest quality factor and a lower pressure drop.

Wang et al. proposed a non‐MOF template strategy that facilitated the heteroepitaxial growth of ZIF‐8 on TOCNF extracted from Chlamydomonas sp. (Figure 20c).^[^ ^202^ ^]^ The high aspect ratio (>1000) and the negatively charged carboxyl and hydroxyl groups of TOCNF made it an excellent template for promoting the heterogeneous nucleation of ZIF‐8 crystals, resulting in a unique porous structure resembling blood cells. The ZIF‐8@TOCNF aerogels exhibited outstanding porosity, high specific surface area and distinct porous structure, which facilitated superior performance in the removal of various PM. Through mechanisms such as interception, inertial impaction, brownian diffusion, gravity, and electrostatic effects, the aerogel achieved a PM removal efficiency of up to 95.80% at an air flow velocity of 0.34 m s^−1^. Hao et al. integrated titanium‐based MOFs (MIL‐125 and MIL‐125‐NH_2_) into BC/chitosan foams to prepare an air filter (Figure 20d).^[^ ^203^ ^]^ A special freeze‐drying design was used to construct an interconnected porous structure in the vertical direction of the composite foam, while an ordered parallel structure was formed along the axis. This anisotropic structure reduced the resistance encountered by the gas during transport. When simulated PM‐containing polluted air passed through the BCH air filter, particles were captured by the foam matrix through diffusion, interception, and inertial impact. The aligned channels provided high permeability for the air filter, while the addition of MIL‐125‐NH_2_ enhanced the specific surface area and hierarchical porous structure. Furthermore, the amino functional groups of MIL‐125‐NH_2_ chemically adsorb and form hydrogen bonds with PM particles,^[^ ^200^, ^204^ ^]^ while the positive charge on its surface attracts and captures negatively charged PM particles through electrostatic interactions. The filtration efficiency of the MIL‐125‐NH_2_@BCH filter for PM2.5 exceeds 99.5%, and the filtration efficiency consistently remained above 99.0% in stability tests. Liu et al. enhanced PM removal efficiency by depositing NH_2_‐MIL‐101(Fe) on a cellulose substrate.^[^ ^205^ ^]^ The resulting NH_2_‐MIL‐101@cellulose composite filter demonstrated filtration efficiencies of 95.8% for PM10 and 92.3% for PM2.5. DFT calculations confirmed strong interfacial interactions between NH_2_‐MIL‐101(Fe) and the cellulose matrix (Figure 20e).

**Table 10:** The data related to the application of MOFs‐NC composites for PM separation are presented.

NC	MOFs	Metal	Organic linkers	Pollutants	Initial conditions	Isotherms	Adsorption capacity/efficiency	Mechanism	Refs.
CNF	Ag‐MOFs	Ag	H_3_BTC	PM2.5 PM10	28°C flow rate:32 L min^−1^ 40% RH	Type I	94.30%	Physical adsorption, chemical adsorption, electrostatic adsorption, multilayer network, hydrodynamic effect	[51]
	ZIF‐8	Zn	2‐MIM						
TNC	ZIF‐8	Zn	2‐MIM	PM0.3	28°C flow rate:32 L min^−1^ surface wind speed:5.3 cm s^−1^ effective test area:100 cm^2^ 40% RH	Type I	100.00%	Porous structure, —3D network structure, fluid dynamics effect, chemical adsorption, group functionalization, electrostatic adsorption	[55]
TOCNF	ZIF‐8	Zn	2‐MIM	PM0.3	airflow velocity:0.34m s^−1^	/	95.80%	Interception and inertial impact, sliding stream effect	[202]
				PM2.5			93.70%		
				PM5.0			95.06%		
				PM10			95.80%		
BC	MIL‐125	Ti	H_2_BDC	PM2.5	constant wind speed: 2 m s^−1^	/	92.60%	Physical adsorption, electrostatic interaction, functional group effect, physical interception, gas diffusion	[203]
	MIL‐125‐NH_2_		2‐NH_2_‐BDC				99.50%		
CNF CNC	NH_2_‐MIL‐101(Fe)	Fe	NH_2_‐BDC	PM2.5	999 µg m^−^^3^ Wind speed: 2 m s^−1^	/	92.3%	Physical interception, electrostatic interaction, chemical adsorption, hierarchical hole structure	[205]
				PM10			95.8%		

### 4.7 Other Application

In addition to effectiveness in treating pollutants such as dyes, drugs, heavy metal ions, VOCs, CO_2_, and PM, MOFs‐NC composites also demonstrate significant potential for the removal of other pollutants. The detailed data of various researches of MOFs ‐ NC composites for other pollutants are presented in **Table** **11**.

**Figure 21:**
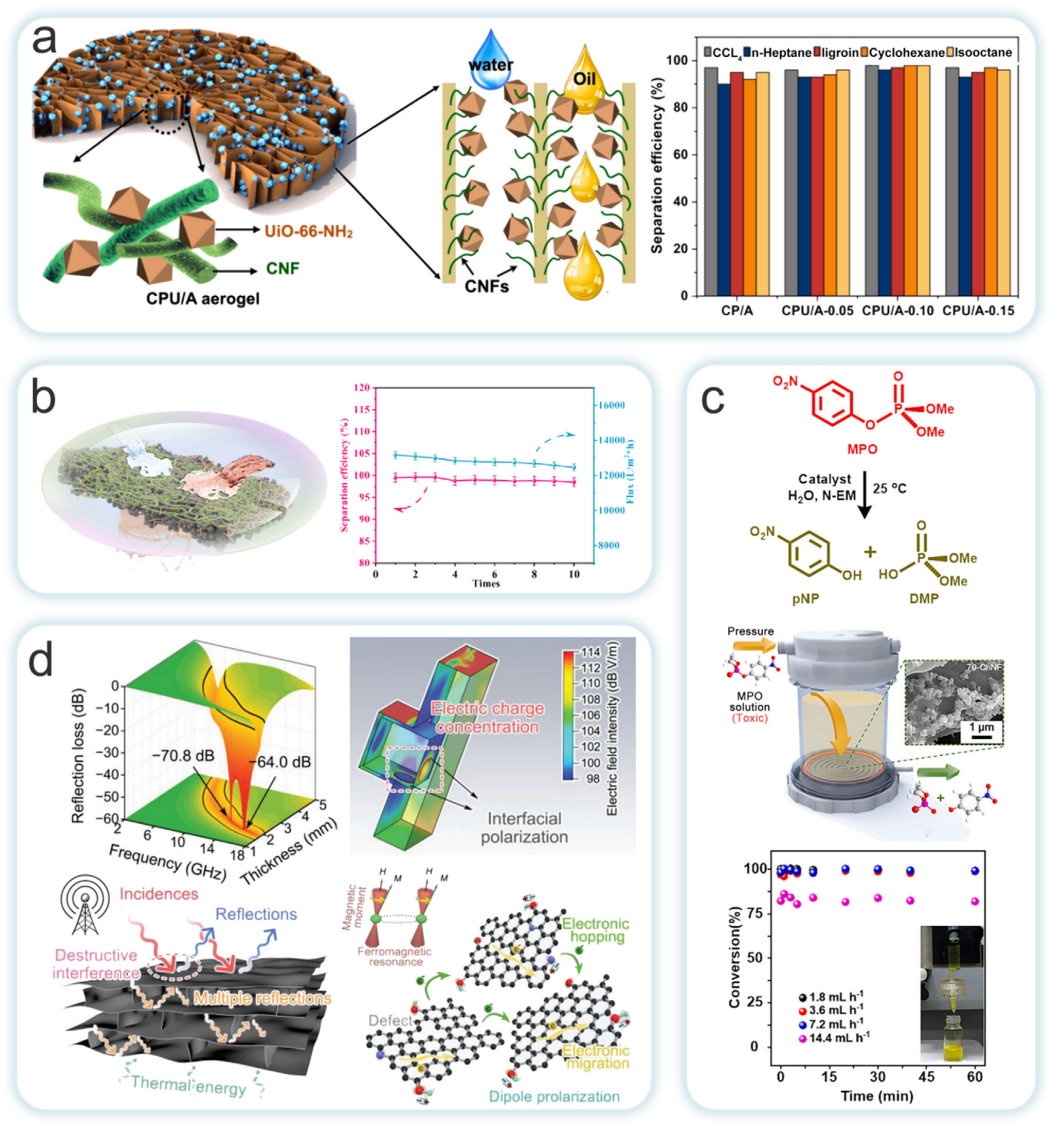
a) Structure, mechanism of oil/water mixture separation efficiency of CPU/A aerogel. Reproduced with permission.^[^ ^121^ ^]^ Copyright 2023, Elsevier B.V. b) The schematic diagram of oil–water separation, and the variation of separation efficiency and flux with time for water/trichloromethane solution. Reproduced with permission.^[^ ^207^ ^]^ Copyright 2023, Elsevier B.V. c) The illustration of continuous phase detoxification of CWA simulants by the ChNF/UiO‐66 reactive membrane, and the change in conversion rate over time at different flow rates. Reproduced with permission.^[^ ^135^ ^]^ Copyright 2024, Elsevier. d) 3D Reflection Loss (RL) curves and electric field distribution schematic illustrations of EWA mechanisms for the CoFe/carbon aerogels, electric field distribution of local CoFe@carbon nano‐capsules. Reproduced with permission.^[^ ^140^ ^]^ Copyright 2024, Wiley‐VCH GmbH.

Oil pollution is prevalent in marine spills, industrial wastewater, and accidental leaks, causing long‐term and often irreparable damage to aquatic ecosystems.^[^ ^206^ ^]^ For instance, Qu et al. developed a highly hydrophobic aerogel with enhanced oil–water separation performance by incorporating UiO‐66‐NH_2_ into a polyvinyl alcohol (PVA)/CNF composite aerogel (**Figure** **21**a).^[^ ^121^ ^]^ The modified CNF/PVA@UiO‐66‐NH_2_‐Alkylated composite aerogel (CPU/A) exhibited pleasurable absorption capacity for various oils and organic solvents. This is attributed to the interconnected pores formed by directional freezing, which facilitate liquid absorption and provide substantial storage space. The CNF formed a robust network structure with a high specific surface area when combined with CPU/A, which imparting the aerogel with excellent mechanical stability and flexibility. The flexible, elastic structure of the aerogel allowed for subsequent extraction and recovery of the adsorbed oil, which achieving effective oil–water separation. The CPU/A showed exceptional reusability in oil–water separation, with separation flux maintaining 98% of the original flux after 10 cycles. Additionally, Si et al. proposed a sustainable method to fabricate ultralight, high‐porosity, and hydrophobic double‐network aerogels (Figure 21b).^[^ ^207^ ^]^ Physical entanglement created a stable network structure during the preparation process, while freeze‐drying controlled the size, distribution of the pores, and ensuring high porosity. Chemical vapor deposition (CVD) with methyltrimethoxysilane (MTMS) resulted in a water contact angle of 132.6°, which enhancing the hydrophobic properties and improving oil adsorption capacity. The aerogel demonstrated an adsorption capacity ranging from 35.99 to 74.55 g g^−1^ for oil, with a separation efficiency for CHCl_3_ exceeding 98.5% after 10 cycles. The aerogel can remove oil under gravity alone without internal structure collapse and without requiring additional energy input, indicating that has strong potential for practical applications.

Due to the persistent presence of chemical warfare agents (CWAs) in the environment and the high toxicity caused widespread harm. Meanwhile, handling these substances remains challenging. Seo et al. successfully developed an environmentally friendly reaction membrane, which containing amine‐functionalized chitin nanofibers (ChNF) and UiO‐66 for efficient decomposition of CWA simulants in both static and continuous flow systems (Figure 21c).^[^ ^135^ ^]^ A significant amount of UiO‐66 catalyst (over 70 wt%) was selectively deposited on the continuous pores of ChNF. Moreover, the Lewis‐acidic Zr_6_(µ_3_‐O)_4_(µ_3_‐OH)_4_ clusters provided numerous catalytic active sites for reactions with organophosphate CWAs. The membrane maintained porosity while interacting through strong hydrogen bonds and metal‐amine coordination. The 3D nanoporous structure of the ChNF aerogel facilitated rapid diffusion of reactants and maintained structural stability.^[^ ^208^ ^]^ This special performances led to excellent catalytic activity for organophosphate decomposition. Additionally, the Brønsted basic functional groups (─NH_2_ and ─NHCOCH_3_) in ChNF synergized with the Lewis acidic sites of UiO‐66 to enhance CWA hydrolysis by promoting proton transfer,^[^ ^208^, ^209^ ^]^ while imparting anti‐fouling properties. Since the active sites of UiO‐66 can be regenerated after use, the catalyst is reusable in multiple cycles, enabling sustainable treatment of CWAs. The 70‐ChNF composite achieved 100% conversion of the CWA analog methyl paraphosphinate (MPO) within 3 min. Furthermore, the kinetic coefficient (k) decreased of only 10% after five repeated MPO hydrolysis tests, which demonstrating excellent reusability.

With the widespread use of wireless communication devices and power systems, electromagnetic wave pollution has garnered increasing attention due to its potential threat to the ecological environment and human health. Qiao et al. successfully synthesized highly dispersed and well‐connected MOF‐derived magnetic nanocapsules within carbon aerogels, significantly improving the electromagnetic wave shielding performance (Figure 21d).^[^ ^140^ ^]^ This high‐quality interface promoted close contact between CoFe nanocapsules and the carbon matrix, which enhancing interfacial polarization relaxation and providing an additional pathway for electromagnetic loss. By simulating the distribution of the electric field, it was found that the electric field was concentrated at the interface, which proved that the interface polarization and relaxation process played an role in the attenuation of electromagnetic waves. The high dispersion and strong interfacial connectivity of CoFe nanocapsules in the carbon sheet combined with the biomimetic ordered porous morphology, optimize impedance matching. This synergy also facilitates the entry of electromagnetic waves into the aerogel. Simultaneously, the hierarchical microstructure induces multiple reflections extending the wave transmission path and promoting dissipation. In addition to good conductivity and magnetic loss capability, the dispersion and interfacial connectivity of CoFe nanocapsules are improved. Consequently, interfacial polarization relaxation is enhanced, enabling multiple electromagnetic attenuation mechanisms. The material exhibited an expanded effective absorption bandwidth of 6.0 GHz, covering the frequency range of 4.3 to 18 GHz. It achieved an outstanding reflection loss value of −70.8 dB at a remarkably low filling rate of 2.2 wt.% and surpassing all currently reported carbon‐based aerogel absorbers. The Mai team fabricated HMN composite films with unique alternating electromagnetic structures through an alternating vacuum‐assisted filtration method.^[^ ^154^ ^]^ In the same year, they designed a bilayer aerogel structure (BZMN) and proposed a distinctive “absorption‐reflection‐reabsorption” mechanism.^[^ ^210^ ^]^ Both innovative structural designs significantly enhanced the materials' electromagnetic shielding performance while simultaneously endowing them with photothermal conversion capabilities. The photothermal conversion properties not only improved the material stability but also enabled thermal expansion‐induced nanostructural modifications that increased electromagnetic wave propagation paths within the material. Furthermore, the generated thermal energy could excite electrons within the material, thereby enhancing both electrical conductivity and magnetic permeability‐resulting in superior electromagnetic wave absorption performance.

**Table 11:** The data related to the application of MOFs‐NC composites for other application are presented.

NC	MOFs	Metal	Organic linkers	Pollutants	Initial conditions	Adsorption capacity/efficiency	Mechanism	Refs.
CNF	UiO‐66‐NH_2_	Zr	NH_2_‐BDC	Oil‐water mixture	/	100.00%	Hydrophobicity, porous structure, elastic structure	[121]
CNF	ZIF‐8	Zn	2‐MIM	Oil–water mixture	/	98.50%	Superhydrophobicity, high porosity	[207]
ChNF	UiO‐66	Zr	H_2_BDC	MPO	MPO/N‐EM = 0.025 mmol/0.45 m static water hydrolysis N‐EM as buffer	100.00%	Porous structure and electrostatic action, chemical adsorption, hydrolysis mechanism	[135]
					MPO/N‐EM = 0.5 mmol/0.45 m continuous flow constant flow rate:1.8 – 14.4 mL h^−1^	85%–100%		
CNF	PBA	Co Fe	Fe(CN)_6_^3^⁻	Electromagnetic wave	2–18 GHz	Min. refl. loss:–70.8 dB Spec. refl. loss: –1450 dB mm⁻¹	Interface polarization–relaxation, magnetic loss mechanism	[140]
TOCNF	ZIF‐8	Zn	2‐MIM	Electromagnetic wave	8.2–40.0 GHz	66.8 dB	Interface polarization–relaxation, magnetic loss mechanism, multiple scattering, solar thermal conversion	[154]
	ZIF‐67	Co	2‐MIM	0.1–4.0 THz	114.6 dB		
TOCNF	ZIFs	Co Ni	2‐MIM	Electromagnetic wave	8.2–12.4 GHz	21.2 dB	Interface polarization–relaxation, magnetic loss mechanism, multiple scattering, solar thermal conversion	[210]

## 5 Conclusion and Perspectives

### 5.1 Conclusion

**Figure 22:**
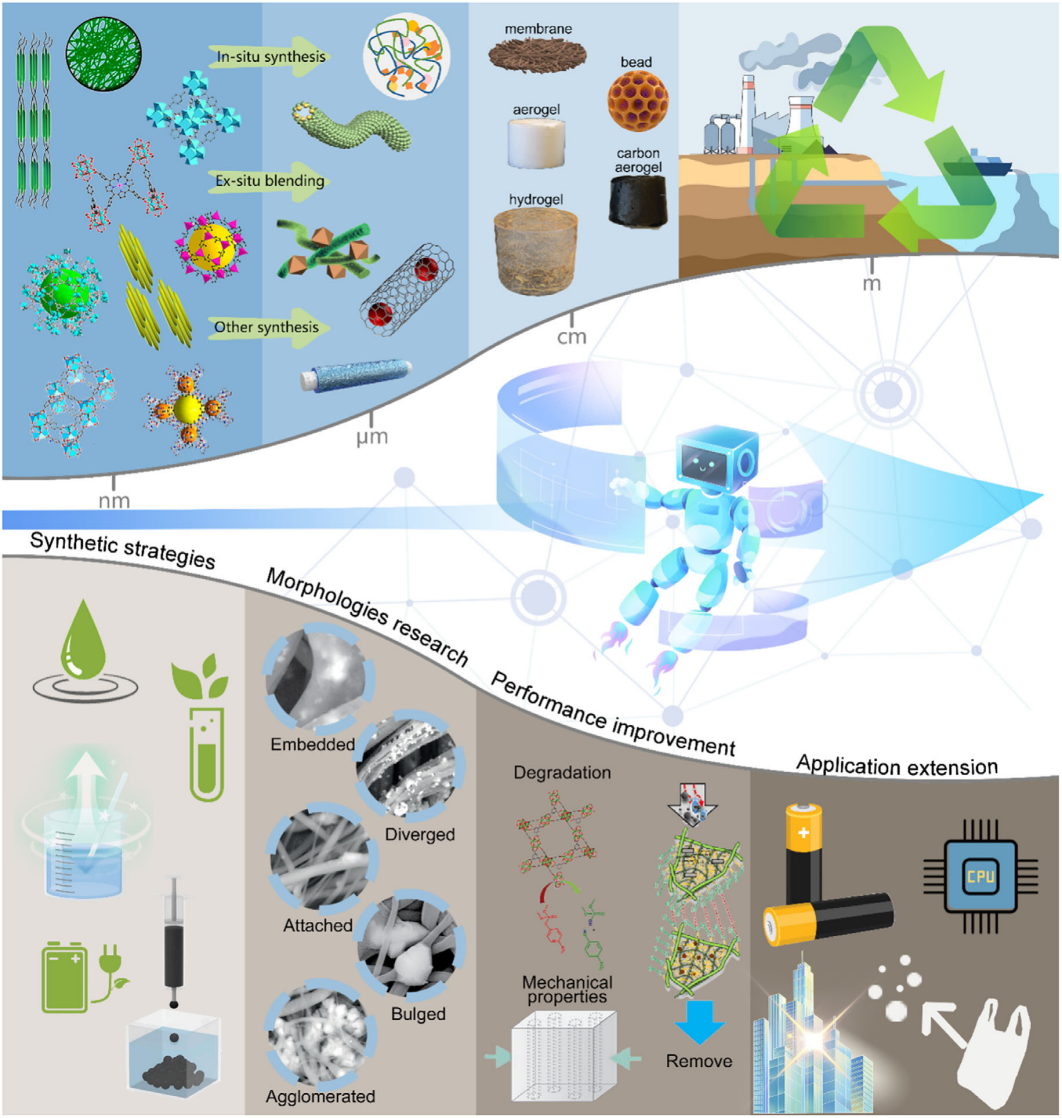
Outlooks of nanoarchitectonics of MOFs‐NC composites for multifunctional environmental remediation.

In summary, this review highlights the recent advances and application potential of MOFs‐NC composites in various environmental remediation domains (**Figure** **22**). The discussion begins with an overview of the materials microstructures and primary fabrication strategies, including both in situ and ex situ synthesis, followed by an analysis of the structure‐property‐function relationships. Subsequently, we summarize the applications and underlying mechanisms of MOFs‐NC composites in areas such as dye adsorption and degradation, pharmaceutical removal, heavy metal ion capture, adsorption and degradation of VOCs, CO_2_ capture and separation, and PM separation. Compared to pure NC and pure MOFs, MOFs‐NC composites exhibit superior structural, performance, and application advantages. However, research in this area is still in its early stages, and numerous challenges remain for practical implementation.

### 5.2 Perspectives

The critical challenges of MOFs‐NC composites for multifunctional environmental remediation are focused on follows (Figure 22):

1. Expanding MOFs structural diversity and establishing theoretical design frameworks. Current studies on MOFs‐NC composites are primarily focused on classic MOFs families such as ZIFs and MILs, which restrict the development of multifunctional systems. It is essential to establish universal structure‐activity relationship models and utilize high‐throughput screening and machine learning to systematically investigate the compatibility between MOFs topologies (e.g., pcu, fcu) and NC surface characteristics.
2. Elucidating interaction mechanisms between NC and MOFs. A comprehensive interfacial interaction map should be constructed to distinguish and analyze the synergistic or competitive roles of hydrogen bonding, electrostatic forces, and coordination interactions. Special attention should be given to the mechanisms governing selective coordination bond formation. Drawing on Peterson et al.’s classification of MOFs‐polymer fiber composite morphologies into embedded, divergent, adherent, protruding, and aggregated types, this framework can be extended to design cellulose‐based hybrid architectures.^[^ ^211^ ^]^
3. Enhancing the performance of MOFs‐NC composites. By adjusting the NC loading ratio in MOFs, the adsorption, degradation, and other properties of MOFs‐NC composites can be optimized. This adjustment also enhances material defects and improves their repair performance in practical applications.
4. Constructing closed‐loop intelligent remediation systems. Currently, MOFs‐NC composites have achieved continuous improvement in single functions such as adsorption and degradation. However, their performance remains limited in complex and variable real‐world pollutant systems. Although a basic “adsorption–degradation” remediation system has been established, there is still a lack of an intelligently regulated, real‐time remediation system. By using responsive MOFs‐NC composites as the input, a synergistic modular system integrating “detection + adsorption/degradation/catalysis” can be constructed to achieve more efficient and intelligent environmental remediation. Additionally, incorporating functions such as recycling and energy supply can further enhance green remediation.
5. Scaling up material production. The trade‐off between performance and cost remains a major barrier to commercializing MOFs‐NC composites. Future research should focus on optimizing morphology, performance, and efficiency. Implementing continuous‐flow chemistry systems can improve production efficiency and reduce batch variability. For powder‐form MOFs, developing effective post‐processing techniques to convert them into more user‐friendly and processable forms is equally important.
6. Establishing standardized application frameworks for industrial translation. To accelerate the standardization and commercialization of MOFs‐NC composites, a comprehensive application framework is needed. This includes defining performance benchmarks and functional descriptors for different MOFs and NC types, and tailoring application guidelines to meet the specific demands of diverse environmental sectors. Such standardization will provide clearer datasets and technical guidance, greatly enhancing the industrial viability of these materials.
7. Integration with materials informatics. Through computational models, machine learning, and artificial intelligence, materials informatics (MI) can rapidly process and analyze data from the preparation and application processes of MOFs‐NC composites. Simulating and predicting the performance of materials in real‐world environments enables more targeted optimization of synthesis conditions. This approach enhances material quality and accelerates their transition from the laboratory to the market.

Achieving these goals will provide significant support for the use of MOFs‐NC composites in environmental remediation. As technology advances, the preparation strategy and performance of MOFs‐NC composites will continue to improve. This will enable the full utilization of the properties of NC and MOFs. We look forward to seeing MOFs‐NC composites play an increasingly prominent role in this highly regarded field.

## Conflict of Interest

The authors declare no conflict of interest.
